# OST-HTH: a novel predicted RNA-binding domain

**DOI:** 10.1186/1745-6150-5-13

**Published:** 2010-03-19

**Authors:** Vivek Anantharaman, Dapeng Zhang, L Aravind

**Affiliations:** 1 National Center for Biotechnology Information, National Library of Medicine, National Institutes of Health, Bethesda, MD 20894, USA

## Abstract

**Background:**

The mechanism by which the arthropod Oskar and vertebrate TDRD5/TDRD7 proteins nucleate or organize structurally related ribonucleoprotein (RNP) complexes, the polar granule and nuage, is poorly understood. Using sequence profile searches we identify a novel domain in these proteins that is widely conserved across eukaryotes and bacteria.

**Results:**

Using contextual information from domain architectures, sequence-structure superpositions and available functional information we predict that this domain is likely to adopt the winged helix-turn-helix fold and bind RNA with a potential specificity for dsRNA. We show that in eukaryotes this domain is often combined in the same polypeptide with protein-protein- or lipid- interaction domains that might play a role in anchoring these proteins to specific cytoskeletal structures.

**Conclusions:**

Thus, proteins with this domain might have a key role in the recognition and localization of dsRNA, including miRNAs, rasiRNAs and piRNAs hybridized to their targets. In other cases, this domain is fused to ubiquitin-binding, E3 ligase and ubiquitin-like domains indicating a previously under-appreciated role for ubiquitination in regulating the assembly and stability of nuage-like RNP complexes. Both bacteria and eukaryotes encode a conserved family of proteins that combines this predicted RNA-binding domain with a previously uncharacterized domain (DUF88). We present evidence that it is an RNAse belonging to the superfamily that includes the 5'->3' nucleases, PIN and NYN domains and might be recruited to degrade certain RNAs.

**Reviewers:**

This article was reviewed by Sandor Pongor and Arcady Mushegian.

## Findings

Animal germ cells possess specialized ribonucleoprotein (RNP) granules termed nuage or germinal granules that appear as electron-dense fibrous and motile bodies localized to the cytoplasm [1-3]. In several animals they contain maternal determinants for germ cell specification and are asymmetrically partitioned into the future germline precursors. In contrast, despite the presence of germinal granules in cells of the mammalian germline, there is no evidence for such asymmetric partitioning [3]. Irrespective of their asymmetrical partitioning, the nuage complex contains several proteins that are conserved throughout animal evolution. These include well-known RNA-binding proteins such as nanos, Sm heptamers, hnRNPs, maelstrom with a catalytically inactive 3'->5' exonuclease domain and certain ribosomal proteins [4]. Additionally, these bodies contain processing and effector enzymes of the miRNA and piRNA systems, such as dicer, argonautes and piwis, components of the mRNA decapping complex and ATP-dependent RNA re-structuring enzymes such as the helicase Vasa [3,5]. Germinal bodies appear to perform many distinct roles: By storing mRNAs they might allow localization and stage-specific expression of certain transcripts. The miRNAs and piRNAs present in them play a central role in post-transcriptional gene silencing, transposon repression and perhaps regulation of DNA methylation [3,5]. Tudor domains present in several nuage complex proteins have been proposed to bind methylated arginines on modified Piwi proteins like aubergine, or to bind lysines on histone H3 and might be required for sequestering proteins with different covalent modifications [6,7]. Mutations or gene-deletions of several nuage components in mammals and arthropods result in male sterility and genomic disruption due to uncontrolled transposase activity [3,5]. Thus, these RNP complexes appear to be a critical complex for maintenance of germ line integrity. It is also possible that the nuage complex is structurally related to other major RNP granules such as those found in neurons [3]. In oocytes of dipteran insects there is a second RNP complex, the polar granule, which is related to the nuage complex, both ultrastructurally and in terms of its protein components [8]. Like the nuage complex of certain animals, polar granules are also asymmetrically distributed to the posterior pole of oocytes concentrated in the germplasm. However, it is not entirely clear if the nuage complex directly gives rise to polar granules or they are assembled independently from similar precursor proteins [8].

Nucleation of such RNP complexes has been extensively studied in *Drosophila*, where the RNA-binding protein Oskar has been found to be a critical player in initiating the formation of polar granules [1,8,9]. In spite of other pervasive similarities between the polar granules and the nuage complex, Oskar is believed to have no vertebrate homolog, unlike other conserved components of the nuage such as Vasa, Nanos and Tudor. Furthermore, components such as Tudor and Nanos appear to be largely restricted to metazoans; however, preservation of germline integrity and counter-transposon defense are more general issues faced by most eukaryotic lineages. Hence, we were interested in understanding if there were any common determinants and cognate processes involved in nucleation of nuage-like RNPs within metazoa and if such elements might be more widely distributed over the tree of Life. Using sensitive sequence profile analysis we show that Oskar and the vertebrate nuage complex proteins TDRD5 and TDRD7 share a conserved domain with a winged helix-turn-helix (wHTH) fold that is predicted to bind double-stranded RNA. We show that this domain is widely conserved in eukaryotes and bacteria and appears to be a novel RNA-binding domain that might have key role in the assembly and localization of RNA-protein complexes with important post-transcriptional regulatory functions.

## Identification of the OST-HTH domain

**Figure 1:**
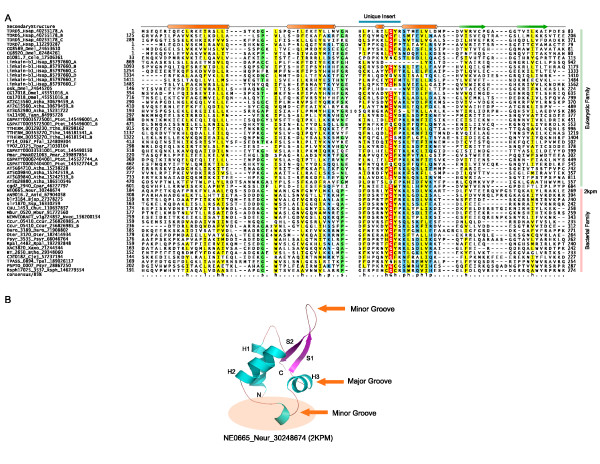
**A. Multiple alignment of selected representatives of the OST-HTH superfamily**. The consensus shown below was computed on a complete alignment of the superfamily which included 256 non-redundant versions of domain and was used to color columns of the alignment. The consensus positions are labeled thus: h- hydrophobic, l- aliphatic, s-small and p-polar. The proteins are labeled with the protein name followed by the organism abbreviation and Genbank Gi number delimited by underscores. The organism abbreviations are: *Anid: Aspergillus nidulans; Atha: Arabidopsis thaliana; Bjap: Bradyrhizobium japonicum; Bthe: Bacteroides thetaiotaomicron; Ccur: Cryptobacterium curtum; Cele: Caenorhabditis elegans; Chut: Cytophaga hutchinsonii; Cjej: Campylobacter jejuni; Cpar: Cryptosporidium parvum; Daro: Dechloromonas aromatica; Dmel: Drosophila melanogaster; Hsap: Homo sapiens; Mbur: Methanococcoides burtonii; Neur: nitrosomonas europaea; Nvec: Nematostella vectensis; Oter: Opitutus terrae; Pfal: Plasmodium falciparum; Pmar: Perkinsus marinus; Psyr: Pseudomonas syringae; Ptet: Paramecium tetraurelia; Rpal: Rhodopseudomonas palustris; Rsph: Rhodobacter sphaeroides; Ssp: Synechocystis sp.; Tann: Theileria annulata; Tpal: Treponema pallidum; Tpar: Theileria parva; Tthe: Tetrahymena thermophila; Xaxo: Xanthomonas axonopodis; Osat: Oryza sativa; Vvin: Vitis vinifera; Jant: Jonquetella anthropi; Pinf: Phytophthora infestans*. **B**. The average NMR structure of the OST-HTH domain from the protein NE0665 (2 kpm) is rendered as a cartoon with the helices and strands labeled as per the secondary structure progression. The regions inferred to potentially contact dsRNA based on the superposition to the archaeal CDC6-DNA co-crystal structure are indicated. The region of the OST-HTH that is circled is the unique insert that distinguishes it from other wHTH domains.

Analysis of the *Drosophila *polar granule protein Oskar showed that it contains a C-terminal catalytically inactive SGNH hydrolase domain [9] and an uncharacterized N-terminal predicted globular region. To better understand the affinities of this N-terminal globular region (gi: 24645205, residues 8-112) we used it as a seed for iterative sequence profile searches with the PSI-BLAST program (profile inclusion threshold = .01; iterated to convergence) of the Non-Redundant database (NR). In addition to recovering close orthologs in insects, the search also recovered a comparable region from the mammalian nuage complex proteins TDRD5 (gi: 134035042, e = 0.001) and TDRD7 (gi: 112293287, e = 0.004) [10] within three iterations. Given that this relationship is consistent with a functional connection between these proteins, we initiated further transitive PSI-BLAST searches with the corresponding region in TDRD5 and TDRD7. The search with the region from TDRD7 (residues: 5-96) recovered further proteins such as the *Drosophila *protein CG8920 (gi: 62484261, e = 2 × 10^-15^; iteration 3), vertebrate protein limkain b1 (gi: 85797660, e = 10^-4^, iteration 5), the *Arabidopsis *protein AT2G15560 (gi 30679459, e = 5 × 10^-7^, iteration 6), proteins typified by TTHERM_00129230 from ciliates (gi: 89298162; e = 0.006, iteration 7) and cgd2_2940 and its orthologs from apicomplexans (gi:46227797; e = 0.006, iteration 11). Some of these proteins, such as TDRD5, TDRD7 and Limkain b1 from vertebrates showed multiple tandem repeats of the homologous region that enabled us to precisely determine its boundaries (Fig. 1). The search also recovered several similar regions in bacterial proteins with marginal significance (e.g. gi: 239623453 and 256826985, e = 0.03-.05). A parallel search with the JACKHMMER program using the above query also identified these bacterial proteins with e-values < .01. Furthermore, both PSI-BLAST and JACKHMMER searches seeded with bacterial starting points (e.g. gi: 256826985) recovered several of the eukaryotic proteins with e-values < .001 within 12 iterations. The relationship between the bacterial and the eukaryotic versions was also confirmed (p < 10^-5^) in a profile-profile comparison using the HHSEARCH program. PSI-BLAST and JACKHMMER searches recovered a bacterial representative NE0665 (gi: 30248674) from *Nitrosomonas europaea*, whose NMR structure has been determined as part of the structural genomics initiative (2 kpm; Fig. 1).

Examination of this structure showed that the domain adopts a winged helix-turn-helix (wHTH) fold, which is characterized by a core 3-stranded HTH with a C-terminal extension of two strands. Structure prediction of just the eukaryotic versions using the JPRED program, which combines information from residue frequencies in columns, a HMM and a PSSM derived from the alignment, also predicted a secondary structure progression completely congruent with that observed in the above bacterial version. Based on these observations we named this novel conserved domain the *Os*kar-*T*DRD5/TDRD7 HTH (OST-HTH) domain.

## Phyletic patterns and domain architectures of OST-HTH superfamily

**Figure 2:**
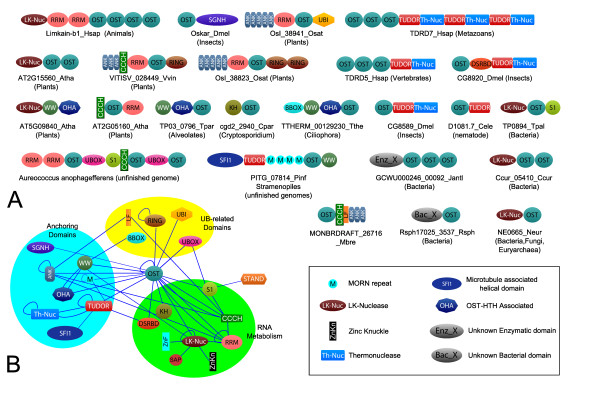
**A. Domain architectures of OST-HTH proteins**. The domains are shown approximately scale but the intervening non-globular regions are not shown. The phyletic pattern typical of each architecture is shown below the illustrated representative. The species abbreviations and protein labels are as in figure 1. We observed that the DUF2384 domain fused to certain bacterial LK-nucleases is another HTH domain distinct from the OST-HTH described in this article. A key is provided for the domains whose names have been abbreviated in a non-standard fashion. **B. Architectural network for the OSK-HTH domain**. The network is centered on the OST-HTH with the other domains grouped as per their function. The arrows indicating the directionality of the connections have been omitted for easier examination.

The OST-HTH superfamily is primarily found in bacteria and eukaryotes with very rare occurrences in archaea (*e.g. *Mbur_0520 *Methanococcoides burtonii*) that appear to be relatively late lateral transfers of bacterial versions. The bacterial and the eukaryotic versions can be distinguished based on their sequence conservation patterns (Fig. 1) and constitute two distinct families of the OST-HTH. The bacterial family is found sporadically but widely across the bacterial tree with examples from practically all major bacterial lineages (Additional File 1). In eukaryotes, representatives are found in metazoans, choanoflagellates, plants, ciliates, apicomplexans and stramenopiles. Almost all of the bacterial versions show stereotypic domain architectures with a single or duplicated OST-HTH fused to an N-terminal globular domain annotated as a domain of unknown function (DUF88) in the PFAM database (Fig. 2). Fusions of the OST-HTH to the latter domain were also observed in several eukaryotic proteins typified by the human Limkain b1 protein [11] that contain additional RNA-binding RRM domains. Likewise, some bacterial proteins contain further fusions to the S1/Cold-shock-type OB fold RNA-binding domain (Fig. 1; Additional file 1). Sequence profile analysis and structural comparisons of the crystal structure of a representative of these "DUF88" domains from *Vibrio parahaemolyticus *(VPA0982; PDB: 2qip) showed that it is a member of the 5'->3' nuclease domain superfamily that includes RNAses such as the PIN, NYN, and phage T4-type viral RNAse H domains [12]. Hence, we term this domain the LK-nuclease domain after its presence in Limkain b1. In addition to the OST-HTH domain, the LK-nuclease domain is also fused to RNA-binding Zn-knuckle domains in certain proteins (Fig. 2).

Eukaryotic members of the OST-HTH superfamily, other than those in Limkain b1, show a remarkable diversity of architectures (Fig. 2). Versions from different eukaryotic lineages show fusions to multiple, distinct single- and double- stranded RNA-binding domains such as the CCCH, RRM, KH, S1/Cold-shock-type OB fold and the dsRBD domains [13]. In addition to these RNA-binding domains the OST-HTH is also linked to several protein-protein interaction domains in various eukaryotes. In metazoans and stramenopiles the OST-HTH shows multiple fusions to the methylated peptide-recognizing Tudor domains [6,7]: *e.g. *in vertebrate TDRD5/TDRD7 and arthropod proteins typified by CG8920, one or more copies of the Tudor domain occur with one or more copies of the OST-HTH (Fig. 2). In other eukaryotes the OST-HTH shows fusions to distinct interaction domains, such as ankyrin repeats in plants and independent fusions to WW domains in apicomplexans, ciliates and stramenopiles. Related WW domains are also fused to the LK-nuclease domain in plants (Fig. 2). The arthropod Oskar proteins are unique in being fused to a C-terminal SGNH hydrolase domain. SGNH hydrolases are esterases of fatty acid esters (*e.g. *lipases) [14]; however, examination of this domain reveals that two of the key residues of the hydrolase catalytic triad (a serine and a histidine) are disrupted (Additional file 1). Hence, this domain in Oskar is likely to be an enzymatically inactive lipid-binding version. Stramenopiles show another potential lipid-binding module, namely MORN repeats. In ciliates, plants, stramenopiles and choanoflagellates, in addition to RNA-binding domains, the OST-HTH is also linked diverse domains specific to the ubiquitin conjugation network (Fig. 2). These include the ubiquitin E3 ligase domains with the treble clef fold, such as the B-Box, U-box and different types of RING fingers, the "Little finger", which is a ubiquitin-binding Zn-ribbon, and ubiquitin-like domains that could also bind Ub [15,16].

We represented the domain architectures of the OST-HTH as an ordered network where each node is a domain and each edge represents co-occurrence of two domains as immediate neighbors in the same polypeptide (Fig. 2). This network reveals three major themes: 1) linkages to multiple RNA-binding domains and a potential RNAse, namely LK-nuclease domain. Given that representatives of the wHTH fold have been previously implicated in binding nucleic acids [17], the OST-HTH is likely to possess RNA-binding activity by itself or in conjunction with other domains. 2) In eukaryotes there are multiple fusions of the OST-HTH to domains that could play a role in anchoring these proteins to the cytoskeletal proteins (e.g. Ankyrin repeats, Sfi1 and the WW domains), to modified proteins in RNPs (Tudor domains) or to lipid membranes (inactive SGNH hydrolase domain and MORN repeats). These linkages implicate eukaryotic OST-HTH proteins in functions that might be specifically related to localization of RNA to particular cellular sub-structures. 3) In several eukaryotic lineages the OST-HTH has been independently combined with various domains specific to the Ub-system. Such proteins could potentially modify other proteins bound to RNA, or associate with ubiquitinated proteins by means of their Ub-binding domains.

## Structural analysis of the OST-HTH domains suggests potential dsRNA-binding capability

Others and we have formerly characterized several other RNA-binding domains with the classical and circularly permuted versions of the wHTH fold [17]. Examples of the former version are the ribosomal protein S19AE, RNA 2'phosphotransferase, La, the ribosomal protein S10E, a domain of the archaeal-type phenylalanyl tRNA synthetase α-subunit and the RNA-binding modules of the selenocysteine-specific translation factor SelB. The latter variety of the wHTH fold is represented by the translation factor IF2 N-terminal domain and the phenylalanyl tRNA synthetase β-subunit. However, the OST-HTH revealed no specific relationship to any of those domains beyond sharing a common fold (Fig. 1). This suggested that it is probably an independent adaptation of the wHTH fold for a RNA-binding function. To better understand the mode by which the OST-HTH might contact RNA, we compared it with other known structures by performing a search of the PDB database with the DALI program using its representative structure (2 kpm; *Nitrosomonas europaea *NE0665) as a query. The best hit in this search was the wHTH domain of the archaeal replication protein CDC6 that binds dsDNA [18]. Both these wHTHs share a distinct extended insert between the helix-2 and helix-3 of the wHTH domain. However, the OST-HTH is unique among all other wHTHs with characterized structures because this insert region assumes a distinctive conformation with at least two turns of a helix (Fig. 1). On the basis of the superposition of the OST-HTH structure on the CDC6 wHTH-dsDNA co-crystal structure we inferred that OST-HTHs could potentially make comparable contacts with dsRNA with the tip of the 'wing' contacting the minor groove. In addition to the possible major groove contacts by helix-3, this superposition suggests that residues located in the unique insert between helix2 and helix-3 of the OST-HTH structure could also contact a second minor groove, distinct from the one contacted by the wing (Fig. 1). Indeed, this insert region has one of the defining motifs of the OST-HTH domain, namely a conserved Ghxph motif (where p is polar, h is hydrophobic and x is any residue; Fig. 1) suggesting that contact via this region might be a prevalent feature in the superfamily. These observations, together with the circumstantial evidence from the domain architectures reported above and the functional characterization of proteins like Oskar and TDRD5/TDRD7 [1,10], suggest that the OST-HTH might specialize in contacting dsRNA or stems of folded structures in RNA.

## General conclusions and evolutionary implications

The prediction of a RNA-binding domain with potential dsRNA-binding properties is of considerable significance in understanding the organization of the nuage-like RNP complexes. Firstly, the OST-HTH is a common denominator of the Oskar proteins of arthropod polar granules and TDRD5/TDRD7 vertebrate nuage complexes. This, taken together with our functional prediction, suggests that there could be a common principle in the nucleation of these complexes that proceeds via recognition of dsRNA. The nuage complex has been shown to contain miRNAs, rasiRNAs and piRNAs that form dsRNA with their targets. These dsRNA molecules could be potential targets bound by the OST-HTH. Plants possess both regulatory miRNAs and rasiRNAs that target transcripts from repetitive elements. Ciliates have a regulatory system of small RNAs similar to piRNAs, namely the scnRNAs that are critical for elimination of transposons and other DNA elements from the somatic macronucleus [19]. While little is known of the post-transcriptional gene regulation in stramenopiles, key components of the RNAi machinery are detectable in several stramenopile lineages. These include peculiar RNA-dependent-RNA polymerases with N-terminal PHD fingers (e.g. THAPSDRAFT_9018, gi: 224008402 from the diatom *Thalassiosira*). These findings indicate the presence of robust RNAi machinery that might be specifically involved in chromatin level silencing as in other eukaryotes. Identification of several OST-HTH superfamily members in these eukaryotic lineages raises the possibility that they participate in the formation of RNP structures that are critical for counter-transposon defense. As in the case of the animal nuage/germinal granules these RNPs could also participate in sub-cellular localization of various RNAs in various eukaryotes. For example, in photosynthetic stramenopiles such as *Aureococcus anophagefferens *there are giant proteins in which the OST-HTH domain occurs with multiple distinct RNA-binding and ubiquitin E3 ligase domains in the same polypeptide and could function as large scaffolds for assembly of RNP complexes (Fig. 2). In animals neuronal RNPs have been implicated in phenomena such as learning and memory via specific intra-neuronal RNA localization - it would be of interest to investigate if such localization might require OST-HTH proteins. Detection of members of the OST-HTH superfamily in apicomplexans such as *Plasmodium *and *Cryptosporidium *(Fig. 1 and 2), which apparently lack the conventional RNAi machinery, suggests that these OST-HTH proteins could function independently of the small RNAs of the RNAi system. In these organisms they could bind dsRNA generated by other mechanisms (e.g. anti-sense transcription) or even folded structures in mRNA. In any case, the OST-HTH superfamily members in these organisms point to potential RNA localization and post-transcription regulatory mechanisms that have not been previously characterized.

Combination of the OST-HTH with several domains of the Ub-system suggests a previously under-appreciated role for ubiquitination and interactions with ubiquitin in the assembly and stability of nuage-like RNPs across eukaryotes. Previously the ubiquitin E2-ligase UBC3B/UBE2R2 has been shown to be a component of the mammalian nuage complexes, but is role was unclear [3]. In light of our findings it is likely that it is part of the Ub-dependent system that regulates nuage assembly. The fusion to the LK-nuclease domain suggests that the OST-HTH might also recruit substrates for processing or degradation. In this capacity they could function as part of or in parallel to the RNAi-mediated degradation events that help in elimination of deleterious transcripts, such as those from transposons [3,5,19]. However, genes for the bacterial versions (i.e. LK-nuclease+the OST-HTH proteins; Fig. 2) show no linkages in the form of conserved gene neighborhoods to genes of any other known RNA-processing systems like the CRISPR system or the RNA-dependent RNA polymerase [20]. Hence, in bacteria they might function as a novel, standalone RNA-degradation enzyme.

Examination of the phyletic patterns (additional file 1) suggests that the OST-HTH most probably emerged in the bacteria, where it was widely dispersed through lateral transfer. There was probably one ancient transfer to the eukaryotic lineage. Its absence in basal eukaryotes such as diplomonads and parabasalids suggests that this transfer might have occurred after the divergence of these lineages. The presence of a conserved version across eukaryotes with the LK-nuclease domain (Fig. 2, additional file 1) suggests that this was probably the version that first entered eukaryotes and might have retained a role comparable to its bacterial cognates in RNA degradation. However, in eukaryotes the OST-HTH developed a distinct role of its own via combinations to multiple domains involved in protein-protein interactions or ubiquitination. Thus it appears to have become a major player in RNA-localization, RNP-nucleation/assembly and RNA-associated protein modifications.

## Materials and methods

Sequence profile searches were performed against the NCBI non-redundant (NR) database of protein sequences (National Center for Biotechnology Information, NIH, Bethesda, MD), and a locally compiled database of proteins from eukaryotes with completely or near-completely sequenced genomes. PSI-BLAST searches [21] were performed using an expectation value (E-value) of 0.01 as the threshold for inclusion in the position-specific scoring matrix generated by the program; searches were iterated until convergence. Profile-based HMM searches [22] were performed using the newly released HMMER3 package (version beta 2). Pairwise comparisons of HMMs, against profiles were performed with the HHPRED program [23]. Multiple alignments were constructed using the Kalign programs, followed by manual correction based on PSI-BLAST high-scoring pairs, secondary structure predictions, and information derived from existing structures [24]. Protein secondary structure was predicted using a multiple alignment as the input for the JPRED2 program. Structure similarity searches and structural alignments were performed using the DALI program [25]. Protein structures were visualized and manipulated using the Swiss-PDB and PyMol programs http://pymol.sourceforge.net/. Similarity-based clustering was performed using the BLASTCLUST program ftp://ftp.ncbi.nih.gov/blast/documents/blastclust.html with empirically determined length and score threshold parameters. All large-scale procedures were carried out using the TASS software package (Anantharaman V, Balaji S, Aravind L, unpublished results).

## Competing interests

The authors declare that they have no competing interests.

## Authors' contributions

VA and LA were made the discovery and wrote the paper. DZ was performed the analysis on the LK-nuclease (DUF88) domain. All authors read and approved the final manuscript.

## Reviewers' comments

### Reviewer 1

Sandor Pongor, International Centre for Genetic Engineering and Biotechnology, Trieste, Italy

The discovery of RNA regulatory mechanisms is one of the most fascinating insights of modern biology. RNP granules are especially difficult targets within this widespread and varied set of molecular scenarios because of their complex and presumably loosely defined molecular architecture is not easily amenable to structural studies. The mechanism by which RNP granules self-organize at specific cellular locations is currently poorly understood. The authors present a predictive bioinformatics analysis of the proteins of the polar granule and nuage complexes, based on sequence alignment, domain architecture analysis and sequence-structure superpositions and suggest that a hitherto unknown domain-type, OST-HTH, with a potential specificity for dsRNA may play a key role in the recruitment process that anchores nucleoprotein granules including miRNAs, rasiRNAs and piRNAs to specific cellular structures. The paper is clearly and carefully presented and the conclusion represents an important advance.

1. The Materials and Methods section could be more detailed and more understandable for a wide audience. Minor: I did not find reference to JACKHMMER,

**Response**: A detailed Materials and Methods in available as a part of Additional File 1

### Reviewer 2

Arcady Mushegian, Stowers Institute, Kansas City, United States

I have no concerns about the validity of the sequence relationships and structural predictions described in this work. Several queries to the authors:

"It is also possible that the nuage complex is related to other major RNP granules such as those found in neurons [3]." -- related in which way? Two complexes may well include some homologous protein components - is this "related" in an interesting sense, or is something more dramatic implied?

**Response**: The two RNPs indeed share several common proteins or those with homologous RNA-binding domains and similar architectures. Additionally, both neuronal RNPs and nuage-like complexes share other distinctive features: 1) A similar ultrastructure; 2) Presence of dsRNA; 3) intracellular motility. The motility of both these granules marks them as specialized elements required for active sub-cellular RNA localization as against passive aggregations of RNAs complexed with proteins.

p.6 last paragraph: I do not think that the network shown in Fig. 2 reveals any of the observations that the authors say it reveals. In fact, each of these three observations have been described by the authors earlier in the paper without resorting to network representation. Thus, Fig. 2 is another way to illustrate the points already made. Network structure can be used to quantify the relationships and to find new connections that are not easily revealed by other methods, but this is not such a case.

This point is in fact not so minor, as it concerns the explanatory and predictive utility of networks in biology.

**Response**: The network in Fig. 2B is indeed a representation of the same information as the domain architectures illustrated in the panel A. However, it is not useless because it helps a reader to easily perceive the main themes in the linkages to other domains as they are grouped together and categorized according to their general function in Fig. 2B. Hence, we are of the opinion that this network does serve as an explanatory aid. There is quantitative information in the network from which the figure is derived that further supports the basic points being made in the text: In 70% of the distinct architectures, the OST-HTH has an RNA-binding domain as an immediate neighbor or a neighbor-one removed (separated by one intervening node). In 58% of the distinct architectures it shows such neighborhood relationships to protein- or lipid- interacting domains with "anchoring" functions. In 25% of the architectures it shows such neighborhood linkages to the Ub-system related domains. The first value is comparable to major RNA-binding domains like RRM, S1-like OB fold and Zn-knuckles and the other two values are significantly higher (p < .001) than the equivalent linkages to domains belonging to the same functional categories in a sample network of eukaryotic RNA-binding domains (derived from 1530 proteins presented in PMID: 14519199)

## Supplementary Material

Additional file 1 This file contains additional material and methods, data for phyletic patterns, domain architectures and alignments of OST-HTH and associated conserved domains. Click here for file
