# Supplementary material for: OST-HTH: a novel predicted RNA-binding domain
Source: Biol Direct. 2010 Mar 19;5:13. doi: 10.1186/1745-6150-5-13 (PMC2848206; doi:10.1186/1745-6150-5-13)
Supplement: Additional file 1 — This file contains additional material and methods, data for phyletic patterns, domain architectures and alignments of OST-HTH and associated conserved domains. [file 1745-6150-5-13-S1.HTML]

OST-HTH: A novel predicted RNA-binding domain


# 

**OST-HTH:
A novel predicted RNA-binding domain**.

Vivek Anantharaman, **Dapeng
Zhang*,***and
 L.
Aravind\*  
  
\* *Address
for
correspondence: L. Aravind (aravind@mail.nih.gov)*  
  
*National
Center for Biotechnology
Information, National Library of Medicine, National Institutes of
Health, Bethesda, MD 20894, USA*


---

  

Abstract  

The
mechanism by which the arthropod Oskar and vertebrate TDRD5/TDRD7
proteins nucleate
or organize structurally related ribonucleoprotein (RNP) complexes, the
polar
granule and nuage, is poorly understood. Using sequence profile
searches we
identify a novel domain in these proteins that is widely conserved
across
eukaryotes and bacteria. Using contextual information from domain
architectures, sequence-structure superpositions and available
functional
information we predict that this domain is likely to adopt the winged
helix-turn-helix
fold and bind RNA with a potential specificity for dsRNA. We show that
in
eukaryotes this domain is often combined in the same polypeptide with
protein-protein- or lipid- interaction domains that might play a role
in
anchoring these proteins to specific cytoskeletal structures. Thus,
proteins with
this domain might have a key role in the recognition and localization
of dsRNA,
including miRNAs, rasiRNAs and piRNAs hybridized to their targets. In
other
cases, this domain is fused to ubiquitin-binding, E3 ligase and
ubiquitin-like
domains indicating a previously under-appreciated role for
ubiquitination in
regulating the assembly and stability of nuage-like RNP complexes. Both
bacteria and eukaryotes encode a conserved family of proteins that
combines
this predicted RNA-binding domain with a previously uncharacterized
domain
(DUF88). We present evidence that it is a RNAse belonging to the
superfamily that
includes the 5’->3’ nucleases, PIN and NYN
domains and
might be recruited to
degrade certain RNAs

  


---

Contents  
  

- Materials And Methods
- Multiple
  Alignment of OST-HTH
- Multiple
  Alignment of LK-Nuclease(DUF88)
- Multiple
  Alignment of the divergent WW family found in Apicomplexa
- Multiple
  Alignment of OHA (OST-HTH associated domain)
- Multiple
  Alignment of the inactive SGNH-Hydrolase domain of Oskar
- Multiple
  Alignment of the bacterial domain X
- Multiple
  Alignment of the enzymatic bacterial domain
- Family
  clusters of OST-HTH domain containing proteins with domain
  architecture

  


---

Materials
And Methods  
  

The
non-redundant (NR) database of protein sequences (National
Center
for Biotechnology
Information,
NIH, Bethesda)
was searched using the BLASTP program [1].
Iterative
database searches were conducted using the PSI-BLAST program with
either a
single sequence or an alignment used as the query, with the PSSM
inclusion
expectation (E) value threshold of 0.01 (unless specified otherwise);
the
searches were iterated until convergence [1,
2].
For all searches with
compositionally biased proteins, the statistical correction for this
bias was
employed. Multiple alignments were constructed using the KALIGN
programs [3,
4],
followed by manual correction based
on the PSI-BLAST results. Globular domains were predicted using the SEG
program
with the following parameters: window size 40, trigger complexity=3.4;
extension complexity=3.75 [5].
All large-scale
sequence analysis procedures were carried out using the TASS package
developed
by our group (unpublished; Vivek Anantharaman, Santhanam Balaji, and L.
Aravind). Protein secondary structure was predicted using a
multiple
alignment as the input for the JPRED program [6].
Similarity-based
clustering of proteins was carried out using the BLASTCLUST program
(For
documentation see ftp://ftp.ncbi.nih.gov/blast/documents/README.bcl).
Profile-based HMM searches [7]
and JACKHMMER 
were performed using the newly released HMMER3 package (version beta 2)
(http://hmmer.janelia.org/). 
Pairwise comparisons of HMMs, against profiles were performed with the
HHPRED program [8] (http://toolkit.tuebingen.mpg.de/hhpred).
 

 References

1.           
Altschul
SF, Madden TL, Schaffer AA, Zhang J, Zhang Z, Miller W, Lipman DJ:
Gapped BLAST and PSI-BLAST: a new
generation of protein database search programs. *Nucleic
Acids Res* 1997,
25(17):3389-3402.

2.           
Aravind L, Koonin EV:
Gleaning non-trivial structural,
functional
and evolutionary information about proteins by iterative database
searches.
*J Mol Biol* 1999,
287(5):1023-1040.

3.           
Lassmann T, Sonnhammer EL:
Kalign--an accurate and fast multiple
sequence alignment algorithm. *BMC
Bioinformatics* 2005, 6:298.

4.           
Lassmann T, Sonnhammer EL:
Kalign, Kalignvu and Mumsa: web
servers for
multiple sequence alignment. *Nucleic
Acids Res* 2006, 34(Web Server
issue):W596-599.

5.           
Wootton JC: Non-globular
domains in protein sequences: automated
segmentation using
complexity measures. *Comput
Chem* 1994,
18(3):269-285.

6.           
Cuff JA, Barton GJ:
Application of multiple sequence alignment
profiles to improve protein secondary structure prediction. *Proteins*2000, 40(3):502-511.

7.
           Eddy SR: Profile hidden Markov
models. Bioinformatics 1998,
14:755-763.

8.
           Soding J: Protein homology
detection by HMM-HMM comparison. Bioinformatics
2005, 21:951-960.

 

   
  
  
  
  
Top  


---

  
  
Multiple
Alignment of OST-HTH   

|  |
| --- |
| FINAL                                  ------HHHHHHHHHHHHHHH--------------HH--HHHHHHHHH-----------------HH----HHHHHHHH-------EEEEEE---------EEEEEEE---  osk\_Dmel\_24645205                      YISVREEYPDIDSEVRAILLS--HAQNG-----ITIS--SIKSEYRKLTGN----------PFPLHD----NVTDFLLTIP----NVTAECSE------SGKRIFNLKASL  Caps1000022651\_Caps\_Caps1000022651     --MSKTSRESVTKEVRSILL---ASQRG-----MSCR--EFVREWNELLGY----------PIPHRQLGFASLEDFLRGLP----EVVELSWQ------DGELILKAVANE  Caps1000022651\_Caps\_Caps1000022651\_2   VTQRRTVNPFLRGQILQLLN---AFPHG-----LLGS--NFLLAFSRKFGR----------NIAPSALGFQSVNEFLQSMP----DILRFENLSS----GGYKVFPLNGQQ  Caps1000022651\_Caps\_Caps1000022651\_3   MDDHSGLAGDIKANIRKVLS---RFPAG-----MRCL--RFTQQYKSMIGQ----------ELDYQQYGFYNLVGLMSTIP----DVVKVEKISA----NDWLLSDARIKG  Caps1000022651\_Caps\_Caps1000022651\_4   LQTAPPIPEEIQEKIRQVVL---QFPNG-----LPLS--NLQQNFEDLVGE----------ALNYVTLGYKSIDAMIRQME----TKVTMR--------NYVLFPANDSDP  TDRD5\_Hsap\_40255178                    MSEQERIQECLRKEIRSLLI---STKDG-----LSPQ--ELEKEYLLMVGN----------HLPLRILGYRSTMELVLDMP----DVVRVCPGA-----GGTVILKAIPDE  TDRD5\_Hsap\_40255178\_2                  GRVAPILPAVVKSELKDLLA---L--SP-----VLLS--DFEKAFAKRFGR----------SFQYMQYGFLSMFEVLNAAS----DVISVEQTRA----GSLLMLKKSVTE  TDRD5\_Hsap\_40255178\_3                  IGPGGTISSELKHKIKFVVS---KFPEG-----LFIS--KLLGEYEVIFKE----------QLSPKKLGFLNVTELVGALS----DILHVEFRK-----GHQDLLVFDADK  LOC100177761\_Cint\_198418545            MSELLAEKNKVKKVLRSLLL---SASCG-----LTPT--QLERDYKQMAGE----------VLPYKKFHFMTSRDFLKSLR----DTLVFVYD------GPHVIIHGIADD  LOC100177761\_Cint\_198418545\_2          KAPTLYVPAFIRNNLFKVIK---TFPNG-----FSIS--ILPEIYSKVNGA----------KLHINGHGFENHRDLLLAMP----DIVRLEF-------GRTNRAGQNLYV  LOC100177761\_Cint\_198418545\_3          KQEYSPACQILQRELLQLLS---GRPNG-----TWSL--NISSLYEQKYNK----------PLELKNYGYFSIIEFMSTLG----NKVSMRPLPY----SGDWFFNIRSPN  TDRD7\_Hsap\_112293287                   ----MLEGDLVSKMLRAVLQ---SHKNG-----VALP--RLQGEYRSLTGD----------WIPFKQLGFPTLEAYLRSVP----AVVRIETSR-----SGEITCYAMACT  BRAFLDRAFT\_64001\_Bflo\_210131123        MSSKAKMVQEVKKDIRSLLI---SAQRG-----LTAT--MLLDDYKQFIGH----------PLPYRELGYNAPIDFIRDIS----DVVQLTWQ------GSMQVLRAVVDE  BRAFLDRAFT\_64001\_Bflo\_210131123\_2      PAPTASVPAGVRTQVKQLLA---SYPSG-----IPSS--AFNTAFGRRFGS----------QINYTKLGYKSMGELLRALR----DIILIRPQA-----GSTFRIYGRGDS  BRAFLDRAFT\_64001\_Bflo\_210131123\_3      PASCGVIDPVLKAEITEVLA---ERPNG-----MWAK--RLPFEYKKKLGK----------ELPLQENGFYSVIEMISALT----DVAVITRPNP----NGDWWLCEQKAY  BRAFLDRAFT\_64001\_Bflo\_210131123\_4      VIAPVASPPSLEMLIVQVLL---KHPEG-----IKSK--QFPEVFKTQTGR----------TLPVQERGYRSTETLLLDME----DYVRMVYKG-----AGTVMLYAREGA  Dpul1000007973\_Dpul\_Dpul1000007973     VSPKDVVLETLKEDIHALLC---AVKGS-----VPSE--QIIKDYRMVCGS----------HLPYRQLGFASVNDLLKSIP----DVVSCHNL------NGNVLVKAVLKN  CG8589\_Dmel\_24653610                   -MDDGGELSLVKKVVHSLVV---SSPGK-----LTVE--QLMRDYRSEEGC----------TLPYSKLGFKDAESFLRSIP----DTVTVTGH------GQMAWITAVATA  Lgig1000022804\_Lgig\_Lgig1000022804     MSSVAVIKEEAEKQIRALLN---STPAG-----LAVG--ELKRDYQQFIGS----------QIPYQKFGYSSAEDYFNDIP----DVITPVWE------NGKTILKATANE  Lgig1000022804\_Lgig\_Lgig1000022804\_2   YVTTPQVPVSIRHQIKQMFR---Q-NRS-----LPLT--HFDNLFFKTFGH----------HLDHERFGYSSVEYLLKSIP----DTVTLINQH-----GEWRVLSAYARY  Lgig1000022804\_Lgig\_Lgig1000022804\_3   SPEPEEFEDPVHKELKKVLS---SHSKG-----IMAS--RLAFEYKAIIGK----------ELPFKDHGYHSVIEFVNDIP----NVVRIERPNP----SGDFILFDATAP  LOC100204243\_Hmag\_221115123            ---MKENLEEVKKIVRSLLL---SAKQG-----LTIN--QLCSEYTDLEHK----------TLPYKEFGYNSAISFMKSMP----DVVRPVFLS-----RGDMVLQGVADE  NEMVEDRAFT\_v1g205481\_Nvec\_156221401    KAEREKTKQQVSRHLRALLI---SAPNG-----LNVV--EVQNDYFKFIGK----------HLPYRELGYKSALELLQALP----DVVRPSWV------RGELILRGIVDE  NEMVEDRAFT\_v1g205481\_Nvec\_156221401\_2  PSLPPNVPYFTRSKIKELLS---FYPSG-----LLGA--MFPQAYERHCKH----------ALDVKGIGFGSSQELLRSIP----DIARIEQIK-----GGGYRVYAVQEK  NEMVEDRAFT\_v1g205481\_Nvec\_156221401\_3  IDSQNSVSRMLREEIAKILN---MRPNG-----VWSA--RLPVEYKKLFKK----------ELVVRQHGFTSVIELADAIP----DIVRIHRPTK----TGDWLLLGNKKG  LOC100183257\_Cint\_198415060            ----MSDLENVKKNLRSVLL---SSKQG-----IVAK--HLQREYRELNGE----------FIPHQKLSFCSLEEFIRSLS----DVCRVGY-------NNQNELTYFALT  LOC726241\_Amel\_110764792               -----MEREEVIKNLRACLI---SSKGG-----VKME--DLNRDYKMLIDE----------NIPFRKLGYQSLVAFLRTVQ----SVRIIEK-------AGECFVEAIPSE  CG8920\_Dmel\_62484261                   -MEKQEVLEYVAKVVRALIT---SAKPP-----VTLR--SIVADYMEIEGE----------PIPFRRLGYSNDQELLKDSN----QFNFHQS-------GNQVFITAKYNA  Dpul1000021766\_Dpul\_Dpul1000021766     MTDKEKLRNEVKLEIQALLC---TEKSA-----ITSK--RLYGLYISEMGT----------VMPYAKLGYNQMDDFLRSIP----DVVSSYTNFQ----DGMLYVKAVPLE  NEMVEDRAFT\_v1g175950\_Nvec\_156209296    ---MEEMKEKTERMLRAVLI---SAPRG-----IPIY--KLDKEYKSITFE----------PIPFKKCGYPSLEAFIRASP----KVATIQTDR-----DGEIIVKGVASE  Caps1000018855\_Caps\_Caps1000018855     ---MTRVPDDLKVEIRSVLL---STIGG-----IKTN--FFLKEFKDLTQY----------ELNYRKYGFSSLSEFLASMP----DVCRLDFNKQ----GQPAVFGILTNE  Caps1000009677\_Caps\_Caps1000009677     MGSQEEELKKLKVNIRAVLE---SINGG-----VELT--RFNWEYQEVTMEP---------NINFKKFNCNSLVEFFQKFCD---STVSIRRNNE----GSEMCMPVFSEK  D1081.7\_Cele\_17506361                  ENQKVDPMRKVKIELQAVLV---AEKIP-----ISTE--EIRRRLLDSYGA----------CPDPKRYNCSTLDDLLQACS----ESIVHTFGR-----DGIHRYGPRTTE  LOC100207520\_Hmag\_221129949            NDSMSIIDDQVKQLLRSVLV---S-APG-----VKQS--SLEHEYYKITNE----------VLPWRKYKFPNLFEFLKALE----GEITRIEF------SEKFADNIIYAV  KIAA0430\_Hsap\_85797660                 TGAASKSLSLLSAETMSVLQ---DAPACC----LPLF--KFTDIYEKKFGH----------KLNVSD--------LYKLTD-----TVAIREQ------GNGRLVCLLPSS  KIAA0430\_Hsap\_85797660\_2               SPVGNPQLIQFSREVIDLLK---SQPSCV----IPIS--HFIPSYHHHFAK----------QCRVSDYGYSKLIELLEAVP----HVLQILGM------GSKRLLTLTHRA  KIAA0430\_Hsap\_85797660\_3               -QDEIERTKQFSKDVVDLLR---HQPHFR----MPFN--KFIPSYHHHFGR----------QCKLAYYGFTKLLELFEAIP----DTLQVLEC------GEEKILTLTEVE  KIAA0430\_Hsap\_85797660\_4               -----ERFKALAAQFVKLLR---SQKDNC----LMMT--DLLTEYAKTFGY----------TFRLQDYDVSSISALTQKLC----HVVKVADIES----GRQIQLINRKSL  KIAA0430\_Hsap\_85797660\_5               -----KSLRSLTAQLLVLLMS--WEGTTH----LSVE--ELKRHYESTHNT----------PLNPCEYGFMTLTELLKSLP----YLVEVFT-------NDK--MEECVKL  KIAA0430\_Hsap\_85797660\_6               ECVKLTSLYLFAKNVRSLLH---TYHYQQ----IFLH--EFSMAYTKYVGE----------TLQPKTYGHSSVEELLGAIP----QVVWIKGH------GHKRIVVLKNDM  Caps1000025556\_Caps\_Caps1000025556     PSLGQ-QLTQFSSELIDLLK---SSPQCR----MPFS--KFIPSYHHHFGK----------QCRVADYGYTRLMELFDSMP----HTVQVLGS------GNRRYITLAHRT  Caps1000025556\_Caps\_Caps1000025556\_2   TLAHRTQIKRFATDLLRVLR---TQSSKQ----VSIL--MFPSVYQKVFSK----------SFDIYEYGVCYLEDLLCELED---NTIVVKGT------GKEMTLGMPVRE  CG17018\_Dmel\_45551016                  NSVAD-PLFQISREVIELLK---MSPKST----MKFN--RFIPAYHNHFGK----------QCRVADYGYTKLIELFEALS----NVVQIMGD------GENRQITLSHRI  CG17018\_Dmel\_45551016\_2                TNSELEKTCVFAGEMVELFQ---NALQYT----ILFQ--KFVRSYHYHFAY----------QCRLSDYGFLKLADLLDAIN----GLVEMKLTS-----DEDKKIVLSPQV  PHYPADRAFT\_134054\_Ppat\_162679782       SWVQPGDVTGLKKQLVWLLK----QNGGK----MTLV--KVPAEYNKHYGR----------PLYKSEYNVLKLVHLLEKMK----DTLVVKGD------GTNKTLHLIEKV  PHYPADRAFT\_112426\_Ppat\_167999628       LWAPPGDLIGLKRQLVQLLN----QNGGQ----MMLS--KVPAEYNKLFGR----------PLYLADYNAQRLVHLIDKMK----DALLIKGD------GTSKTLHVLKKE  LOC100177790\_Cint\_198415080            ------NLVNFAREMVDLLK---TFSYSC----LPCH--DIMLAYQRHYGH----------ALRMN--GFTNISDMLLSIP----HVVQ-IMGP-----SNNQLLTLTHRA  LOC100177790\_Cint\_198415080\_2          TLTHRAQVKRFTQEIIKLLK---ASPDKA----ILVA--QLPDMYFRTFDR----------VWNLRDYGVCVLSDILDDIP----DGNIGVSGE-----GDNIVMSLPTRE  LOC100177790\_Cint\_198415080\_3          TSEEIQRTNLFSRELVEILR---LRHRCR----MSFT--EFVPAYHRQFGR----------QCKLSNYGFNKLLDLFESLP----HVVQ-VVED-----APEKYLQLTNEE  LOC100177790\_Cint\_198415080\_4          QLTNEEQLKVLTSQIVGLLRQ--QVGEAEGDG-IPLN--RIQSMYSHKYGF----------PLVPRYYDCEDIQSLIMLLS----HAVKLTKSL-----NNELIVKQSERK  LOC100177790\_Cint\_198415080\_5          TMISLKSIYQRAKRIRSILL----LQGGT----MTLG--EFNQCYKSRYGE----------PLDVEQHGFRTCEGMLRAL-----SLVLVVKGF-----GIKKTLVLRNHM  AT2G15560\_Atha\_30679459                -WVAPGDLNGLKGQLVKLLE----LSGGC----IPLM--RVPSEYQRKFSK----------PLFVSDYGVAKLVDLFKKMS----DVIV-VDGK-----GNKRFVYLRNSK  AT2G15560\_Atha\_30679459\_2              SVQSERNLEEFKFELQDILV----SYCCQ----VQMD--CFEAIYKLRYKR----------PLAYTNMGVNHLEQLFDKLR----DVVAIHEDP-----ATGRKLISPV--  PHYPADRAFT\_173873\_Ppat\_167997905       SWVQPGDVAGLKKQLVRLLK----QNGGK----LTLV--KVPAEYNKQYGR----------PLYLSEYNAQKLVHLLEKMK----DALV-VKGD-----GMNKTLHLIEKV  AT3G52980\_Atha\_15231722                HVVSPGSLEKLEREIIELLK---SRRGAP----ISIA--FLPMMYHEKYGRSLQAEGYLTESQRHGKAGF-SLTKLLARLKN---TIRLIDRPH-----GQHSVILAEDVS  PHYPADRAFT\_119974\_Ppat\_162690707       DGMAPGSLERLEIELQELLR----GRRAP----VSIA--SLPQLYYERFGKTLQAEGYLTESQRHGKAGY-SLTKLLARLKN---TVTLIDRPH-----GQHAVVLAEDAH  Aano1000005602\_Aano\_Aano1000005602     DAPEPPTLRSLEAEVLAVVD---GADAGA----VLCA--NLPKLYYALHGA----------PLNCATYGEDRLSDLLGKLP----NVYVRAEAR-----GRGGTVSRRGTG  Aano1000005602\_Aano\_Aano1000005602\_2   VTAEPEKLRALAAEILALVD---EAPGGK----VLSA--NLPRAYFLKYRK----------PLDMKGYGALKMSRLLTAL-----EPYGLESCG-----GATAGVGRAGSG  Aano1000005602\_Aano\_Aano1000005602\_3   CDASPAELARLAAQILALCD---AARSGR----VLLG--NLPGDYKKAYGA----------PMPSHGVKAKTLMALLPG-------VAVGGYD------PSHVDPDNRWAR  Caps1000025558\_Caps\_Caps1000025558     ---------------------------------MPFN--KFIPSYHHHFGR----------QCRVSNYGFTKLIDLFEAIP----ETVKLLDE------QGEKLLCLTEPE  Caps1000025558\_Caps\_Caps1000025558\_2   CLTEPELLIVLEEQILALLS---SCINGE----LPVP--EFLASFMKFHGH----------SLRLPDYGVTSVVELIEKVP----DVACVRNH------NGRLLIALVDHS  Caps1000025558\_Caps\_Caps1000025558\_3   TLVKLCPLQMFARDVRSLLL---A-NQNH----LPLC--NFEASYANHFGV----------ALVPASYGFPTPVDLLQAIP----HVASIHGK------GLRRMVWLHQNF  TA11490\_Tann\_84995728                  NKMNQEELSKIRRDLLSLFN-----VESK----IPLS--SLPLLYRRVHGV----------ELRYRDLGYDKLSDFIKNEV----PMCRITIH------SNQYVATICSDE  GSPATT00035725001\_Ptet\_145496001       DNNIKMKIEELKEQLIQLLL---ESQDGYS---VSFT--QIPKLLKKRVNF----------KINLIDLGFPKLRNFIESIK----EEISIEKN------GRNNVIVKLDRI  GSPATT00035725001\_Ptet\_145496001\_2     DLSNQAISSNILELLKNILS---QHKYG-----ISIN--ELYYDLSQLLGE----------WFNFKKFQCQSFFQFLQNYA----ENILIIVCQ-----KGNQYLIYERDL  TTHERM\_00129230\_Tthe\_89298162          SKEQIEKIQLIKTTILEILL---ENKRG-----VSLA--RLPKLIQKKIEF----------TFDLHELGFPKLKSLLQTIA----DVIIENDG------TTQAQAVLKQNL  Aano1000008826\_Aano\_Aano1000008826     EAGRADAGAELADEVLELVD---ESPSGT----ILCA--NLPQHYKEKFGK----------LLDLKARGGAKTVLSRLPGV----R---FEGT------FDARVSREHASR  Aano1000008826\_Aano\_Aano1000008826\_2   CTASPEQLDGLATEVLRLVD---AAPSGT----VLCA--NLPRLYYLKYRK----------ALDLKAYGASKMSALVSKLP----GVHFGGTH------RAEVSRRGTGLG  Aano1000008826\_Aano\_Aano1000008826\_3   CAASPEQLEALAREVLGLCD---AAPSGA----ILCA--NLPRLYYLKYRK----------PFDLKAYGAAKMAAVVSRLP----GVHYAGTH------RAEVSREGTGLG  Pram1000012897\_Pram\_Pram1000012897     DAALAQEFSILKLEVHKVVSR--YNASGK----VPLS--EFPAAFEKSMHK----------PIVPGTFGVKKLRTLLGLLE----DVVEIVPP------QREGEVETVQLC  TTHERM\_00353270\_Tthe\_146181541         GLQRDVTVKHLQDALLEILQ---EYPKG-----VSLV--QIPQYLKKKVYF----------QFNLQDLGFPKLKNFIQTMND---KVQIELAG------TNNSYAILKCNA  TTHERM\_00353270\_Tthe\_146181541\_2       LEKLNELLEKVKQNIEAILK---QNPNG-----IHIK--DLYSALSKSMGV----------NFQFGLFGSMDFLGFLTSYAE---SIIDIECK------QNYYVIYQKNHR  PF14\_0367\_Pfal\_124809304               -NHQENSINLIKSRISYLLY-SFSEDKTKSKGSFSLS--RLPLIYKNVYKE----------NINIDQIGYSKLTEFINNEM----DICYISTQ------HKFQCILPLVME  PHYPADRAFT\_78748\_Ppat\_168025460        LNTFKEWLPGFLGRVSMRLK---EGEW------YPLS--SLKGDFRAICGM----------ELDHVSLGFEKLSDFVRSFS----DLCRMKIVP-----VGRGPATHMVLL  TP02\_0125\_Tpar\_71030104                NRLDQEQLSKIRTEVLSLFN---IESI------IPLS--SLPLVYKRVHGV----------DLKYRDLGYEKLSDFIKNEV----PTCRITTH------SNQFVATICSDD  LOC100201018\_Hmag\_221126877            FKSKGYSLSSFQSEIILLLK---MSPKFE----IPWN--RFCLHYNLFFGR----------ELCFQDFKCNDLEELISKVS----EIVKTGNI------FYPTIVQDFSTK  GSPATT00033531001\_Ptet\_145490150       QHEKQNKLKAVQQAIIDILN---EYTKG-----VSLA--RLPKMIQRKIQF----------TFDLHELGFTKLKCLIQGID----GISIINDG------TTYASLILDEYY  Pmar\_PMAR021063\_Pmar\_239897854         TASQFRLPPELIEELKELVR---EHGLSD----SPAG--RLADSYRRTFGK----------RAPVQEYGFKNFVTALGSIP----GVSTWEEK------PQQIWFSWDTEE  GSPATT00002404001\_Ptet\_145527744       DPQIKIRVQELQEQLIELLL---ENPDG-----VSLA--QIPTLLKERVNF----------ELNLIELGFPKLKNFIESVK----DVITIENS------GRNN-FIAKLNY  GSPATT00002404001\_Ptet\_145527744\_2     ESFNQKYIFTVLQMIKTILS---KHKYG-----ISIN--QLYYDLSQQLGE----------WFNYQRFQCQSFFQFLQNYA----ENILVIVCQK----GNQYLIYERDLR  AT5G64710\_Atha\_15238228                ERSRSKVIADCHKLIKKITE---ENAGG-----YSIT--KFKKAFLEKFGY----------RLEYRKFGFSKLQSLIEMMP-----EARIE--------SGHIVTSSTPVP  PHYPADRAFT\_162624\_Ppat\_162687869       VGGRVRMLRHLRCEVAFVLD---KYCKGKPKAAMTFE--QFELLYELEMGH----------PLVSSHYGFATLKSVLQSMP----DLVSVRNI------SSEAADWRVFPY  PHYPADRAFT\_94425\_Ppat\_162669028        KQASSTHRLKHEIRMIFGCPE--YMENG-----ITVQ--EFSTVYKQRTGQ----------LPKLGEF--DSVRSMLQAMP----DVVNFEVRS-----TSDDQKITLVQS  AT5G09840\_Atha\_15242519                VVNLRPIPKEVVDKIRSIVSL---YPKG-----AAIT--ELRAELSKS-NL----------AIDKDFYGHKKFSKFLLSMP----DILQVTTV------SEGLFMIRAVTE  AT5G09840\_Atha\_15242519\_2              ERYKSNAIADCQKMIKKITEE---HPEG-----YSLI--RFRKDFLEEYGY----------HLAVDKLGYDNLQSLIRVM-----HGVRIA--------SGYIFPSTPSPN  PHYPADRAFT\_91454\_Ppat\_168048576        QSCNPSHKLKHETRMILGSPD--YMENG-----LTIE--EFRTVYMDRTGGR---------PLSLGE--FKSIESLLHAMP----DIVSFELGP-----APDDQIITLAQS  AT3G28880\_Atha\_186510546               GDSVPTWLKTFVTWLPLYLRDTSANWKGAG---YPLS--SFKADFRAVFGM----------DLDHTSLDFPKHIDFVKYFP----RLCQMKVVP-----IGKLGAATHWVM  CMU\_030530\_Cmur\_209882957              QGIHFLLRRWIWKRQQSHLPA--KSPLDF----LPLE--SLEAEFIQFFDI----------PLDIELLGWTNLLHFVEAFP----DIWTVENV------GPDEFTLIPLPH  cgd2\_2940\_Cpar\_46227797                ----FLLRRWISKRLAPHYIP--KSSLDF----LPLD--ILESEFSQFYEI----------PLNIEILGWTNLLHFVEAFP----DVWTVENV------GPDEFTLIPLPY  NE0665\_Neur\_30248674                   ---PETKQAFPRKFVLAALEQS-SDDAG-----WANLG-NFGNYLNKLQP-----------DFDSRLYGYKKLSDLVKART----DLFVTEERQVPG--STQKALYLRAKL  AN9016.2\_Anid\_67904058                 PARHAHADGHLKTLLHTTIETA-SDDDG-----WAELS-NVGTLLTKKHP-----------DFDSRTYGYNKLGDLISAL-----SLFDVVRQLPRK--GKAGKIYVRDKR  HRM2\_48560\_Daut\_224371902              NIPRESDETELKAILVSAINAV-SKDDE-----WAPLA-GVGGYINKKYP-----------SFDSRNYGYDKLGKLVESL-----PFIAVDRRKPDEN-ASIVHLYIRATT  Mbur\_0520\_Mbur\_91772560                PTNELKMDTNLVTLLRSAIEDS-ADDNG-----WASLG-NVGQNAMNRES-----------DFDSRNYGYKKLSDLIEAI-----DLFEIEKR------GKNSHIHIKDSR  Rmet\_3395\_Cmet\_94312326                TTQELRQDSRLVRLLQSASQAV-SDEDG-----WSTLG-GIGNYIAKQAP-----------EFDSRNYGYGKLSELVTAT-----NLFEV--ETRNG--GNNKTLWVRLKK  CHU\_1453\_Chut\_110637857                EEPISKVDNETIRVITESVNDL-ADEAG-----WTFLG-SLGNYILKKKP-----------DFDPRNYGFPKLYPLIKDI-----DKFELDERETGI--KNIRHIYLKEK-  Dvul\_2871\_Dvul\_120603909               KADESAASKELLKLLRKAVAAV-SGEDG-----WASLS-NLGSHISKIRP-----------SFDSRNYGYSKLSTLVKNL-----DIFET-RMVPSAD-KLHTDVFLRLKD  Franean1\_2768\_Fsp.\_158314589           PGSRLRSDTKLVTRLRDAVEEA-SDDDG-----WASQA-RVGTIIRRRTP-----------DFDPRDYGYRRLGELITAI-----ELFEVERRSNSG--GKPTLVYLRDRR  cce\_0542\_Csp.\_172035458                GNEKITTNKKLLELLKSAYDSV-ADEEE-----WVHLG-PFGSQLTKLSP-----------SFDSRNYGYKKLSELVQSV-----DIFTIK--------KTRFHLMIKLK-  WS1458\_Wsuc\_34557802                   DSWSKSESKALKSIIQPAIKAT-MKEDD-----WVPLA-SIGLYISKNHP-----------AFDPRNYGYDKLGRLIETL-----DFVEL-KKIPFKDGSSNTHVMVRLRS  HRM2\_39800\_Daut\_224371038              KTINLKSDTKLINLLRQAIEAT-EEENG-----WAALG-PVGSHISN-KT-----------SFDSRNYGYKNLSSLLKPI-----DLFELKR-------GPGNSYLVRDAR  DSY1520\_Dhaf\_89894266                  TKDGMASLDDLIRTIRIIVTES-SDEDG-----WAFLG-EVGKRLNKRYP-----------DFDTRNYGHTKLTPLISSL-----KQFEIQPRKTSN--PNIIHYFIKNKP  CKL\_2033\_Cklu\_153954651                PKVGMTSMDKLIEAIKTIITEI-SDEDG-----WAFLG-EVGSTLNKRYP-----------DFDTRNYGYSKLTPFVSSL-----KHFEIRSIRTSN--PSISLKYIRNKD  Daro\_0095\_Daro\_71905737                VKNSAAKKPLPIQFVVQALDQA-EDDDG-----WANLG-AFGSYLTKLQP-----------DFDPRLYGFKKLSDLVKSKP----HVFTVEERPVAG--SNIKVVYVRRTP  Tola\_1311\_Taue\_237808074               PPRSIKSDTKLINLLRQAIEAV-EEDDG-----WARLS-PIGSHISN-HA-----------SFDQRNYGFKKLSDLFMAI-----DLFEMKK-------TNGSVYWVRDKK  RB4721\_Rbal\_32473273                   SVSSLQKNTKLMNTLRTAVKEA-ADDDG-----WAELG-PVGAHISNQGP------------FSHRTYGFPKLSDMFDAI-----DLFEVQKTQQAG--PGSVRVRLKR--  NEMVEDRAFT\_v1g224852\_Nvec\_156200154    ESEIDKITKKEIKLISATINDL-SDEEG-----WAFLG-DVGSLLQKKQP-----------NFDSRNYGFEKLTPLIKSI-----GSFEIEQRENPK--SRYKLIFVKNK-  Psyr\_3535\_Psyr\_66046764                AAEPSKKPKAPVAFIAKIMDDI-ADEDG-----WAHLS-ALGTNITKLRP-----------EFDPRTHGYKKLSDLIKGYP----QTFELQARAAS---GGTAVLYARHKL  Rpal\_4482\_Rpal\_192292848               PAKPLQPPSAATPIIERVIKQM-ESEDG-----WVSLG-EVGKHLSNLAS-----------DFDPRTFGFRKLSDLVRKT-----NAFELEQ-------QNGHSMRIRLKP  XAC3820\_Xaxo\_21244544                  DATALRRDTRLVQMLRNAIVSA-CEDDG-----WALLS-AVGKQVAN-QA-----------SFDPRNYGYRKLSDLVRAI-----GLFEI--R------QDEQALWVRDTP  lpg2067\_Lpne\_52842281                  KANEPSAQEFPKEFILEALNNS-YDDTG-----WAYIG-TFGGYLTKLKP-----------DFDSRLYGFKKLSDLVKGKP----EIFETEERVNPG--SNTKVLYLRGRQ  slr1870\_Ssp\_16330259                   TGKELKQDAKLISLLRSAISST-IDDDG-----WSSLA-EIGGHIKN-QA-----------SFDSRNYGYAKLSSLFEAI-----DLFEIE--------RKNRAIYVRNRQ  Rleg\_6293\_Rleg\_241554076               VPAKPRLDAAALKMLENAVIAS-ADEDG-----RANLA-RVGAHLAKQSS-----------DFDARNYGFARLTDLVEAS-----GIVDVERSG-----DSPKIVTVRMKG  CAP2UW1\_2580\_CAcc\_257094155            SLAALARPPFPEKFLIEALDKS-VDDSG-----WSQLG-TFGSYLQKIQP-----------DFDSRIHGFKKLSDLVRART----DLFTTEERPALG--SNYKVLYVRART  Tola\_1344\_Taue\_237808105               ENKQSFAKKFPREFIVEALENS-YDHTG-----WAHLG-TFGSYLLKLQP-----------DFDSRLYGFKKLSDLVKTRT----DVFQTTERTIPG--TDTKVLYIKVR-  blr3164\_Bjap\_27378275                  RSTSLQPLDAATPIIKKVITQM-ESEDG-----WVALG-EVGRQLANLAS-----------DFDPRTFGFRKLSDLVRKT-----NSFEIDE-------SKGRSMRIRVKP  RSP\_1459\_Rsph\_77461998                 RGREKEEPMKAIPLIAAAMRAI-DPEGE-----WYSLG-QIGQFITQANP-----------DFDTRTYGSAKLSDLIKKI-----SRFEVKP-------GPGGQLLARDIA  CT0467\_Ctep\_21673303                   SSHELRSDGKLRTLLLNAVDSA-QEEDG-----WSNLA-KIGKNIAN-QA-----------SFDPRNYGFKRLSDLVRAI-----DMFDIEFR------NNNSELYIRDKR  RCIX466\_Umet\_147920970                 ARTHKKLDKFAINLLKRAYEMG-VSKEGE----WVDLS-TLANNLYKIEP-----------SFDSREYGFSQLQLLVKAT-----NLYDVERSRL----KGPSQSYIRLK-  BPSS1326\_Bpse\_53722351                 KHDGDARKAEAISLAVETFDAL-ASERGESGKIWASVLKSA---IKRRKP-----------DFNESYYGFRAFGNLLEEAQAR--GLLEIGRD------DKSGTFVFRPIQ  azo2662\_Asp.\_119898953                 KEELAARKSQAIEFAVETFEAL-LAERGDSGKIWASMLKEA---IKRRKP-----------GFNETYYGFRAFGNLLEEAQAR--GLLEFGRD------EKSGAYVYRSNA  CAP2UW1\_2449\_CAcc\_257094026            KAELEARRSKAIELAVATFDAL-VAERGDSSKIWASMLKEA---IKRRKP-----------DFSESYYGFRAFGNLLEEAQAR--GLLEIGRD------EKSGTYVFRSNG  Daro\_3189\_Daro\_71908802                RDKQEERKSKAIDIVSATFVDL-MADRGESERIWASVLKEV---VKRRNP-----------GFNESYYGFRTFGNLLEEAAGR--GLIGFGRD------DK-GAFVFRAPP  Oter\_3120\_Oter\_182414934               EKPAEPDPMDAIELLVATVGAL-TEERGEDEPIWASMVKQA---IKRRNP-----------GFNERAHGFKSFTALLHDA-----EKRRLVQLE-----EKSGNCQVRLAE  Noca\_0118\_Nsp.\_119714385               ADAEADSLPDLRTILEAAVRST-AQDDG-----WSALG-SVGSYLGKTHA-----------AFDPRHYGFPRLSALAGAQ-----PYLDLDHPD-----GGQPRVRLRSQR  Swit\_5232\_Swit\_148550669               GAKSLAQDSKLITTLRGAVEAA-ARDDG-----WAHMA-AAGSAVKRQAP------------IDPRNYGVKNFPRLFEAT-----GLFDIMK-------GDNGQTYVADKR  BT\_2650\_Bthe\_29348060                  ENTPEFFIQRDMEYFDKAFEQA-ADGKAE-----VSLS-LIGGVLKKLMP-----------KFKVKRYGCRTLGKLYERL-----EKYELVKT------GKGVAGLVRIKS  CJE0182\_Cjej\_57237194                  SKKEDILSKDYLRALINITEQL-IDEKG-----RAEYA-QIRTNMNRKYS-----------DFHPQNYGFKNFRALIQK-------FLPKMKKFEE---EREKNIYFLVKK  TPASS\_0894\_Tpal\_189026117              AVEEDTGGFDLGKLIAHAYRNSRMTEEG-----WVSLS-NLGKSLRITKP-----------EFDPRSYNHSTLREMVEALP----ELFEVQSDR-----RIPPNYWVRAVR  Amuc\_0741\_Amuc\_187735246               SATKKDPLEIIHKHLQQCAIDN-KKDDG-----YTSAS-DAGKYLRQHIL-----------GFTPKTYGYKNLTNLLEDFR----NLYAIEKH-----PIPGNKNSVKLCN  Aave\_4318\_Acit\_120612954               PTPELRQDTALMNLLRDAVAAT-QDESG-----WAKVG-AVGTQIAN-KA-----------SFDARNYGYATLTKLLAAT-----QAFEMRD-------EGTSKVSVRDRR  MSMEG\_1210\_Msme\_118470413              EPEPPDPQATATALLLRALQIG-LEKDDVE---WLHNS-AVKAQMKRMDP-----------SFSEKSLGFRSFSDFLRSRS----DYVELD--------ESSTIRMVRLRP  Cagg\_1924\_Cagg\_219848822               QVVITRPLRDAVKLLQRILTES-AQTAPGE---WVSGS-AIGSRLRAIDP-----------SFDHRFYGYQNLSKLLDACVVEAKEMFEIRR-------RDNGNIDIRLKP  Tmz1t\_0994\_Tsp.\_217969422              DGTVDWPAAGIVRALREAAAQL--AVEG-----WTPIA-AAGRWIADRHP-----------EQLPANYGCSSWRQVVHECR-----LFELRYREV----EGQRAAWYRPRE  GAU\_0654\_Gaur\_226226060                TGEDETTRWDPWQLVTEAIGRM-KRNGD-----VMRSD-RLKQVMQEIDS-----------SFDEKNLGHPKFSRFVQEAQQR--GLLHVTK-------LDSGQLEVDAPE  PSPTO\_0007\_Psyr\_28867250               DGIVHWPSSGITACLREAETKL--AHAG-----WTPLF-EAIRWIAKTYP-----------ELTPKRYGCGSWRHVIHES-----QQFEIRKQSQAD--NGPTVVWYRSRP  Tmz1t\_3165\_Tsp.\_237653824              GAGVEWACSTIVRLLREAEAGL--AHDG-----WTSLN-DAIAFLQKEHP-----------EHTPRRYGCSSWRQVLHES-----GQFEVRKE------QAGPGLPTRVWF  ebA2464\_Aaro\_56476823                  DGTVDWPAVGIVRALREAAGEL--AVEG-----WTPVA-AAGRWISERHP-----------EQLPAKYGCSSWRQVVHEC-----RFFELRYRDV----DGRRAAWFRARE  MXAN\_0030\_Mxan\_108762087               GGGKSQPKTEVPDIAREVVQSMLARATGP----VNP-S-LIKEAIVRKEP-----------DFDEREHGFSTFARLLAAL-----EQQGLLRRI-----QQGRQWYVVAAD  Rsph17025\_3537\_Rsph\_146279554          HGQVPWHVTTIAQALVDAASEL--APGG-----WTSVE-AAVNWVLARRP-----------GERPEMCGCRSWRQVIHES-----GLFDLQVREV----DGRRQAWYRPRN  Bfae\_18070\_Bfae\_257068962              DESGEDPREAATRLLQRALQ---LGDRGDSE--WLHAS-AVKSHMRRMDS-----------SFSEKAIGYRSFNDFLKGNA----QIAELE--------ESGHERLVRARE  Acid\_7883\_CSol\_116626906               HKRVALDLAQAMPLVERALGVL-ERREV-----RPQLG-LLKSTMLQLDS-----------TFNEKAYGASSFTDFVEKLQKA--DFVEVT--------GGEGRYMIRMKG  Acid\_7883\_CSol\_116626906\_2             KPGSKPEEAIPLLRDILEIHRF-DVDSG------STAD-ELFEWVKQERS-----------SFDPREYGFQEFSELLNYAQDK--GLVRNQV-------DEERGLIVFLGA  Ccur\_05410\_Ccur\_256826985              SGENSISKRDVADAIVNIINEG--NGQG------QHLG-SVGSRLVNVYP-----------DFDVRSFGYSQLSKFLESF-----DNLQLD--------RRDNAVFVSLKD  Ccur\_05410\_Ccur\_256826985\_2            LKDTDRLRDQVDAFVVDLARRA--GAEG------VNLG-YLGGLIHSSFP-----------DFSVKDYGYSKLSKFIRDV-----EGVLLVDD------ESGTRVVIDCE-  AN9016.2\_Anid\_67904058                 PARHAHADGHLKTLLHTTIETA-SDDDG-----WAELS-NVGTLLTKKHP-----------DFDSRTYGYNKLGDLISAL-----SLFDVVRQLPRK--GKAGKIYVRDKR  consensus/85%                          ..........h...l..hh........s.....h......h...h.p..s............h....hGh.ph.phlp.........h................h......      Legend is  Aaro : Aromatoleum aromaticum; Acit : Acidovorax citrulli; Amuc : Akkermansia muciniphila; Anid : Aspergillus nidulans; Asp. : Azoarcus sp.;   Bfae : Brachybacterium faecium; Bjap : Bradyrhizobium japonicum; Bpse : Burkholderia pseudomallei; Bthe : Bacteroides thetaiotaomicron;   CAcc : Candidatus Accumulibacter; CSol : Candidatus Solibacter; Cagg : Chloroflexus aggregans; Ccur : Cryptobacterium curtum;   Chut : Cytophaga hutchinsonii; Cjej : Campylobacter jejuni; Cklu : Clostridium kluyveri; Cmet : Cupriavidus metallidurans;   Csp. : Cyanothece sp.; Ctep : Chlorobium tepidum; Daro : Dechloromonas aromatica; Daut : Desulfobacterium autotrophicum;   Dhaf : Desulfitobacterium hafniense; Dvul : Desulfovibrio vulgaris; Fsp. : Frankia sp.; Gaur : Gemmatimonas aurantiaca;   Lpne : Legionella pneumophila; Mbur : Methanococcoides burtonii; Msme : Mycobacterium smegmatis; Mxan : Myxococcus xanthus;   Neur : nitrosomonas europaea; Nsp. : Nocardioides sp.; Nvec : Nematostella vectensis; Oter : Opitutus terrae;   Psyr : Pseudomonas syringae; Rbal : Rhodopirellula baltica; Rleg : Rhizobium leguminosarum; Rpal : Rhodopseudomonas palustris; Rsph : Rhodobacter sphaeroides;   Ssp : Synechocystis sp.; Swit : Sphingomonas wittichii; Taue : Tolumonas auensis; Tpal : Treponema pallidum; Tsp. : Thauera sp.;   Umet : uncultured methanogenic; Wsuc : Wolinella succinogenes; Xaxo : Xanthomonas axonopodis;  Amel : Apis mellifera; Atha : Arabidopsis thaliana; Bflo : Branchiostoma floridae; Caps : Capitella spI; Cele : Caenorhabditis elegans;   Cint : Ciona intestinalis; Cmur : Cryptosporidium muris; Cpar : Cryptosporidium parvum; Dmel : Drosophila melanogaster; Hmag : Hydra magnipapillata;   Hsap : Homo sapiens; Nvec : Nematostella vectensis; Pfal : Plasmodium falciparum; Pmar : Perkinsus marinus; Ppat : Physcomitrella patens;   Ptet : Paramecium tetraurelia; Tann : Theileria annulata; Tpar : Theileria parva; Tthe : Tetrahymena thermophila |

  
  
  
  
  
Top  


---

Multiple
Alignment of LK-Nuclease (DUF88)  
  

|  |
| --- |
| 1exnB                                         EEEEEEHHHHHHHHHHHHH--------------HHHHHHHHHHH-HHHHH----------EEEEEE--HHHHHHHH---HHHHHHHHHH-HHHHHHHHHHHHHHHHHHHHHHH----EEEEEEE---------------------HHHHHHHHH----------EEEEEE-------------------EEEEEE-----------------EEE----  1exnB\_BPT5\_2392326                            RNLMIVDGTNLGFRFKHNNSK----------KPFASSYVSTIQS-LAKSYS-----ARTTIVLGDKGKSVFRLEHLPEYKGNRDEKYAGRTEEEKALDEQFFEYLKDAFELCKTT-FPTFTIRG---------------VEADDMAAYIVKLIGH------LYDHVWLIST--DGDWDTLLTD------KVSRFSFTT-------------RREYHLRD  LKAP\_Hsap\_254763460                           PIGVFWDIENCSVPS----------------GRSATAVVQRIRE-KFFKGHR----EAEFICVCDISK-----------------------------------ENKEVIQELNNCQVTVAHINATA------------KNAADDKLRQSLRRFANTHT---APATVVLVST--DVNFALELSDLRHRHGFHIILVHKN--------QASEALLHHANEL\ Limkainb1  LOC100183300\_Cint\_198415078                   PIGVFWDIENCQVPS----------------GKSAMAIVQKIRR-QFFNDHA----EAEFMAVCDINK-----------------------------------ESRHVIQDLNNAQVNVIHVNAVA------------KNAADDKLRQSIRRYAQTHT---APATVVLITG--DCNFTSEVSDLRHRHKYFVVLMHPV--------NSSKALVEAANT- |  LOC661475\_Tcas\_189240101                      PLGIFWDIENCQVPK----------------NTSASAVVQRIRE-FFLEKYR----EAEFLVVCDVKK-----------------------------------ERPQVIQELHDSQVNLIHVASTS------------KNAADEKLRQSLRRFAEVFP---APSAVVLISG--DINFAADLSDLRYRKKIRVILVHNT--------NVADALILCANE- |  \_Atha\_9759464                                 KTSVWWDIENCEVPK----------------GCDPHGVAQSIRS-VLSKSNF--CGPLTIYAYGDTNQ-----------------------------------IPSSVQQALSSTGVSLNHVPAGV------------KDGSDKKLLVDIMLWAMDNQ---APANIMLISG--DKDFSYLLHKLGMK-RYNILLARPE--------KASTPLIAAAKTV |  EDA32\_Atha\_15228710                           KTSVWWDIENCQVPK----------------GLDAHGIAQNISS-ALKKMNY--CGRVSISAYGDTSG-----------------------------------IPHVIQHALNSTGIELHHVPAGV------------KDASDKKILVDMLFWAFDNP---APSNIMLISG--DRDFSNALHKLSLR-RYNILLAHPP--------KASAPLSQAATTV |  AT2G15560\_Atha\_30679459                       PMAILWDMENCPVPS----------------DVRPEDVASNIRM-AIQLHPVISGPVVNFSAYGDFNG-----------------------------------FPRRVREGCQRTGVKLIDVPNGR------------KDASDKAILIDMFLFVLDNK---PPATIVLVSG--DVDFAPALHILGQR-GYTVILVIPS-------SVYVNSALSNAGKF |  AT5G64710\_Atha\_15238228                       RVPVWWDFENCHLPS----------------GANVFKLAQTITS-AVRICGI--KGPITITAYGDMIQ-----------------------------------LSRTNQEALFATGINLTHVPQGG------------KNSTDRSLITEIMCWVSQNP---PPAHLFLISS--DSDFANVLHRLRMR-NYNILLACYE-------ETTLGVLCSAASIM |  AT3G61028\_Atha\_145332911                      KTEIWWDVDSCRLPD----------------SVDPYRLVGNLRK-SLNEKGY-RGPITSINAFGNTNR-----------------------------------IDETTMLALSATGVYTRHIPDGR------------KESAHKKILVDLLCFGMDNIQ--QPCNIMLISG--NRDYSDSLHQLKSR-LFNILLAQPE-------DFASTPLIHAASTV |  AT4G30760\_Atha\_30688791                       KTCVFWDVEDYPIPA----------------DLHPRSIRRRIVK-DVKKYGC--DAEVSIHAYANDNT-----------------------------------VSVTMRRQFSAAGIKLEVFTQGD------------KYARHCSLYGDIMLWSLENP---PPSNIIVIAKIIDDDLADRIGCLTTVWSYGLLISQVK-------PEWLERLFPVGSY- |  RCOM\_1343910\_Rcom\_255548285                   KTSVWWDIENCQVPK----------------GCDPHAIAQNISS-ALVKLNY--CGAVSISAYGDTNR-----------------------------------IPQSVQHALSSTGIALNHVPAGV------------KDASDKKILVDMLFWAVDNP---APANYLLISG--DRDFSNCLHQLRMR-RYNILLAQPQ--------KASAPLIAAAKTV |  DDB\_G0269710\_Ddis\_66825719                    PVNVFWDLENCAVPS--------------------NLKGIHVVN-AIRSFALQRGVLKNISAFANLKL-----------------------------------IKDELRSNLQECGVLLHDVTRNK------------SNASDIAILVEILKLVIDNK---PPHCIVLISG--DRDFSNVLNTLTFR-RYQVFLIHST--------HASDVLKYSATAS |  An09g02040\_Anig\_145241746                     KFAVLIDADNAAYSV-----------------------IHPLLA-EIARYGT----AHAKRAYGDWSSP----------------------------------NLTRWKDQLLQHSIEPIQQFAYTYG----------KNSTDSAMIIDAMDLLYTR----RYDGFCLVSS--DSDFTRLAARIRES-GLVVYGFGEQ--------KTPKPFVAACDKF |  FG08118.1\_Gzea\_46127481                       PVCIYIDDSNVAIRGRA-----------MHDPQGTLTPWNYDID-VLANIIIQQFDLTAIEPFVQKSLNFYGADLHRSP------------------------QLDHLRGLGLVYGCDCPRNGHGH------------EKQADVALATDMTEQAKHALDFGIARDFVLVSG--DSDFIPAVRKVLGY-GFNVHVWAWR-------RSLSSEFYRLKQDF |  AN9016.2\_Anid\_67904058                        NLVVLVDADNAMPST-----------------------ARLILA-EVAKYGT----VYVKRAYGDWTST----------------------------------SLKGWKKELLSQSIQPFQQFAYTNG----------KNSTDSAMIIDAMDLLYSN----RFGGFCLVSS--DSDFTRLAARIRES-GLTVYGFGEQ--------KTPKPFVAACNKF |  NECHADRAFT\_62469\_Nhae\_256725266               KLAVLIDSDNVTPRV-----------------------AGQVLA-EVAKYGI----AFVKRAYGDWTGP----------------------------------GLNSWKDHLLDNSIQPIQQFSYTKG----------KNSTDSAMIIDAMDLLYSN----KFDGFCLVSS--DSDFTRLASRIRES-GLVVYGFGER--------KTPNSLVRACDKF |  ATEG\_05392\_Ater\_115397957                     SPWILIDADNAQASV-----------------------VNLVLS-EVAKYGT----AHVKRAYGDWTSS----------------------------------NLLPWKAKLLEHSIQPVQQFAYTHG----------KNATDSAMIIDAMDLLYAN----RFDGFCLVSS--DSDFTRLAARIRES-GATVYGFGVH--------NTPKPFVSACDKF |  PTRG\_11053\_Ptri\_189210104                     SLALLIDGDNVSPKI-----------------------IAGLMA-EVANYGT----ASVRRIYGDWTSP----------------------------------YLNGWKACLLHHSITPIQQFAYTTG----------KNVTDGAMIIDAMDLLYTG----RLSSFCLVSS--DSDFTRLAARIREQ-GVTVYGFGER--------KTNNAFIAACDKF/  RCIX466\_Umet\_147920970                        NIAMLIDGDNAQPSL-----------------------IEEMLS-EAGKYGN----VTVRRIYGDWTMS----------------------------------SMNSWKDYLHKHAFQPIQQFRYTKG----------KNATDSAMIIDAMDILYAG----HVDGFCIVSS--DSDYTKLATRLRES-GLFVMGIGRK--------DTPQSFVKACVIF\Archaea  Mbur\_0520\_Mbur\_91772560                       KLAVLIDADNVHSSI-----------------------IKGLFD-EIAKYGI----ASVKRLYGDWTGP----------------------------------QLSNWKDNLHIYSIQPIQQFSYTAK----------KNATDSALIIDAMDLLYTR----NLDGFCIVSS--DSDYTKLCQRIRES-GAFVYGFGEK--------KTPEAFRAACNKF |  MJ0482\_Mjan\_15668658                          RIALLIDGPNMLRKE-------------------FNIDLDKIRE-VLSEFGD----IVIGRVYLNQYA------------------------------------SDKLIEAVINQGFEPKIS----------------AGDVDVEMAVDATELVFNP----NIDTIAYVTR--DADFLPAIRKAKER-GKKVIVIGAE-------PGFSTALQNIADYV |  Htur\_0796\_Htur\_284164086                      RVAVLVDAQNLYHTA--------------QSLHSRNIDYSALLD-KAVQDRQ----LTRAIAYVIRADSP---------------------------------EEESFFEALIDIGFEPKIKDIKTFSDGTK------KADWDVGMSLDAVTLAN------HVDTVVLCTG--DGDFSRLCSHLRHE-GVRVEVMAFE-------SSTAEELIAAADSF |  HborDRAFT\_0607\_Hbor\_227879953                 RVAILADAQNLYHSA--------------QSLYSRNIDYSSLLE-KGVSDRT----LTRAIAYVVRADSP---------------------------------EEESFFDALVEIGFETKIKDIKTFGDGSK------KADWDVGMSLDAVTLAN------HVDTVVLCTG--DGDFSRLCSHLRHE-GVRVEVIAFK-------ESTADELIEAADTF |  MmarC7\_0304\_Mmar\_150402231                    KMALLIDGPNMLRKE-------------------FNVDLDKVRD-ALEQFGD----IVVGRVYLNQYA------------------------------------SDKLIEAIANQGFEPRIS----------------AGDVDVEMAVDGTELIFNK----NLDTLVYMTR--DADFLPAIRKAKER-GKQIIVVGAE-------PGFSTAIQNIADHV |  rrnAC0208\_Hmar\_55377125                       RVAVLADAQNLYHTA--------------RSLYSRNIDYEALLE-EAVDGRE----LTRAIAYVIRADSP---------------------------------EEESFFEALVDIGFETRIKDIKTFQDGSK------KADWDVGMSLDAVSLAN------HVDTVVLCTG--DGDFARVCRYLRHE-GCRVEAMGFE-------ESSSEDLKAAVDGF |  FerpDRAFT\_2162\_Fpla\_259025443                 KIGVLVDGPNMLRKE-------------------FNLNLKEIRE-ILDSYGD----VKIAKVFINQYA------------------------------------GEKLVEAIENQGFEPVVT----------------SGDVDVKMAVEAMEIIYND----SIDVLALVTR--DADFKAVLQKAMEK-GKETIIIGAE-------PGFSAALKNAADIA |  Maeo\_0221\_Maeo\_150013362                      RMALLIDGPNMLRKE-------------------FNIDLDKIRE-VLDEFGS----VVVGRVYLNQYA------------------------------------SDKLIEAIANQGYEPKVS----------------AGDVDVEMAVDGTELIFND----TIDTIIYMTR--DADFLPAIRKAKEH-GKNIIIIGAE-------PGFSTAIQNIADYV |  TK0756\_Tkod\_57640691                          TIGLIIDGPNILRKE-------------------FGIKLEDILE-ALKRIGN----VRVAKVILNQYA------------------------------------PQGLIEAVVNQGLEPVIV----------------AGDTDVRVAIEAMELIYNS----DVDVIALASR--DADFLPIIIEAKRR-GKETVVIGVD-------PGFSVALQNAADYV |  MTH1064\_Mthe\_15679075                         SLGLLVDGPNMLRKE-------------------FCSDLEFVKN-LLFDRGN----LKVGKVLLNQYA------------------------------------SDKLIEAVVNQGFSPMIV----------------AGDVDVQLAVEAFELIHNP----NIDVVAIMTR--NADFLPLINIAKEN-GKETLVIGAE-------PGFSIALQNSADDS |  TSIB\_0816\_Tsib\_242265195                      QIGLVINGPNILLSK-------------------FDISLEDILY-ALNDIGR----IVVGKVVINYEF------------------------------------SSNLLKSIIDSGLESVMV----------------NGRVDVAVAVEGMKIIYNP----RINVLALATR--DAHFMPLVFEAKRM-GKDVIVIAPE-------KKVSEALQNIADKT/  syc2204\_c\_Telo\_56752213                       RLAIFIDGNNMFYAQ-----------------QKNGWFFDPRRV-LNYFANR----PEIELVNAYWYTGLKDPQ-----------------------------DQRGFRDALVSLGYTVRTKMLKEFHDESNGNRYFQRANLDIEIVIDMFNTVE------QYDEIVLFSG--DGDFERAIELLRAK-QTHITVVSTD-------GMIARELRNATDRY\LabABacteria   Tmz1t\_1700\_Tsp.\_217970117                     SMALFCDFENVALGV--------------RDAKYEKFDIKRVLE-RLLLKGS----IVVKKAYCDWDR------------------------------------YKSFKAAMHEANFELIEIPHVRQSG---------KNSADIRLVVDALDLCYTKS---HVDTFVIISG--DSDFSPLVSKLREN-AKQVIGVGVK-------QSTSDLLIANCDEF |  Tmz1t\_0056\_Tsp.\_217505845                     --ALFVDFDNVYSGLRKLDPAIADRFARQPLEWVNWVIGELELP-DHAPAGA-RRRLLVRRCYLNPQA------------------------------------YQRFRPSFNLAGFEIIDCPALTSEG---------KTSTDIHMVLDIIDLLQHEA---RYDEFIVFSA--DADFTPVLRKLRRW-DRRTTVLAIG--------FPSAAYRASADLL |   BTH\_II1099\_Btha\_83717296                      NMALFCDFENIALGV--------------RDTKFEKFDIKPVLE-KLLLKGS----IVVKKAYCDWDR------------------------------------YKTFKAAMHEASFELIEIPHVRQSG---------KNSADIRLVVDALDLCYTKA---HVDTFVIISG--DSDFSPLVSKLREN-AKRVIGVGVK-------NSTSDLLVANCDEF |   CV\_0373\_Cvio\_34495828                         KLAVLIDADNAQSSL-----------------------IAELLA-EVAKYGT----AIVKRAYGDWTTT----------------------------------QLKGWKEVLHQYAISPIQQFAYTKG----------KNSTDSALIIDAMDLLYTG----NFDGFCLVSS--DSDFTRLATRLREG-GLTVIGLGEQ-------SKTPRPFIAACDKF |  NE0665\_Neur\_30248674                          RLAVLIDADNAQAAV-----------------------IEGLLA-EIARFGE----ATVKRIYGDFTAP----------------------------------ASASWKKVLQKYAIKPVQQFAYTTG----------KNATDSTLIIDAMDLLYTR----KFDGFCLITS--DSDFTGLAMRLREE-GLTVLGFGEK--------KTPEAFRNACHKF |  Vapar\_0857\_Vpar\_239813866                     RVMLLIDADNVSADV-----------------------IEQAVQRTLAEHGA----VHVRRAYCNAET------------------------------------ALKQQALFKRLSVRPMVNLSAG------------KNSTDIALAVDALDLVIAE----RPDVVVLVSS--DSDFAPLVIRLREK-GCRVCGLGQQ-------GKTGEETVAVYDEF |  Vapar\_0855\_Vpar\_239800441                     SMAVFIDADNLNDAT----------------------ALDHVLL-ALRSMAD---RILYRRAYGRPES------------------------------------LKSIHAVLWRHGVRPVANLIVD------------KTTTDSALVIDAVEAVCTN----DIDIVAICSG--DADFVPLAIWLREK-GCRVLCYSLA----------NKIFANPDSFY |  AnaeK\_2905\_Asp.\_197123307                     RIALFIDFENLVTRT---------------GLSAETFDLQPALD-TLLEKG----KVVYRRAYADWTR------------------------------------FSAATPRLHEKGVELVDVPPSTRAG---------KNGADMRLVIDALELAYLRE---HIDTFVIASG--DSDFCPLAYKLREN-DRTVIGMAVR-------EATSPLFVKACDEF |  BRADO3579\_Bsp.\_146340536                      RLAVLIDADNASAKI-----------------------VDGLFE-EIAKIGE----ASVRRIYGDFSSP----------------------------------RSKPWADTLARHAIVPQQQFAYTTG----------KNASDITLVIDAMDLLHSG----RFDGFCLVSS--DSDFTRLAARIREQ-GIDVFGFGEQ--------KTPESFRQACRRF |  BRADO4470\_Bsp.\_146341383                      KIALFIDGANLYATA--------------KTL-GFDIDYKRLLK-EFQSRGT----LLRAFYYTAIIEDQEYS------------------------------SIRPLIDWLDYNGYTVVTKATKEFIDASGR--RKVKGNMDIELAVDAMELAE------HIDQMVLFSG--DGDFRSLVEAMQRR-GVRVTVISTI---SSQPPMIADELRRQADVF |  BpinM\_010100011896\_Bpin\_254707641             KIALFIDGANLYAAS--------------KTL-GFDIDYRKLLK-AFQKRGY----LLRAYYYTALVEDQEYS------------------------------SIRPLIDWLDYNGYKVGTKAAKEFTDSTGR--RKVKGNMDIELTVDAKQLTD------TVDHFVIFSG--DGDFRSLVEALQRK-GRKVSVVSTL---TTQPAMISDELRRQADHF |  CJJ26094\_0215\_Cjej\_86151302                   SIAIFIDAENIPAKY-----------------------AKSIFD-IASDYGE----VIIKRIYGDWTQK----------------------------------NIQGWREQIAEYSLIAMQQFNFAAN----------KNSSDMYLITEIMSIFYEK----NIDIFVIVSS--DSDYTSLIQKLREN-KKQVIGMGLE--------KSIKSYVNAFSEF |  ACA\_1164\_Acal\_255020942                       GIGVYVDAENIRYNG-------------------GYAMRYDVLR-RFAGRGD-DARLLRLNTYMAIDEERLRRDPDYRD------------------------GIRGYQQAVRDLGWKIIEKPVRWFTDEEGN--RLSKANADLDLAVDVMLQSE------RLDQVLLVTG--DGDFLQVVRALQNR-GCRVEVLAFR--------NVSRDLQHEADAF |  Cagg\_3705\_Cagg\_219850544                      DVAVFIDFENIYVSV--------------RDKLNATPNFEAIMD-RCNDLGR----VVISRAYADWYR------------------------------------YPRITSALYANAIEPIYVATYYYDKDAGRTGRAIKNSVDMNLCIDAMKTLYTNP---NISRFVLVTG--DRDFIPLVHSIRQH-GKEVYIIGIG-------GAASTHLAQSADEF |  Cagg\_3809\_Cagg\_219544907                      NTAIFYDIENLIKGY------------GFSTQTITNVSLKEILT-ALRQTGK-IGHIAVQRAYANWSDP----------------------------------RLGVMRDEINELGIDPIQVFGFAREPK--------RNAADVQLAIDAIDLAYIRP---GLDVFVIVSG--DGGFAALAKKLHEY-GKTVIGCAYR-------SAVNKTFQAVCDEF |  Cagg\_1300\_Cagg\_219848211                      RIGVFYDGSYFSYAQ-------TYFYAEKKVGWLSFTPFHRLIE-QFISSKE-QRYAMHRIVYASWHQGLFPASQTNEKQFF---------------------IERNRHLDLMHAGIEPKYVPMAPSGH---------EKGVDVSLAVDVMERVMEG----KIDVAVLVTG--DGDLTPLARTVMKH-GVRVGVFYFEYDSPQRNSRVNGRLITACNYA/  consensus/90%                                 ..slhhD..Nh...........................h..h....h..............hh............................................h...h...sh......................ss.D..h..-h..b..........s.hhlho...D.ca...h..h..........h.............s..h...sp..                                                ......\*.......................................................................................................................................\*..............................\*.\*...........................................  Legend is  Acal : Acidithiobacillus caldus; Anid : Aspergillus nidulans; Anig : Aspergillus niger; Asp. : Anaeromyxobacter sp.; Ater : Aspergillus terreus; Atha : Arabidopsis thaliana; Bpin : Brucella pinnipedialis; Bsp. : Bradyrhizobium sp.; Btha : Burkholderia thailandensis;   Cagg : Chloroflexus aggregans; Cint : Ciona intestinalis; Cjej : Campylobacter jejuni; Cvio : Chromobacterium violaceum; Ddis : Dictyostelium discoideum; Fpla : Ferroglobus placidus; Gzea : Gibberella zeae; Hbor : Halogeometricum borinquense; Hmar : Haloarcula marismortui;   Hsap : Homo sapiens; Htur : Haloterrigena turkmenica; Maeo : Methanococcus aeolicus; Mbur : Methanococcoides burtonii; Mjan : Methanocaldococcus jannaschii; Mmar : Methanococcus maripaludis; Mthe : Methanothermobacter thermautotrophicus; Neur : nitrosomonas europaea;   Nhae : Nectria haematococca; Ptri : Pyrenophora tritici-repentis; Rcom : Ricinus communis; Tcas : Tribolium castaneum; Telo : Thermosynechococcus elongatus; Tkod : Thermococcus kodakarensis; Tsib : Thermococcus sibiricus; Tsp. : Thauera sp.; Umet : uncultured methanogenic;   Vpar : Variovorax paradoxus; BPT5 : Enterobacteria phage T5 |

  
Top  


---

Multiple
Alginment of the divergent WW  
  

|  |
| --- |
| FINAL                               ------E--------------HHHHHHHH-HHHHH-----EEEEEEE----------EEEEEE----------------EEEEE------------HHHHHHHHHHHHHHH  TP03\_0796\_Tpar\_71026501             MKRSICD-NFW--------SAAEVAYVSL-HCKNTVKL--NIELRVKGE------MGWVVYLIKEPS-----------DFKGFV-DTHSTINSYD-DYYWRQLNIFASSIL  TA18280\_Tann\_85001524               MKRSICD-NFW--------SAAEVAYVSL-HCKNTVKL--NIELRVKGE------MGWVVYLIKEPT-----------NFKGFV-DTHSTVNSYD-DYYWRQLNLFASSIL  BBOV\_III000420\_Bbov\_156087541       MKRSIAD-NFW--------SASEVAFVSL-HCQDTVDL--RTELRVKGE------MGWVVYLREKPP-----------GFLGFV-DTHSTVDPYS-NYHWRALNNFAVDIM  BBOV\_II001020\_Bbov\_156084292        VRSRFVE-FDT--------KNIPVEHIMN-ICQRRQD---IFRVEVNEE-----LNQTYIYFVKPPS-----------YFKNWI-DRNNLTDIYP-ETMWEQFLDFLVELV  TA08680\_Tann\_84996849               VRGRFIE-FNT--------EEIPPEHIMG-ICKMKPD---IFNVVSNGS-----LNETFIYLVEEPS-----------WFVNWV-DRNDLRDVYP-SEMWEQFFQFIKNYK  TP04\_0484\_Tpar\_71028952             VRGRFIE-FNT--------EEIPPEHIMD-LCKRKPD---IFNVVSNGS-----LNETFIYLVEEPS-----------WFVKWV-DRNDLRDIYP-AEMWDQFFQFIKNYK  TGGT1\_045580\_Tgon\_221485777         IKGRLRE-FGC--------SHIVNTNFIA-IYALFPT---LYRIEWLPQ-----QGERAVFFTIDPP-----------NAKGWI-DINDPHDRYP-PSMWTAFSNFLYDKF  Pmar\_PMAR023053\_Pmar\_239875739      VKKRHEERYKA--------EPLTLDTLMH-LCNQNNP---DFSIEKAED-----RPVPGFKLAHPPK-----------SFRGFV-SPTDPVDYYG-PQVWEALRTEINSLL  CMU\_005100\_Cmur\_209557542           --------YSI--------RNLTITDCFN-ICTCYPS---VFILNDSKSNSVSCNNEIVIELVHPPAKLSNINCIRLHNNKMWV-DPKEPQDIYP-NELWYSFIQYTCNLL  CMU\_006480\_Cmur\_209881271           IQERSTESFRN--------MKFGTKELLN-ELYIHKD---EFFVSESGE----------VYFTNNKV-----------EESHWI-DPKNPEDPYS-PSIWKAFMQYIHNLI  PF14\_0195\_Pfal\_124808633            IKGRADE-YEY--------REILRNNIKT-AYSLYPE---KYIIKPNES-----GDDYIIYFTNKKV-----------SEDYFM-SINNLKDIYS-SNLWKQFEEYLEEIS  BBOV\_III010230\_Bbov\_156089481       LLRICRE-DLY--------RRFDVVNISQ-VDDHHTD---K-GIQHSWA----------IMLAGKPFVQ---------REVNPS-DPRELADLFR-HAVFMASANMSDRPV  Pmar\_PMAR006821\_Pmar\_239903346      --------LIV--------DHAPPDAVAL-----EVV---FARITKSGQ-------NCCVLFRETPVVSPAF------DGSFYF-VSPHAGDIYD-KAMWDAFRLYLDEAL  PF14\_0367\_Pfal\_124809304            CREDILN--IF--------DIYKANSQEK-EDKKSKN---YYKKTYNNE-------NICIYLRGIP-------------RDYFI-NPNDDNENIS-SYIPLIFIHIIDRFR  Pmar\_PMAR014276\_Pmar\_239864584      --MGQLP-FLG--------EELDQDDTVLC-GTFPSGEVVFARITKSGQ-------NCCVLFRETPVVSPAF------DGSFYF-VSPHAGDIYD-KAMWDAFRLYLDEAL  cgd7\_410\_Cpar\_126652095             LKERVD---LG--------ANYNLQMLVK-DLTENEE---EFLLFSNGE----------IYFRNLNVP-----------DNHWI-DPKNPNNPYS-HEIWSGFLQYINNLV  GSPATT00035725001\_Ptet\_145496001    LKYKRKE-WNLIINQIKSEKSSQGKQVELPMIQVIQI---KDPFIQEET--------QGIYIQNQN----------------WNPEDEFQEDLYQ-KQEWNIFIEYLQSII  Dpur1000008840\_Dpur\_Dpur1000008840  ILKRMKT-LGY--------PCYSFDLIIL-PIAIS------CSFIIDGE-------KPHRVMYPPS-----------GRFEGI-DPSDPNPDFFL-PHTWKQLEEFLKSSH  GSPATT00006860001\_Ptet\_145497923    SKSQKHS-IKF--------RSSNGKLIELPLLQVKNI---KDPLIQEET--------YGIYIYQQN----------------WIVEDEFQPELDK-KQEWTIFIEFLMDFF  GSPATT00002404001\_Ptet\_145527744    LKNQKQQ-IKF--------RSSNGKLIELPLLQVKNI---KDPLIQEET--------YGIYIYQQN----------------WIVEDEFQPELDK-KQEWTIFIEFLMDFF  DDB\_G0269710\_Ddis\_66825719          ILKRMKT-LGY--------PCYNFDLIVL-PIALS------GSLIIDGE-------KPTRVLYPST-----------GRFEGI-DPSVPNPDFFL-PHTWRQLESFLKSSH  GSPATT00033531001\_Ptet\_145490150    ----------------------------------------SASQQINNE-------SLLLFTIEDN--------------INWKMMDAQQMNSIY-KSQWRSFIQFLKDFF  GSPATT00010196001\_Ptet\_145510865    ----------------------------------------SASQQINNE-------SLLLFTIEDN--------------INWKMMDAQQMSSIY-KSQWRSFIQFLKDFF  TTHERM\_00129230\_Tthe\_89298162       VKINQLY-WNG-VILYIKSLADINGDIIFNKPNILNLKLKKFKDPLNNT-------ESYLMYVKDT--------------IEWIHSDNGPILHED-KDLWRIFIEFIKEFF  TTHERM\_00353270\_Tthe\_146181541      ---------------YI--QNYQNGQNGLDLPQLVVQK---VNDPLMGA-------DTTVIYIKEQ---------------EWIPEDKGRIDFDN-DKMWKLFKEFLDQYF  TGGT1\_016930\_Tgon\_221483909         VVRRLKL-LEP--------STLVTKNALH-FFRILTDT---FIVEKTNH------SRMVVYLKEKPA-----------WFEGWI-DPKSEEDPYP-QPVWRELEQYLTVLS  AT3G62200\_Atha\_15228700             NISDCIQ-YGD--------PKHHGTDVKK-ALESALE---HHMIMMTN------VGKLKLYIGKNEA-----------LWNCVN-PLGANAKQYPKE-TWDRIQQFLTSS-  AT5G09840\_Atha\_15242519             ESVDEVK-VGADD------VDSKDKDASP-GFL-------GRLLK-----------SFK-FWGKNTKSSKDSSG----NQELVN-VDSQVQDIFAKEYFWSDIESFINSP-  AT5G64710\_Atha\_15238228             ESAEEVK-VDN--------AVGNGKSKSP-GLT-------CRLLK-----------RFKFSWGRYTE-----------LSNAAA-TGPQVDDVFVKDSFWNDVESFINSP-  consensus/85%                       ..............................................p.p..........hhh....................h........p.b.....hp.h..hh.p.. |

  
  
  
  
Top  


---

Multiple
Alginment of OHA (OST-HTH Associated domain)  
  

|  |
| --- |
| FINAL                               ---------------HHHHHHHHH-----------HHHHHHHHHHHHH-H--EE-E-----EEE---EEEEHHHHHH---------  TP03\_0796\_Tpar\_71026501             NDKVKDDKDFN--GGRYAFAERLKN-EVEAFRDMRLGEVVHLVQLAIY-SGIFV-YTQR-ILLP---VSACEKTAQELFPKLKKSR  TA18280\_Tann\_85001524               NDKVKDDKDFN--GGRYAFAERLKN-EVEAFKNMRLGEVVHLVQLAIY-SGIFV-YTQR-ILLP---VSACEKTAQELFPKLKKSR  BBOV\_III000420\_Bbov\_156087541       NEASEGLVSFT--GGRYAFAERLRE-EVHAFRSMRLGEVVHLVQLAIY-TGVFV-YAQR-ILLP---VTACEKTAEEMFPRMKKAR  BBOV\_II001020\_Bbov\_156084292        NNPDQTMPVFP--GSIYGTAKVFQKLELPFFEGMTLGTLCHIVQLAVR-VRKYLMYELK-TLKP---NLLSILQAC-----IKRTK  TA08680\_Tann\_84996849               --DEHGNEYFP--GSIYGMSRTMQDHNLPFLEGMSLGTICHIIQLAIR-LKKYLVYDMK-TLKP---SESVRTGQFTPIPEDAEET  TP04\_0484\_Tpar\_71028952             --DEHGNEYFP--GSIYGMSRTMQEHNLPFLEGMSLGTICHIIQLAIR-LKKYLVYDMK-TLKP---SESVRSGQFIPIPEAVEEN  TGGT1\_045580\_Tgon\_221485777         -LLSSAPNGIG--GGRYGLAKYLQQLGLPFFKGFSLGQLCHVVQLAIS-HKFLLAYEDN-VLKP---VTACAVFANALLGLPDAKR  Pmar\_PMAR023053\_Pmar\_239875739      -LYDPSFYTFP--GGRYGTARALKERNLRFLNGYSLGELCHIVQLAID-KEILA-YGKSGSVVP---FKVSSKAEKDANARDKKPT  CMU\_005100\_Cmur\_209557542           QGNTLKTYQFK--GGRYGFAVELYEKALPFLDGYSLGSICHIVQLAIW-KGILR-YENN-ILQP---AAACWDVSSVCTSGFFNKS  CMU\_006480\_Cmur\_209881271           VTPILMRYQFK--GGRYGVAIEIHKANIPELKDLSVGQICHLVQLAIS-KGILQ-YENN-VLQP---RCTCLKIAAAVLALDIEKD  PF14\_0195\_Pfal\_124808633            KSEDATLYTFS--GGRYGMAKELKKRNLPFFQGLFLGQLCHIVHISTN-KKIIA-YENN-YLKP---ISQCHKYTSAKKGIINGDE  BBOV\_III010230\_Bbov\_156089481       KHDVNISHDIRNVGGRYLFADHLRKTGPERFRSLPLGKVVRIVQDALD-SKILS-YDGN-NVVP---VVSSMSTANKIISRMQMMK  Pmar\_PMAR006821\_Pmar\_239903346      QNGRRDLYIFP--KGRYSCAHALEARKLPFFNGLTMGHLINVVQLAMSKYNLLMFDEDK-SIRP---PQAAQKVIRSQQRKPSEDS  PF14\_0367\_Pfal\_124809304            --LQSNDNNHK--GGRYILAETLKHTGPYIFRTMKLGRIIHILQKCID-LNILS-YFNN-NIIP---IFTSMSISKTYVSKLHVNE  cgd7\_410\_Cpar\_126652095             IEPDLLRYQFK--GGRYGVAMEIHKAKISQLEDLSLGELSHLVQLAIN-GGILQ-YENN-VLKP---RCTCVKIAAAALSMDVDQI  GSPATT00035725001\_Ptet\_145496001    ---QNDYQFFK--DGGYGCAQFIKLFGPKNLRELSLARLKLYTHMAIN-KHYIR-NTKK-VQYN---REKSEYATDDSQDRDNNIK  Dpur1000008840\_Dpur\_Dpur1000008840  -------PISR--NGRYGFATFLKESGPNEIREMPLAVITSMVELAFK-KKLLT-YKNC-NVFSN--MEVFVGEINSTSEVSANDG  GSPATT00006860001\_Ptet\_145497923    -TNAQNNKILK--GGRYGCAQFIKLLGPKKLRDLSLGRLTLYVQMAVN-KNYIR-YNKT-ILIQES-REKSEHTTDSSVDGDPQ--  GSPATT00002404001\_Ptet\_145527744    -TNAQNNKILK--GGRYGCAQFIKLLGPKKLRELSLGRLTLYVQMAVN-KNYIR-YNKT-ILIQES-REKSEHTTDSSVDGDPQ--  DDB\_G0269710\_Ddis\_66825719          -------PISR--NGRYGFATFLKESGPNEIREMPLAVITSMVELAFK-KKILT-YKNC-NVSSNIEELMFCESSSSIIDIIPD--  GSPATT00033531001\_Ptet\_145490150    KPPKSIQKAIP--GGKYGCAQLLKCCGPLELRVCSLGVLCLMIQEAIN-RNVLI-YYKT-LLIK--PNTNIAFDFNDFLNIFDDQF  GSPATT00010196001\_Ptet\_145510865    KPPKSIQKAIP--GGKYGCAQLLKCCGPLELRVCSLGVLCLMIQEAIN-RNVLI-YYKTLLIKP---NTNIAFDFNDFLNIFDDQF  TTHERM\_00129230\_Tthe\_89298162       RPPKSINKAIP--GGRYGCAQLLKCCGPLPLRSCSLGKLSLFVQEAIT-RGILV-YYKTLLIKP------TNFDFDEVMDITVLDQ  TTHERM\_00353270\_Tthe\_146181541      TQSQQELRAIP--GGRYGCVQYVKYCGPRELQELSLGRLSLFVQEAIN-TGIIR-YHRTLLVK---------HTYNEENSVLNESS  TGGT1\_016930\_Tgon\_221483909         HELNDPKIVLP----PYSSEETSNVDDSSHCENLPTSSLAMVPFTANL-FRKLR------KLLR---QARQQDQAQCFVAASDGAT  AT3G62200\_Atha\_15228700             ----SGRVEFTATTCRYEAAQVLKKE---CLKEFTLGDILQILNITAT-TKKWITHHQT-GWKP---ITISLAAETTNETATEADP  AT5G09840\_Atha\_15242519             ----RGFAIVSHSRTREVMAKNVQEEGPSCLRLLDESSMLHLVTLLIS-EKKWIEETPS-SSLP---FRIIKGSSPGHRHASNGLS  AT5G64710\_Atha\_15238228             ----RGFVAVSHSRSRETMAKNLKEEGPSSLKPLDVPKMLDLISMLIS-EKKWIQENPS-DALP---FRVTRFTEESSCRSNPRTT  TA11490\_Tann\_84995728               -TIIPVEDIFNI-GGRYVFAEHLKKNGPKEFRNLSLGTIVNVVQKLNE-MGLLV-YRGN-CLVP---VVTSKMAANKFNNAVNKMN  TP02\_0125\_Tpar\_71030104             -SIVPVEDIFNV-GGRYVFAEHLKKNGPKEFRNLSLGTIVKVVQKLNE-MGLLV-YRGN-CLVP---VVTSKSAANKFNNNATNRL  PF14\_0291\_Pfal\_124809004            DTTNM-FDIFN--NDIYEVADILKKKNFPILKDYSLGKIAHIIYLCLY-NGLLL-EENQ-KIIP---ACSSKNIISSIFYIKNKNS  consensus/80%                       ....ph...h...sG+YuhAp.lc.....bhcshsLGpls.hlQbAh....hh..Y..p..l.............p...s...... |

  
  
  
  
Top  


---

Multiple
Alginment of the bacterial domain X  
  

|  |
| --- |
| FINAL                          --------HHHHHHHHHH----HHHHHHHHHHHHHHHHHHH------HHHHHHHHHHHHHHHHHHHHHHH-----EEE-E----------------HHHHHHHHHHHH----HHHHHHHHHH-HHHHHHHHHHHHHHHHHH-----HHHHHHHHHHHHHHHHHHHHHHHHHHHHHHHHHHHHHHHHHHH--HHHHH---  Rsph17025\_3537\_Rsph\_146279554  MTSVDAPWLTARHEVEHLFGQCILRLQAFELLLKLIVAGHHVSGS-PATSQDTRASRIAEVRRKTLGGLVGEMMGSVL-VPA------GQEGQHDACDDDAEFACLVRIAFPVGEFARIETE-HRELVALRNALVHHFLEEQDLRTEAGCLSARQALMTTLDRVTRAHADLQTWAAEMEQARRVMAEYVESPEFHDWIV  NAS141\_16058\_Ssp.\_83954751     -MAPDTAPPLERHEIERLLGQCLLRLQAFELRLKAIVATHRLSGP-ADLLEQLRAQRIDETRRKTMGGLVGDLMGNVL-VPE------GQQGLRDEAEDAPAFSFLMQIALPADEFSRIEAE-HRKLTAKRNALVHHFLEEHDLLSEEGCRVAKRDISGIIERVTRADKKLDVLAADMDQVRKVFAEHLASPDVRDWFV  ebA2464\_Aaro\_56476823          HSVSDDEPQAQQREVQRLLGRCLLRLQQYERLMKAIVAHHDISGP-AHSLEAVRAARIEDTATKTLGTLVGQLFGSYV-VTE---GGDVSENEPDLPADMISFRTRVQLSLSAEDYVRTQAD-LKDLVSLRNALVHHFIDQHDLWTIEGCHTAHDALTTAYTRIDQHFEQLRGWAEHMDQARRLAAEFVQSDVFHDLVV  Tmz1t\_0994\_Tsp.\_217969422      TSPATNALQPLQPDVQRLLGRCLLRLQQYERLMKAIVAHHEISGP-AHSLEAIRAARIEDAATKTLGTLIGQLFGSYV-VTD---GNGGEERDDDLPGDVISFRTRVQLSLSAQDYAKTQAD-LKDLVSLRNTLVHHFIDQHDLWTVDGCRAAQDELGSAYTRIDQHFEQLRGWAEHMDQARRLAAEFVHSDVFHDLVV  Tmz1t\_3165\_Tsp.\_237653824      EPRRSEQFVALQHLVQRKLGRCLIRLQQYERLLKALVAEHDVSGP-AHRLIAIRDSRMEGLSKKTLGHVVGALTENLL-TPDSIASDEDGDNDHSAEENAFVLRARFRVELSAKRHEETVTA-LRALVDLRNELVHHFLEKHDIWSESGCITAQAYLEACYEQVDERYMELQAWAKASVEAREYMASFMQTPEFREFLH  AcavDRAFT\_0259\_Aave\_270491449  PTNDRPSLAELQRAVQHKLGGCIWRLQQYERLLKAMVANTDLAGE-PAQLQVLRDARVASVHRTTLGGLVSLFTGGYL-LAE-DGSSPTAVADDKAPGDKLWFSFQQRMTMSAKRHDAITTE-LKELVDLRNELVHHLLERYDLAQLDRCEAAVAYLDASRATIDRHYQTLRTWAEHMDNARALAASFMNSDAFKDMLI  PSPTO\_0007\_Psyr\_28867250       IPLPSDPLPDLQRTVQRKLGRCILQSQQYERLLKAMVAHGELSGP-PERLQAIRDEKVACADKKTLGTLVGMLTESYLKLPD---LSDEPEQAEPIDRVWVSFR--CQMELTEERYAETKAA-LKELVDLRNELVHHFLQRFDLWGVDGCIAAESYLDESYETIDGHYLTLRDWAKSMDEARQLMVSFMQTQEYRDAVI  IPF\_4677\_Maer\_159026983        MD-KPIDIEVVRTEALRKLGRNIVNFSKIEGILKYLLSVSQLEGL-STSTHNQLVDNYERFRKHTLGRLVQKLHNTVL-VDD-------SQSEAQLDSSELGMSLSFKVPYSDSDFLNAQKQALSDIVAERNKLIHEDLALLDTSSIEDYYKLISLLDEQNPRLLAHLKELGWMLTSFIEGIKDLQEFIKSPDFHQFIH  KT71\_11544\_Clit\_88706107       -----MDLDACRDEVVFKLGRNVLLFQQLERTLKWIVPRLEVSGANAAELKDNFAKRQSSASKQTMGMNTGELF---------------TDAEKDVSSQTWALVSSFAFE------PETREE-IEALVCERNELVHHFMDKVEVESPESWRKASIALDHQKERIAAIDALLRDLAAVLKSGIDTAVQEIHADLLQRD--  Kkor\_1893\_Kkor\_256823108       MT-SLDNKDNLRDEALRKIGRNVVNFQKIEALLKVLVLYSSVDSQ-TRDNEVTHSFNEKLIQNRPLGEVVSKFLKNLI--------DNRVAENEELHSSELQISTRFYLDMDAEDVAKEKIE-LKRLVSERNQLIHKDLASIDFNCVKSCRKLISELDEQNSRVLDKLHQLKDIWETFNQMKNELLTYFQSDEFLAVFI  consensus/85%                  ..............................................................p.sh....................................h.....h.................lssbRp.l.Hc.l.b.D..p.p.h............p......pLp.hh..b.p.b....pbh.ps.h.ph..  Legend is  Aaro : Aromatoleum aromaticum; Aave : Acidovorax avenae; Clit : Congregibacter litoralis; Kkor : Kangiella koreensis; Maer : Microcystis aeruginosa; Mavi : Mycobacterium avium; Psyr : Pseudomonas syringae;   Rsph : Rhodobacter sphaeroides; Ssp. : Sulfitobacter sp.; Tsp. : Thauera sp.; Veis : Verminephrobacter eiseniae |

  
  
  
  
Top  


---

Multiple
Alginment of the enzymatic bacterial domain  
  

|  |
| --- |
| FINAL                                    --HHHHHHHHHH-----------------------HHHHHHHHHHHHHHHHHH---------------EEEE--E-----EEEEE---------EEEEEE--E-------------------------------------------------EEEEEEE--------------------------------------EEEE-------------------H-HHHHH--H--HHHH--H---HHHHHHHHH-------------------HHHHHHHHHHHHHHH---HHHHHHHHHHHHHHHHHHHHHHHH----EEEEE----------EEEEEEEE----------EEEEEEEE---------EEEEEEEE-H-HHHHHHHHEE-----------  GCWU000246\_00092\_Jant\_260654181          -DDQYRRLA-GMALPEPWEF------HD---DELPLSILKEYVRATFARVKRLGG--------------LGF--DRKKE-IAAFNTGLLDRNCEPIFAYF--GRKS--------------------------------------------ESWELLDFCLPGQ----GQYGKPLLKNFS----PLPPRAVFS-RSADELIY-----DLSAGEP-QV----D-WNHVL--V--DKISR-----IPGEVLRLVGLDVPNGAPFIQ----------DLSVVRSCRRELRTNPLTHRRLKSIFEGAIDNVMKRLQYDYRLAVPIYRISR----QTLGLALPLDL-TS--SSRVDLALIC-ERIGSV----GYQGHTIYPL-RWVYRSARVMFRPT-VPWLDP  ALIPUT\_00786\_Aput\_167752535              -DAAINDLAVNLALDERWYY-----DEK---DKLAKPILKNYLSYTFERLQYEDEEEIERSKKEVRKPILKI--LTNED-NAVWNTGLVDNIYDPIYAFF--QKNNGKN-------------------------------------PAVIQPWVFLGFGTAN------SYYQKIITDFP----YKPKRAQYF-DDPRELFY-----DITAQRP-TL----D-WNHFI--K--ENIER-----LPVGFIKKGATDGFQFIED----PAALPKPQREAYYKKLADAIFEDDDWKQFLTTRFSNALDIALSRVAWNYKTAIPVYYVKD----HKMQLLLPLAL-EH--KGTIDVALVCNHKYDKEKEVNNYEGRTIFTM-EMAYNNARLITRPD-SDWLMA  MED193\_18819\_Rsp.\_86136205               VRGVLHDLA-EIALDEDWAY-GD--NHN---PRTPFPILRNYLINTFYKQYRDGQ-------------VAEVA-HGGKS-WAAFNTGLVDDRYDPIFALY--EQNDR---------------------------------------PP--RPWKFHAFCRPNI----GREGQTLARNFN----PLPPAPKYF-DKAEDVIF-----DPDT-AI-QP----R-YDHIV--Y--DSIEKDR---YPSDFLEKHIPSGIEWKD-----PSKMEKPERAAFLREFRHALVADARTDRDIRNRIDDAISLAVKRARWNFKTAIPLYYPKA----NAISLLLPIAL-VD--DDKVDLALVV-TRTAAG----GYSGETVYKL-KWAYDHARLVCRPD-SDWLTP  S7335\_1492\_Syn\_254421342                 -NQKVSELA-AIALPEDWYF-GA--HPP---DDFAYPILKSYLRYTFIRLQHERK--------------VID--SPNRE-FKAFNTGLLDRLLRPIYALL--SPYPT---------------------------------------NA--QVWDLT-FCIPGE----GPAGKKLVAHFN----HLPAAANYL-KDPSKVFY-----HLSAGPP-RV----D-WPHII--K--DNMER-----LPYTFIVKYAPANFTPLD-----TTDLDSAQFHAYKKAFADALDADPIAYRSLVNCLSEALSRTLVKTQVNYKTAVPTYYPTL----NSIDLLLPICL-TE--EGIADCAMVA-RQSDSG----AYIGHTILTL-RQAYNNARLICKLD-EHWLSR  ALIPUT\_00693\_Aput\_167752443              -IEYLAN---NIADPEPWDF-----SDA---TQAKYSILKSYIEHTFRKIKSENK----------------ISFSSDNN-FACFNTGLVTANLESIFALA--ERNNR---------------------------------------PDV-AEKGLSPYVFKAFVR--ESDIQLISKFGD----NIPDIADFF-QKPEDLIF-----NPQC-RV-VP----Q-IDHII--A--DNMDR-----FPAHMQGL----------------------------------------SSDEMRRRLVGAINEAQKKARSNYKIAVPQYY--E----GKIQLLLPLCL-TPG-SPNPDLALAT-HKIGNN----TYTARTCLTL-KMAYNNARLIVKPQ-SSWLKP  HMPREF0762\_01890\_Sexi\_269217191          -KDFLKSLA-AIALPEPWDFNGD--ADA---APRRYTILSNYIRYTFYRLTLEDK--------------IGY--SSDET-FCAFNTGLVDTHYDDIYACF--ERNEK---------------------------------------TEAFQPWSFTSFCTAGT----GRYGKQLVRELN----PLPQPASYL-SRKEDLLF-----DLDR-QI-VC----D-IEHIV--I--DNIHR-----LPLEFLRDELASSKECMAVLADIESAPDAEAEAEAYDSLRTIISTKSRLFSRLSNSVNAAIDIARRQVRWNYKTAVPAYYPRT----NNMNLLLPLNL-TE--DNIPDVALVV-ELQKSG----NYQGQTIITM-VQAYRDARLVCRPY-IDWLSP  \_Psyr\_32419263                           -DEKLDQLA-DLAEPENWEY--Q--FTE---ADGRKPILANYLKHTYKRLAKEEK----------------LELATNGL-NVTFNTGLVTPAQEPIYALF--NQNRNE---------------------------------------GARQPWYFQRFV---------RRGSADLNSFD----QLPEMAHYF-DDPAVLVF-----DHRK-DF-RI----N-VEHII--S--ENKER-----FPKPYCDL----------------------------------------DEYALVSLLEGVIKNARQRVRRNYKTAVPNFYR------GKVQLMLPLCI-SN--PSKADLALVI-EDHN-----SFYRAATCLML-DWAYSNARLLAKPD-KEWLQP  Dole\_1264\_Dole\_158521277                 -ETPFEKLA-HMAKKEDWNF-RSPQFRK---DGQKYPILMNYLNYTFLRIQELGL--------------IAY--SDDGS-KSCFNTGLQTKNEKDIYATFFFNQNAEE---------------------------------------LGKPQWTFYSFA---------DSYSEKLKPFN----PLPEVATYI-SYARDLVF-----ETGY-EI-EV----N-TDHIV--D--HNKDR-----LPANLREE-----------------------------------------RTLSIAAIEGSTRFLKEKIRRNYKVAIPHWYS------GRIQLLLPLNL-TS--DYEADLALVA-DRDDDR---KIYRIRTALSM-DMAYVNARLLCRPD-RDWLNP  SAI\_0625\_Saga\_77405473                   -NAKFTELA-ELAEPENW-------TSS---NSKRNDILYSYITHTFERAFELGD------------DYVII--NEDES-YACFNTGLLTDNGEDIICLF--NTFDS----------------------------------------SDEYHWHLFGFR--------KESNWDFLNNFS----KTPLVPHFF-TNPQDIYF-----DPNK-EL-IK----N-LDHIL--E--DNIDR-----FEGRLQEK----------------------------------------GKQFINALLNNAIELTITKCKRNYRIAVPQYYR------GKITYLLPVTI-DG-----QLMSVAV-ENIN-----GRYRVNTIFTL-EMAYKNARLLMKPE-VDWITS  CLOSTASPAR\_04368\_Casp\_225390613          -NAFLQQLA-ETAMTENWTS-----AN----SEQPCDILRSYICHTIYRLQDEDA-------QADPGAPRKI--DEVDG-KIYFNSGLLNHLFRQIIIVG--TKYEME---------------------------------------KEIPCFGTHKFVLMKNPYPYSESDQEIAQVYDGDTYKLPGIAKFF-DDYRAIIF-----DARL-PI-RL----N-DRHIF--L--DGVERKR---LPKYAEEF----------------------------KACRDNESERNALLARISRDFDSALERAKLLAERNYRLAIPQFWREA----GTIQFLLPIYLGELEEADQPHCALAL-SYDDTGR-VKYYRGETILTL-SMAYNNARLIAKPD-VFWLDT  RSAG\_03008\_Rsp.\_253580784                ------------MVPEKWSF-----SD-----TDDNGILKGYLEHTFKRLYEEQK----------------V--WEKKN-YAIFNTGLFNYYYQPIYAYF--IPNL-V---------------------------------------PDRQPWFLDGFY---------TEYYLLKEGIT----CLPEKACYV-ENPSDLVF-----DTKL-PV-IP----Q-YEHIF--GDEENAAR-----LPKEVRDS----------------------------------------SMK--MQLFDGALKQTKRMLEADYRTAIPQYYN------HSIQLLLPICL-RH--PGKPDLALAC-MKTSD---GSKYLGRTCLTL-RMAYHNARLLARVD-RSWLMT  RUMHYD\_01443\_Bhyd\_225573252              -YGQLDELS-KMALPEPWRF-KKPIYKT---KNEDTPILERYIHIVFRKQSIDFN-----SERDARKAAGYF--HVENE-CACFHTGLYTSRYKGIYACF--DRNHKK---------------------------------------TSMKDWYFRGFC---------DELSPFLKYVQ----PLPQMPSYY-MAQNGAGF-----QPDW-PI-RV----N-VEHIL--GDTENLER-----IPAKIRKA------------------------------------------RNLPLLFETAVEIGRRKTVVEPGLIVPQGYQ------GKMQYLLPIYL-TN--EKKPDLALTL-TVME-----GYYLGNTCLTL-EMAYLNARVISRPI-APWLTD  CLOSCI\_00234\_Csci\_167757903              -YGQLDELS-KLALPEPWRF-KKPIYKT---KNEDTPILERYIHIVFRKQSIDFN-----SERDARKAAGYF--HVENE-CACFHTGLYTSRYKGIYACF--DRNHKK---------------------------------------TSMKDWYFRGFC---------DELSPFLKYVQ----PLPQMPSYY-MAQNGAGF-----QPDW-PI-RV----N-VEHIL--GDTDNLER-----IPAKIRKA------------------------------------------RNLPLLFETAVELGRRKTVVEPGLIVPQGYQ------GKMQYLLPIYL-TN--EKKPDLALTL-TVME-----GYYLGNTCLTL-EMAYLNARVISSPI-APWLTE  HOLDEFILI\_00066\_Hfil\_223982556           -IKYLAELA-NLAEKEIW-------TSP---DGKDYDILLNYLQYTFDKAAKDDL--------------VYF--TPNDD-YAAFNTGLLTENGEDIICMF--NKFTG----------------------------------------SQKFAMHLYGFK--------KESDYEFLNNFA----ETPPVVTYF-DNPEKIYF-----DPNK-KV-VK----N-LDHIL--Q--DHTDR-----FSDELKNK----------------------------------------GKLYISALLSSALDLTLKRCKRNYRIAVPQYYN------DEITYLLPVNL-DG-----HKMALAV-GYVN-----NRYRVNTIFTL-SMAYKNARLLMKPE-ADWLAL  STH615\_Sthe\_51891753                     -ARFLEELA-SMAIPERWNY----SQYQ---SKQQHPILKSYVEKTYERLKQQGR---------------VL--RNESK-LL-FNTGLLNVYFKEIYVLG--EAD-----------------------------------------PEYPQRVINARPVL-------ENDRAVLELFLN----QKPPMATYF-DRITDVIF-----DPDL-EI-NT----D-DIHII--D--DNFDR-----IPPKYRNR----------------------------------------KKSEIFALFQAAIEFARIMARRNYKLVVPQYYM------GQIQFLMPIYL-SGEFSGPPDFALVL-QKM-----GDVYRGNTILTL-DMAYQNARLIAKPD-TTWLSP  LiPA006\_gp21\_BPA006\_157325436            -LIYLAE---NLAEKEDWYY-ENPNAKS---SNQKYGVLFQFIHHTFSKCKDENL--------------LKF----KDN-HCLMNTGLLTQSGEEIFMLF--TKNSR----------------------------------------PNEQEWFFNSFY--------RSSDHDIPQNMR---GSLPEHIDYFASNPQDMYF-----NTKL-NV-LY----N-MEHIV--E--ENFTR-----LPEGIQQL----------------------------------------DKSIIITILNSSTEQMKKRILRNNRLVVPQYYN------KRIMYLAPLRF-GK-----DTLPLAI-EKHI-----DSYRINTILTP-GMAYCNARLIMKPE-SNWLNN  LMOf6854\_2339\_Lmon\_47095979              -LIYLAE---NLAEKEDWYY-ENPNAKS---SNQKYGVLFQFIHHTFSKCKDENL--------------LKF----KDN-HCLMNTGLLTQSGEEIFMLF--TKNSR----------------------------------------PNEQEWFFNSFY--------RSSDHDIPQNMR---GSLPEHIDYFASNPQDMYF-----NTKL-NV-LY----N-MEHIV--E--ENFTR-----LPEGIQQL----------------------------------------DKSIIITILNSSTEQMKKRILRNNRLVVPQYYN------KRIMYLAPLRF-GK-----DTLPLAI-EKHI-----DSYRINTILTP-GMAYCNARLIMKPE-SNWLNN  PCC8801\_1879\_Csp.\_218246706              -PQTLDELD-DLAQEETWEY--K--HTP---TRKPKPILASYFNYTFKRLLEENK---------------VV---EQED-KACFNTGLVTDNQEDIFAFF--EQNRNN---------------------------------------PSQ--WKFKQFL---------KESDYLLRSFN----PLPDRASYF-DDPTNLIY-----DIRL-GHPRI----D-YDHIKNRT--ENRNR-----FPQKYQSM----------------------------------------NDHELQVLLEGAVKLAIKRVMRNYKAAVPQFFWDRNAKVGELQLLLPLCL-SS--PLKADVALVI-NRTE-----YVYSGETILTL-DMAYNNARLLAKPD-TEWLQP  OSTLU\_13781\_Oluc\_145340841               -ATSFASLE-SAVVRETW---------P-------RGMMDAYLDAQFLRSVEQGK------------MVQCFTESGDAA-WATFHTNLLSYDEFPLFAMF--GREFTR--------------------------------IDKSQA-VNGGKWELVGFVDDVTLRD-PSQPWNAIEPIL----HVPPRPTFV-EDPCDCLWIA---DETGDDV-VL----E-IDARF--W--ESLESDAVVALLKEFSTLTARHHAGMS-------------------------------AISRLARLVSYGFHAPVPRFARLQGARGP----------GKVQMLLPFKADSK--SMQTRAAVVV-DIIKSRRGGRMYRAVGVVSL-REAVFTARVIGPLTTSDWLNA  Ot01g02890\_Otau\_116000521                -ATSAVGLE-RVASVEKW---------R-------PGMMEAYLDAQFLRSIEQGK------------MVQCFTERGDAA-WAVFHCNLLSRDEFSLFAVF--SRDFAEEDADFFYADAGSPTQKKITATKAQSHVSLNAGIDDARAMVNGERWELCGFVDDIALRD-PSQPWNAIEPIL----HIPPRATFV-DDELDRLWLA---DNDD-DV-TL----E-IDAKF--W--ASLEDNAAI-FPKEFVDTSMRQQFSMN-------------------------------AVSRLARLVSYGYHVPVLRFARLQGQRGP----------GKIQMLLPFKCDPR--SSGTQGAVVV-DIIKSRRGGRMYRAVGIVTL-HEAVLSARIVGPLS-SHWLSA  RTM1035\_11320\_Rsp.\_149204108             -FALVARQ--TVDGVDAWHF-HQQRFKD-KYANQAYPKLRNYLNYTFKRLVALEQ---------QEPGRFFVE-SADGN-WITFNTGLQNAHGADLMAVF--EKYRPRPDMPE----------------------------------RETPDWVFKGCY--------APGDRNYQGNFG--T-KQPDIAWYS-RDSRDYIF-----DTEY-SL-DR----DVFDHLF--E--RAKER-AG--MPNFSDEV--------------------------------------------VRNYLRGALENLIPKIRRNYKIAIPVFYVEE----QRMQLLLPFSS-AS--NVNEVSCFLV-ERDD---ALRTYRIKTIFDL-DHAYFSARLITRPD-RDWLNP  Tcur\_3869\_Tcur\_269128066                 -EDFFDLLA-RLAEPEEWGG--APSGPAQVARSDDTWVLREYIEQTFERLYRQRR--------------ILT--SPDGA-HSVFNTGLVTSRQEEIYGLF--VPSRD----------------------------------------PDGAPWRLQGWY--------TESERELQTHFP----ELPPAATYA-EEPAELVY-----DWRC-EL-VV----N-AGRLL--ESAENLAA-----LPAPLNAN-----------------------------------------PYQAGLVLEGAVRRAQSRARRDYRAAVPCWDPLS----ERVRLLLPLSL-TS--PDAVDAALMV-GRED---AQEVYRGHRLLAL-DIAYARARQLARPH--DWLTP  BACCAP\_03125\_Bcap\_154499130              -PERVHEI--TKSVMQRVSY-------L---PGIDYRQLNNYLYVLLSRNRVSER-------------------------NRVINTRLVDELGQPVYLLY--VGDF------------------------------------NKQV-DMHQAFLVDSIS----------EAQKIFSGVT-QT-RLPDPLQWS-EDVSDYIW-----DPTM-EV-LSLSDRV-VQHIV--S--ERTGR-----ICGQLATL----------------------------------------EPATLLRNIKEALFTGQQRACRDCTYALPAYSRQH----NSVGMMLPLHVYTND-TELPEAVLLL-SKTV-----HGYSLVTIVTP-KQAYISVRMFRNPE-ETWLKG  PPL\_01760\_Ppal\_281210602                 ------------------------------------------MDNTFKRIVEENK------------LVAYFPEDQELPILIVFNTGLLSRTNEDIFCIL--QSIH-------------------VASLKDREQES------GGRR-CEERQWILKEFLPSSSFQQ-NDTFDKYGNNILPLT-ELPKRAYYF---IDGVQY-ASF-DPEI-PV-EK-DNVD-FAEII--KN-SSQSK-IDR-LPQEYVHC----------------------------------------NEKELQTRFNQGIVAAIIRTRSNPRCAVPQFHRDSDG-LKRIDLLLPLNM-NG--KKDPDCALVI-RYNITENGNKSYIVRGILSK-EDSYINARVIQRID-QKWMEY  CCOA0058\_Ccol\_57504951                   -LNTLQELS-NITQKEFWGN-------G---NNILFSYLFKYFEFIYENKSYPDI--------------ITY--NKDKT-KACFNTGLYSTGVFPIFACF--EKQEN-------------------------------------------GGYIFRKFC---------SNGDRVLDDLE-IP---KSLSDYD-TFKNEIIF-----DSKL-DF-RV----N-HLHLF-----ERKER-----LPEIVKKL----------------------------------------NDRFIGHIINGELKIIKDNYNLQ-KMIIPAAYK------QRVVLYIPLKLQ----EESVDTIVVV-EKEEVK-NEQYYAVRTILNPHDNIYKTARVLSIVE-SEWVKN  RUMTOR\_02209\_Rtor\_153815964              -SNVTEAIY-GRLLFPNWKS---------------MKGLDRYIKIIGARITQLVD---------AGRTEYFV--ANKTK-SAIVNTGMMDLFGNDFLVLY--RYYE-----------------------KYKTYIA------ERVI-ESKQDYLNYGFT--------KEQSMMQIEPIS-----------FF-DEKEEVFN--PK-KEDF-DI-NQ-NC---LIHII--Q--ERRER-----FPESIRSQ----------------------------------------SDSKIAGQIMNALERGIKMQRRDRSFAKASYSGKS----GTISWFMPLHIDAPL-SEDPELVMVV---RRSG---DFYEIKTILSY-DDELKDRITALSLY-SKLW--  HMPREF6123\_1588\_Osin\_227873392           -----------------------------------------------------------------------------------MNTGLLTKYDQEIVALF--AKNERD---------------------------------------GSKQAWYFVGFF--------KENDKQFTSHFS----EIPKMANYY-KNASDLIY-----DSNL-EL-RL----Q-KEHII--D--DNFIR-----FSNVGFDN-----------------------------------------KELISVFLDVAKGKLEKKLKRNFKLALPFYYHNTETKEDKIQLLAPLYFP----SAPVKLALVI-NKVKTE-TSEYYEGITILPV-ELAYMN---------------  RUMOBE\_02640\_Robe\_153812241              ------------------------------------------------------------------------------------------MYYQPVYAYF--IPNLV----------------------------------------PDRQRWFLDGFY---------TEYHLLKSGIV----NLPKRAAYV-TDPAELIF-----DTGL-DI-VP----Q-YEHIF--EETENCQR-----LPETIRGS----------------------------------------VMK--VQLFDGALRQTKRMLEADYRTAIPQYYN------HGIQFLIPVCL-QN--PDRADLALAC---VKTE-DGSKYLGRTCLTL-KMAYHNARLLAKIH-SSWLYP  consensus/85%                            ...............p.W....................h..ah...h.+..................................hpTGLhs...p.lhhhh....................................................a.h..a..........p....h.p.h.......P....ah.p..p.hha.....p.p...h.......p...Hlh.....-p.p+.....hs..h.p.............................................h...hp.uhp..b.b...p.p.hlP...........ph.hhhPl.h.........phslhh............Y...shhs..p.uY.sARhh.......Wl..  Legend is  Aput : Alistipes putredinis; BPA006 : Listeria phage A006; Bcap : Bacteroides capillosus; Bhyd : Blautia hydrogenotrophica; Casp : Clostridium asparagiforme; Ccol : Campylobacter coli; Csci : Clostridium scindens; Csp. : Cyanothece sp.; Dole : Desulfococcus oleovorans; Hcin : Helicobacter cinaedi; Hfil : Holdemania filiformis; Jant : Jonquetella anthropi; Lmon : Listeria monocytogenes; Oluc : Ostreococcus lucimarinus;   Osin : Oribacterium sinus; Otau : Ostreococcus tauri; Ppal : Polysphondylium pallidum; Psyr : Pseudomonas syringae; Robe : Ruminococcus obeum; Rsp. : Roseobacter sp.; Rsp. : Roseovarius sp.; Rsp. : Ruminococcus sp.; Rtor : Ruminococcus torques; Saga : Streptococcus agalactiae; Sexi : Slackia exigua; Sthe : Symbiobacterium thermophilum; Syn : Synechococcus sp.; Tcur : Thermomonospora curvata |

  
  
  
  
Top  


---

  
  
  
  
  
Multiple
Alginment of the inactive SGNH-hydrolase  
The inactive SGNH/GDSL hydrolase
domain of Oskar is aligned with a crystal structure of an active GDSL
hydrolase to show that the S, D and H required for the active site
are missing in Oskar.  
  

|  |
| --- |
| ...E1.............H1.................E2............H2................E3..................H3..............E4.....................H4...........E5...........................................H5.........  1yzf.pdb                        -EEEEEEEHHH-----HHHHHHHHHHHHHH---EEEEEEEEE---HHHHHHHHHHHHH------EEEEE------------HHHHHHHHHHHHHHHHH--E--EEEEE------------HHHHHHHHHH---HHHHH---EEEHHHHHHHHh--------------------------HHHHHHHHHHHHHHHHH-  1yzf\_Efae\_29374819              MRKIVLFGDSIDEAVSPVLVDLVKRDIAAMGLeEVAVINAGMPGDTTEDGLKRLNKEVLIeKPDEVVIFFGANDasldrNITVATFRENLETMIHEIGSEK--VILITPPYADSGRRPERPQTRIKELVKVAQEVGAAHNLPVIDLYKAMTVypgtd------------eflqADGLHFSQVGYELLGALIVREIKG                                  -------GDSL--------------------------------G--------------------------G-ND----------------------------------------------------------------------------------------------------D--H-------------------  \*conserved                      -------\*\*\*---------------------------------\*--------------------------\*-\*--------------------------------------------------------------------------------------------------------\*-------------------  #active                         ---------#--------------------------------------------------------------------------------------------------------------------------------------------------------------------#--#-------------------  osk\_Dmel\_24645205               LLDFPLMGDDFMLYLARMELKCRF-----RRHERVLQSGLCVSGLTINGARNRL-KRVQLPEGTQIIVNIGSVDIMRGK--PLVQIEHDFRLLIKEMHNMRLVPILTNLAPLGNYCHDKVLCDKIYRF-NKFIRSECC-HLKVIDIHSCLINERGVVRFDCFQASPRQVTGSKEPYLFWNKIGRQRVLQVIETSLEY  Dsec\GM23770\_Dsec\_195330556     LLDFPLMGDDFMLYLARMELKCRF-----RRHERVLQSGLCVSGLTINGARNRL-KRVQLPEGTQIIVNIGSVDIMRGR--PLVQIEHDFRLLIKEMHNMRLVPILTNLAPLGNYCHDKVLCDKIYRF-NKFIRSECC-HLKVIDIHSCLVNERGVVRFDCFQTSPRQVTGSKEPYLFWNKIGRQRVLQIIETSLEY  Dsim\GD18580\_Dsim\_195572425     LLDFPLMGDDFMLYLARMELKCRF-----RRHERVLQSGLCVSGLTINGARNRL-KRVQLPEGTQIIVNIGSVDIMRGR--PLVQIEHDFRLLIKEMHNMRLVPILTNLAPLGNYCHDKVLCDKIYRF-NKFIRSECC-HLKVIDIHSCLVNERGVVRFDCFQTSPRQVTGSKEPYLFWNKIGRQRVLQIIETSLEY  Dyak\GE25914\_Dyak\_195499262     LLDFPLMGDDFMLYLARMELKCRF-----RRHERVLQSGLCVSGLTINGARNRL-KRVQLPEGTQIIVNIGSVDIMRGK--PLVQIEHDFRLLIKEMHNMRLVPILTNLAPLANYCHDKVLCDKIHRF-NKFIRSECC-HLKVIDIHSCLINERGVVRFDCFQSSPRQVSGSKEPYLFWNKIGRQRVLQVIETSLEY  Dere\GG13545\_Dere\_194903569     LLDFPLMGDDFMLYLARMELKCRF-----RRHERVLQSGLCASGLTINGARNRL-RRVQLPEGTQIIVNIGSVDIMRGK--PLVQIEHDFRLLIKEMHSMRYVPILTNLAPLANYCHDKVLCDKVHRF-NKFIRSECC-HLKVIDIHSCLINERGVVRFDCFQSSPRQVSGSKEPYLFWNKIGRQRVLQVIETSLEY  Dana\GF17692\_Dana\_194741640     LLDFPLMGDDFFLYLARMELNCRF-----KRYERVLQSGLCVSGQTIIGARNRF-RKVYLPEGTQIIVNIGSVDIMRGK--PLVQIEHDFRLLIKEIHSRRCIPILTNLAPLANYCHDKVLCDKITKF-NRFVRNEARSHLKFIDINSCLINEKKNVLFDCFQSAPRTVTGSKEPYLFWNKIGRQRVLQTIESCLEY  Dvir\osk\_Dvir\_195389208         LLDFPLLGDDFFLYLARMELKCRF-----KKFEKVLQSGLCISGQTINAARQRL-RLVELPEMTQIIVNIGSEDIMRGR--SLVQIEHDFRLLVKEMHNRRFVPVLTTLAPLANCRHDKQTCDKVSRF-NKFIRSEGR-HLKVIDIHSCLINENGIVRFDCFQNGPRSVTGSSEPYVFWNKIGRQRVLHMIEENLEY  osk\_Dvir\_2498716                LLDFPLLGDDFFLYLARMELKCRF-----KKFEKVLQSGLCISGQTINAARQRL-RLVELPEMTQIIVNIGSEDIMRGR--SLVQIEHDFRLLVKEMHNRRFVPVLTTLAPLANCRHDKQTCDKVSRF-NKFIRSEGR-HLKVIDIHSCLINENGIVRFDCFQNGPRSVTGSSEPYVFWNKIGRQRVLHMIEENLEY  Dper\GL21554\_Dper\_195152922     LLDFPLLGDDFLLYLARMELRCRF-----KRTERVLQSGLCVSGQTISGARSRL-HHLLVNKGTQIIVNIGSVDIMRGR--PIVQIQHDFRQLVKDMHNRGLVPILTTLAPLANYCHDKAMCDKVVKF-NQFIWKECASYLKVIDIHSCLVNENGVVRFDCFQYSSRNVTGSKESYVFWNKIGRQRVLQMIEASLEY  Dpse\GA10627\_Dpse\_198454187     LLDFPLLGDDFLLYLARMELRCRF-----KRTERVLQSGLCVSGQTISGARSRL-HHLLVNKGTQIIVNIGSVDIMRGR--PIVQIQHDFRQLVKDMHNRGLVPILTTLAPLANYCHDKAMCDKVVKF-NQFIWKECASYLKVIDIHSCLVNENGVVRFDCFQYSSRNVTGSKESYVFWNKIGRQRVLQMIEASLEY  oskar\_Dimm\_111663086            LLDFPLLGDDFFLYLARMELKCRF-----KKDEKVLQSGLCISGQTINAARQRV-QHVELQEMTQIIVNIGSVDIMRGK--PLVQIEHDFRQLIKEMHNRRFVPVLTTLAPLANYCHDKQTCEKVLRF-NKFIRNEGR-HLTVIDIHTCLINENGVVRFDCFQKGPRSVTGSVEPYVFWNKIGRQRVLQMIEQNLEY  oskar\_Dimm\_111663088            LLDFPLLGDDFFLYLARMELKCRF-----KKDEKVLQSGLCISGQTINAARQRV-QHVELQEMTQIIVNIGSVDIMRGK--PLVQIEHDFRQLIKEMHNRRFVPVLTTLAPLANYCHDKQTCEKVLRF-NKFIRNEGR-HLTVIDIHTCLINENGVVRFDCFQNGPRSVTGSVEPYVFWNKIGRQRVLQMIEQNLEY  Dmoj\GI10055\_Dmoj\_195111098     LLDFPLLGDDFLLYLARMELKCRF-----KKYEKVLQSGLCISGQTINAARQRL-RLVELPEMTQIIVNIGSVDIMRGR--PLVQIEHDFRQLVKEMHSRRFVPVLTTLAPLANYRHDKQTCDKVLRL-NKFIRNEGR-HLKVIDIHSCLINENGVVRFDCFQNGPRSVTGSSEPHVFWNKIGRQRVLQMIEANLEY  Dgri\GH23955\_Dgri\_195054868     LLDFPLLGDDFLLYLARMELKCRL-----KKYEKVLQSGLCISGQTISAARQRL-RHVELAEMTQIIVNIGSVDIMRGR--PLVQIEHDFRQLIKEMHNRRFVPVLTTLAPLANYRHDKHTCDKVSRF-NKFIRSEGR-HLKVIDIHSCLVNENGIVRFDCFQNAPRSVTGSSEPHVFWNKIGRQRVLQMIEENVEY  Dwil\GK11117\_Dwil\_195445335     LLDFPLLGDDFLLYLARMELKCRF-----KKYSKVLQSGLCISGQTINAARQNV-CHVELPEQTQIIVNIGSVDIMRGR--PLVQIEHDFRQLIKEMHNRRFIPVITTLAPLANYRHDQATCEKINRF-NKFIRKECR-HLKVIDIHSCLINDKGIVLFDCYQNGPRAVTGSKEPYVFWNKIGRQRVLQMIEANLEY  AaeL\_AAEL000442\_Aaeg\_157134733  ----QMIGDDFFLSLARWELGFQY-----DPGHNIQQSGLCISGQTIAEAADRV-MKAPYIN-DRVIINLGVVDILHGN--DFVDMQEDLYQLKKNLENRGARAILTTLSPLANSSHIPDISDKLHRF-NNLIRRYQ---WEHIDLWRCFVNERDQTLYECFQPAPRQVTGSNKPHVLWNKLGRQRIIKFMKTQLAS  CpipJ\_CPIJ007471\_Cqui\_170041806 ----QMIGDDFFLSLARWELGYSF-----DPGHTIQQSGLCISGLTIAEAVDRV-TKATVLN-DRVILHIGVVDLLHGH--EYVDMQMDLLRLMRVFEERGVRVILTTLSPIANSSHMPGVVNRYSQF-NSLIRNSN---WRYIDLYRCFVNERHNTLYECYQPGPRHVSGSNQPHVLWNKLGRQRIIKFLKTQLAR  AgaP\_AGAP003545\_Agam\_118783859  ----QLVGDDFFLAIAKWELGFSF-----DPGHDIDMSGLCISGLTLSEAAKRV-EMAPFIA-DHVLVNVGTVDLLHGR--AMIDLIHDFNQLVARFRERNVEPIMTTLTPIANSGGRTTMAERLLKL-NEYICRTC---PRTIDLWKHFVHADGTVRFECFQPGPRKVSGSIMPHVLWNKLGRQHMLGVLGNEIAA      Legend is  Aaeg : Aedes aegypti; Agam : Anopheles gambiae; Cqui : Culex quinquefasciatus; Dana : Drosophila ananassae; Dere : Drosophila erecta; Dgri : Drosophila grimshawi; Dimm : Drosophila immigrans; Dmel : Drosophila melanogaster;   Dmoj : Drosophila mojavensis; Dper : Drosophila persimilis; Dpse : Drosophila pseudoobscura; Dsec : Drosophila sechellia; Dsim : Drosophila simulans; Dvir : Drosophila virilis; Dwil : Drosophila willistoni; Dyak : Drosophila yakuba |

  
  
  
  
Top  
  


---

  
  
Family
clusters of OST-HTH domain containing proteins with domain
architectures   
  

```
# 52;  
119574308         LK-Nuclease+RRM+RRM+OST-HTH+OST-HTH+OST-HTH                                       LKAP                   1828   Homo sapiens                             metazoa>vertebrata                   limkain b1, isoform CRA_d [Homo sapiens].  
149631838         LK-Nuclease+RRM+RRM+OST-HTH+OST-HTH+OST-HTH                                       LOC100087164           1744   Ornithorhynchus anatinus                 metazoa>vertebrata                   PREDICTED: similar to limkain b1 [Ornithorhynchus anatinus].  
224070088         LK-Nuclease+RRM+RRM+OST-HTH+OST-HTH+OST-HTH+OST-HTH                               LOC100220674           1744   Taeniopygia guttata                      metazoa>vertebrata                   PREDICTED: KIAA0430 [Taeniopygia guttata].  
149725909         LK-Nuclease+RRM+RRM+OST-HTH+OST-HTH+OST-HTH                                       LOC100050501           1743   Equus caballus                           metazoa>vertebrata                   PREDICTED: similar to limkain b1 [Equus caballus].  
281340646         LK-Nuclease+RRM+RRM+OST-HTH+OST-HTH+OST-HTH+OST-HTH                               PANDA_016760           1743   Ailuropoda melanoleuca                   metazoa>vertebrata                   hypothetical protein PANDA_016760 [Ailuropoda melanoleuca].  
85797660          LK-Nuclease+RRM+RRM+OST-HTH+OST-HTH+OST-HTH+OST-HTH                               KIAA0430               1742   Homo sapiens                             metazoa>vertebrata                   limkain b1 [Homo sapiens].  
109127677         LK-Nuclease+RRM+RRM+OST-HTH+OST-HTH+OST-HTH+OST-HTH                               LOC713831              1742   Macaca mulatta                           metazoa>vertebrata                   PREDICTED: similar to limkain b1 isoform 2 [Macaca mulatta].  
114661168         LK-Nuclease+RRM+RRM+OST-HTH+OST-HTH+OST-HTH+OST-HTH                               KIAA0430               1742   Pan troglodytes                          metazoa>vertebrata                   PREDICTED: limkain b1 isoform 2 [Pan troglodytes].  
219841880         LK-Nuclease+RRM+RRM+OST-HTH+OST-HTH+OST-HTH+OST-HTH                               KIAA0430               1742   Homo sapiens                             metazoa>vertebrata                   KIAA0430 protein [Homo sapiens].  
118098046         LK-Nuclease+RRM+RRM+OST-HTH+OST-HTH+OST-HTH+OST-HTH+OST-HTH                       KIAA0430               1741   Gallus gallus                            metazoa>vertebrata                   PREDICTED: similar to limkain b1 [Gallus gallus].  
119916778         LK-Nuclease+RRM+RRM+OST-HTH+OST-HTH+OST-HTH+OST-HTH                               LOC506615              1741   Bos taurus                               metazoa>vertebrata                   PREDICTED: similar to limkain b1 [Bos taurus].  
73958857          LK-Nuclease+RRM+RRM+OST-HTH+OST-HTH+OST-HTH                                       LOC479837              1739   Canis lupus familiaris                   metazoa>vertebrata                   PREDICTED: similar to limkain b1 isoform 1 isoform 2 [Canis  
219841842         LK-Nuclease+RRM+RRM+OST-HTH+OST-HTH+OST-HTH+OST-HTH                               KIAA0430               1739   Homo sapiens                             metazoa>vertebrata                   KIAA0430 protein [Homo sapiens].  
124487213         LK-Nuclease+RRM+RRM+OST-HTH+OST-HTH+OST-HTH+OST-HTH                               4921513D23Rik          1736   Mus musculus                             metazoa>vertebrata                   limkain b1 [Mus musculus].  
149267750         LK-Nuclease+RRM+RRM+OST-HTH+OST-HTH+OST-HTH+OST-HTH                               LOC100045622           1736   Mus musculus                             metazoa>vertebrata                   PREDICTED: hypothetical protein [Mus musculus].  
19173794          LK-Nuclease+RRM+RRM+OST-HTH+OST-HTH+OST-HTH                                       Lkap                   1735   Rattus norvegicus                        metazoa>vertebrata                   limkain b1 [Rattus norvegicus].  
73958855          LK-Nuclease+RRM+RRM+OST-HTH+OST-HTH+OST-HTH                                       LOC479837              1735   Canis lupus familiaris                   metazoa>vertebrata                   PREDICTED: similar to limkain b1 isoform 1 isoform 1 [Canis  
109489755         LK-Nuclease+RRM+RRM+OST-HTH+OST-HTH+OST-HTH                                       LOC678813              1735   Rattus norvegicus                        metazoa>vertebrata                   PREDICTED: similar to limkain b1 [Rattus norvegicus].  
119574305         LK-Nuclease+RRM+RRM+OST-HTH+OST-HTH+OST-HTH+OST-HTH                               LKAP                   1733   Homo sapiens                             metazoa>vertebrata                   limkain b1, isoform CRA_a [Homo sapiens].  
125951031         LK-Nuclease+RRM+RRM+OST-HTH+OST-HTH+OST-HTH+OST-HTH                               Lkap                   1730   Mus musculus                             metazoa>vertebrata                   RecName: Full=Limkain-b1.  
126334867         LK-Nuclease+RRM+OST-HTH+OST-HTH+OST-HTH                                           LOC100023425           1682   Monodelphis domestica                    metazoa>vertebrata                   PREDICTED: similar to limkain b1 [Monodelphis domestica].  
186910212         LK-Nuclease+RRM+RRM+OST-HTH+OST-HTH+OST-HTH+OST-HTH                               LOC733745              1681   Xenopus (Silurana) tropicalis            metazoa>vertebrata                   limkain b1 (lkap) [Xenopus (Silurana) tropicalis].  
119574307         LK-Nuclease+RRM+RRM+OST-HTH+OST-HTH+OST-HTH                                       LKAP                   1608   Homo sapiens                             metazoa>vertebrata                   limkain b1, isoform CRA_c [Homo sapiens].  
114661176         LK-Nuclease+RRM+OST-HTH+OST-HTH+OST-HTH+OST-HTH                                   KIAA0430               1576   Pan troglodytes                          metazoa>vertebrata                   PREDICTED: limkain b1 isoform 1 [Pan troglodytes].  
193613364         LK-Nuclease+RRM+RRM+OST-HTH+OST-HTH+OST-HTH+OST-HTH                               LOC100169075           1527   Acyrthosiphon pisum                      metazoa>hexapoda                     PREDICTED: similar to limkain b1 [Acyrthosiphon pisum].  
242013710         LK-Nuclease+RRM+OST-HTH+OST-HTH+OST-HTH                                           Phum_PHUM328160        1519   Pediculus humanus corporis               metazoa>hexapoda                     conserved hypothetical protein [Pediculus humanus corporis].  
71891770          LK-Nuclease+RRM+RRM+OST-HTH+OST-HTH+OST-HTH+OST-HTH                               KIAA0430               1506   Homo sapiens                             metazoa>vertebrata                   KIAA0430 protein [Homo sapiens].  
156552882         LK-Nuclease+RRM+RRM+OST-HTH+OST-HTH+OST-HTH                                       LOC100116303           1443   Nasonia vitripennis                      metazoa>hexapoda                     PREDICTED: similar to limkain b1 [Nasonia vitripennis].  
195434471         RRM+OST-HTH                                                                       Dwil\GK15335           1406   Drosophila willistoni                    metazoa>hexapoda                     GK15335 [Drosophila willistoni].  
148664972         LK-Nuclease+RRM+RRM+OST-HTH+OST-HTH+OST-HTH+OST-HTH                               mCG_129812             1373   Mus musculus                             metazoa>vertebrata                   mCG129812, isoform CRA_b [Mus musculus].  
195116126         RRM+OST-HTH                                                                       Dmoj\GI11813           1370   Drosophila mojavensis                    metazoa>hexapoda                     GI11813 [Drosophila mojavensis].  
141795160         LK-Nuclease+RRM+RRM+OST-HTH+OST-HTH+OST-HTH+OST-HTH+OST-HTH                       LOC100005105           1361   Danio rerio                              metazoa>vertebrata>actinopterygii    LOC100005105 protein [Danio rerio].  
195051398         RRM+OST-HTH                                                                       Dgri\GH13633           1341   Drosophila grimshawi                     metazoa>hexapoda                     GH13633 [Drosophila grimshawi].  
195385148         RRM+OST-HTH                                                                       Dvir\GJ13178           1340   Drosophila virilis                       metazoa>hexapoda                     GJ13178 [Drosophila virilis].  
119574306         LK-Nuclease+RRM+RRM+OST-HTH+OST-HTH+OST-HTH                                       LKAP                   1335   Homo sapiens                             metazoa>vertebrata                   limkain b1, isoform CRA_b [Homo sapiens].  
45551016          RRM+OST-HTH                                                                       CG17018                1305   Drosophila melanogaster                  metazoa>hexapoda                     CG17018, isoform D [Drosophila melanogaster].  
45551015          RRM+OST-HTH                                                                       CG17018                1294   Drosophila melanogaster                  metazoa>hexapoda                     CG17018, isoform C [Drosophila melanogaster].  
157121039         RRM+OST-HTH+OST-HTH                                                               AaeL_AAEL001666        1292   Aedes aegypti                            metazoa>hexapoda                     hypothetical protein AaeL_AAEL001666 [Aedes aegypti].  
195476454         RRM+OST-HTH                                                                       Dyak\GE14372           1292   Drosophila yakuba                        metazoa>hexapoda                     GE14372 [Drosophila yakuba].  
189240101         LK-Nuclease+RRM+RRM+OST-HTH+OST-HTH+OST-HTH                                       LOC661475              1280   Tribolium castaneum                      metazoa>hexapoda                     PREDICTED: similar to limkain b1 [Tribolium castaneum].  
198473463         RRM+OST-HTH                                                                       Dpse\GA14273           1280   Drosophila pseudoobscura pseudoobscura   metazoa>hexapoda                     GA14273 [Drosophila pseudoobscura pseudoobscura].  
194877707         RRM+OST-HTH                                                                       Dere\GG21372           1273   Drosophila erecta                        metazoa>hexapoda                     GG21372 [Drosophila erecta].  
170029488         RRM+OST-HTH+OST-HTH                                                               CpipJ_CPIJ000954       1244   Culex quinquefasciatus                   metazoa>hexapoda                     conserved hypothetical protein [Culex quinquefasciatus].  
270011708         LK-Nuclease+RRM+RRM+OST-HTH+OST-HTH+OST-HTH                                       TcasGA2_TC005776       1216   Tribolium castaneum                      metazoa>hexapoda                     hypothetical protein TcasGA2_TC005776 [Tribolium castaneum].  
1930141           LK-Nuclease+RRM+RRM+OST-HTH+OST-HTH+OST-HTH                                       A-362G6.1              1199   Homo sapiens                             metazoa>vertebrata                   Unknown gene product [Homo sapiens].  
110767193         RRM+OST-HTH+OST-HTH                                                               LOC409429              1170   Apis mellifera                           metazoa>hexapoda                     PREDICTED: similar to limkain b1 [Apis mellifera].  
195580777         RRM+OST-HTH                                                                       Dsim\GD24356           1087   Drosophila simulans                      metazoa>hexapoda                     GD24356 [Drosophila simulans].  
195164514         OST-HTH                                                                           Dper\GL21146           1083   Drosophila persimilis                    metazoa>hexapoda                     GL21146 [Drosophila persimilis].  
189541342         LK-Nuclease+RRM+RRM+OST-HTH                                                       LOC100005105           1073   Danio rerio                              metazoa>vertebrata>actinopterygii    PREDICTED: similar to Limkain-b1, partial [Danio rerio].  
158297449         RRM+OST-HTH+OST-HTH                                                               AgaP_AGAP007823        1053   Anopheles gambiae str. PEST              metazoa>hexapoda                     AGAP007823-PA [Anopheles gambiae str. PEST].  
195358583         RRM+OST-HTH                                                                       Dsec\GM26675           829    Drosophila sechellia                     metazoa>hexapoda                     GM26675 [Drosophila sechellia].  
194767522         RRM+OST-HTH+OST-HTH                                                               Dana\GF15930           680    Drosophila ananassae                     metazoa>hexapoda                     GF15930 [Drosophila ananassae].  
# 16;  
148905892         LK-Nuclease+OST-HTH+OST-HTH                                                       -                      593    Picea sitchensis                         viridiplantae                        unknown [Picea sitchensis].  
147795296         LK-Nuclease+OST-HTH+OST-HTH                                                       VITISV_001778          531    Vitis vinifera                           viridiplantae                        hypothetical protein [Vitis vinifera].  
195615754         LK-Nuclease+OST-HTH+OST-HTH                                                       -                      531    Zea mays                                 viridiplantae                        hypothetical protein [Zea mays].  
224123372         LK-Nuclease+OST-HTH+OST-HTH                                                       POPTRDRAFT_671445      531    Populus trichocarpa                      viridiplantae                        predicted protein [Populus trichocarpa].  
226528190         LK-Nuclease+OST-HTH+OST-HTH                                                       LOC100275707           531    Zea mays                                 viridiplantae                        hypothetical protein LOC100275707 [Zea mays].  
255555429         LK-Nuclease+OST-HTH+OST-HTH                                                       RCOM_0813110           531    Ricinus communis                         viridiplantae                        conserved hypothetical protein [Ricinus communis].  
115475405         LK-Nuclease+OST-HTH+OST-HTH                                                       Os08g0230500           530    Oryza sativa Japonica Group              viridiplantae                        Os08g0230500 [Oryza sativa (japonica cultivar-group)].  
4335722           LK-Nuclease+OST-HTH                                                               At2g15560              524    Arabidopsis thaliana                     viridiplantae                        unknown protein [Arabidopsis thaliana].  
218200714         LK-Nuclease+OST-HTH+OST-HTH                                                       OsI_28326              523    Oryza sativa Indica Group                viridiplantae                        hypothetical protein OsI_28326 [Oryza sativa Indica Group].  
224103613         LK-Nuclease+OST-HTH+OST-HTH                                                       POPTRDRAFT_804270      504    Populus trichocarpa                      viridiplantae                        predicted protein [Populus trichocarpa].  
30679459          LK-Nuclease+OST-HTH+OST-HTH                                                       AT2G15560              489    Arabidopsis thaliana                     viridiplantae                        unknown protein [Arabidopsis thaliana].  
168020521         LK-Nuclease+OST-HTH+OST-HTH                                                       PHYPADRAFT_125967      474    Physcomitrella patens subsp. patens      viridiplantae                        predicted protein [Physcomitrella patens subsp. patens].  
167999628         LK-Nuclease+OST-HTH+OST-HTH                                                       PHYPADRAFT_112426      465    Physcomitrella patens subsp. patens      viridiplantae                        predicted protein [Physcomitrella patens subsp. patens].  
242085250         LK-Nuclease+OST-HTH                                                               SORBIDRAFT_08g006900   457    Sorghum bicolor                          viridiplantae                        hypothetical protein SORBIDRAFT_08g006900 [Sorghum bicolor].  
167997905         LK-Nuclease+OST-HTH                                                               PHYPADRAFT_173873      449    Physcomitrella patens subsp. patens      viridiplantae                        predicted protein [Physcomitrella patens subsp. patens].  
168032729         LK-Nuclease+OST-HTH                                                               PHYPADRAFT_134054      431    Physcomitrella patens subsp. patens      viridiplantae                        predicted protein [Physcomitrella patens subsp. patens].  
# 15;  
125578595         LK-Nuclease+OST-HTH+OST-HTH                                                       OsJ_35318              1005   Oryza sativa Japonica Group              viridiplantae                        hypothetical protein OsJ_35318 [Oryza sativa Japonica Group].  
115487478         LK-Nuclease+OST-HTH+OST-HTH                                                       Os12g0162900           1004   Oryza sativa Japonica Group              viridiplantae                        Os12g0162900 [Oryza sativa (japonica cultivar-group)].  
218186477         LK-Nuclease+OST-HTH+OST-HTH                                                       OsI_37572              1004   Oryza sativa Indica Group                viridiplantae                        hypothetical protein OsI_37572 [Oryza sativa Indica Group].  
224126929         LK-Nuclease+OST-HTH+OST-HTH                                                       POPTRDRAFT_580004      998    Populus trichocarpa                      viridiplantae                        predicted protein [Populus trichocarpa].  
225431392         LK-Nuclease+OST-HTH+OST-HTH                                                       LOC100259153           990    Vitis vinifera                           viridiplantae                        PREDICTED: hypothetical protein [Vitis vinifera].  
255578428         LK-Nuclease+OST-HTH+OST-HTH                                                       RCOM_0009810           952    Ricinus communis                         viridiplantae                        conserved hypothetical protein [Ricinus communis].  
115484353         LK-Nuclease+OST-HTH                                                               Os11g0167300           925    Oryza sativa Japonica Group              viridiplantae                        Os11g0167300 [Oryza sativa (japonica cultivar-group)].  
218185316         LK-Nuclease+OST-HTH                                                               OsI_35252              925    Oryza sativa Indica Group                viridiplantae                        hypothetical protein OsI_35252 [Oryza sativa Indica Group].  
222615583         LK-Nuclease+OST-HTH                                                               OsJ_33097              925    Oryza sativa Japonica Group              viridiplantae                        hypothetical protein OsJ_33097 [Oryza sativa Japonica Group].  
15242519          LK-Nuclease+OST-HTH+OST-HTH                                                       AT5G09840              924    Arabidopsis thaliana                     viridiplantae                        unknown protein [Arabidopsis thaliana].  
270255240         LK-Nuclease+OST-HTH+OST-HTH                                                       GSVIVT01026616001      871    Vitis vinifera                           viridiplantae                        unnamed protein product [Vitis vinifera].  
224056045         LK-Nuclease+OST-HTH+OST-HTH                                                       POPTRDRAFT_840796      855    Populus trichocarpa                      viridiplantae                        predicted protein [Populus trichocarpa].  
15238228          LK-Nuclease+OST-HTH+OST-HTH                                                       AT5G64710              841    Arabidopsis thaliana                     viridiplantae                        unknown protein [Arabidopsis thaliana].  
19699357          LK-Nuclease+OST-HTH+OST-HTH                                                       -                      841    Arabidopsis thaliana                     viridiplantae                        AT5g64710/MVP7_3 [Arabidopsis thaliana].  
42573794          LK-Nuclease+OST-HTH+OST-HTH                                                       AT5G64710              716    Arabidopsis thaliana                     viridiplantae                        unknown protein [Arabidopsis thaliana].  
#  
47211982          LK-Nuclease+RRM+RRM+OST-HTH+OST-HTH+OST-HTH+OST-HTH                               GSTEN:00011844:G:001   1130   Tetraodon nigroviridis                   metazoa>vertebrata>actinopterygii    unnamed protein product [Tetraodon nigroviridis].  
172087294         LK-Nuclease+RRM+OST-HTH                                                           BACOIKO008_33          1190   Oikopleura dioica                        metazoa                              limkain b1-like protein [Oikopleura dioica].  
168016647         LK-Nuclease+OST-HTH+OST-HTH                                                       PHYPADRAFT_162624      812    Physcomitrella patens subsp. patens      viridiplantae                        predicted protein [Physcomitrella patens subsp. patens].  
255578272         LK-Nuclease+OST-HTH                                                               RCOM_0537650           230    Ricinus communis                         viridiplantae                        hypothetical protein RCOM_0537650 [Ricinus communis].  
225428830         LK-Nuclease+OST-HTH+OST-HTH                                                       LOC100265894           1361   Vitis vinifera                           viridiplantae                        PREDICTED: hypothetical protein [Vitis vinifera].  
270246019         LK-Nuclease+OST-HTH+OST-HTH                                                       GSVIVT01031726001      437    Vitis vinifera                           viridiplantae                        unnamed protein product [Vitis vinifera].  
242082900         LK-Nuclease+OST-HTH+OST-HTH+OST-HTH                                               SORBIDRAFT_08g004010   997    Sorghum bicolor                          viridiplantae                        hypothetical protein SORBIDRAFT_08g004010 [Sorghum bicolor].  
  
# 27;  
123123576         OST-HTH+OST-HTH+TUDOR+Thermonuclease+TUDOR+Thermonuclease+TUDOR+Thermonuclease    Tdrd7                  1119   Mus musculus                             metazoa>vertebrata                   tudor domain containing 7 [Mus musculus].  
73971398          OST-HTH+OST-HTH+TUDOR+Thermonuclease+TUDOR+Thermonuclease+TUDOR+Thermonuclease    LOC474770              1115   Canis lupus familiaris                   metazoa>vertebrata                   PREDICTED: similar to tudor domain containing 7 isoform 2 [Canis  
20302032          OST-HTH+OST-HTH+TUDOR+Thermonuclease+TUDOR+Thermonuclease+TUDOR+Thermonuclease    Tdrd7                  1113   Rattus norvegicus                        metazoa>vertebrata                   tudor domain containing 7 [Rattus norvegicus].  
281341268         OST-HTH+OST-HTH+TUDOR+Thermonuclease+TUDOR+Thermonuclease+TUDOR+Thermonuclease    PANDA_002927           1101   Ailuropoda melanoleuca                   metazoa>vertebrata                   hypothetical protein PANDA_002927 [Ailuropoda melanoleuca].  
47212266          OST-HTH+OST-HTH+TUDOR+Thermonuclease+TUDOR+TUDOR+Thermonuclease                   GSTEN:00013501:G:001   1100   Tetraodon nigroviridis                   metazoa>vertebrata>actinopterygii    unnamed protein product [Tetraodon nigroviridis].  
20381088          OST-HTH+OST-HTH+TUDOR+Thermonuclease+TUDOR+Thermonuclease+TUDOR+Thermonuclease    TDRD7                  1098   Homo sapiens                             metazoa>vertebrata                   Tudor domain containing 7 [Homo sapiens].  
55631945          OST-HTH+OST-HTH+TUDOR+Thermonuclease+TUDOR+Thermonuclease+TUDOR+Thermonuclease    TDRD7                  1098   Pan troglodytes                          metazoa>vertebrata                   PREDICTED: tudor domain containing 7 isoform 2 [Pan troglodytes].  
73971396          OST-HTH+OST-HTH+TUDOR+Thermonuclease+TUDOR+Thermonuclease+TUDOR+Thermonuclease    LOC474770              1098   Canis lupus familiaris                   metazoa>vertebrata                   PREDICTED: similar to tudor domain containing 7 isoform 1 [Canis  
109110889         OST-HTH+OST-HTH+TUDOR+Thermonuclease+TUDOR+Thermonuclease+TUDOR+Thermonuclease    PCTAIRE2BP             1098   Macaca mulatta                           metazoa>vertebrata                   PREDICTED: similar to tudor domain containing 7 [Macaca mulatta].  
112293287         OST-HTH+OST-HTH+TUDOR+Thermonuclease+TUDOR+Thermonuclease+TUDOR+Thermonuclease    TDRD7                  1098   Homo sapiens                             metazoa>vertebrata                   tudor domain containing 7 [Homo sapiens].  
119579248         OST-HTH+OST-HTH+TUDOR+Thermonuclease+TUDOR+Thermonuclease+TUDOR+Thermonuclease    TDRD7                  1098   Homo sapiens                             metazoa>vertebrata                   tudor domain containing 7, isoform CRA_b [Homo sapiens].  
123994891         OST-HTH+OST-HTH+TUDOR+Thermonuclease+TUDOR+Thermonuclease+TUDOR+Thermonuclease    TDRD7                  1098   synthetic construct                                                           tudor domain containing 7 [synthetic construct].  
154152201         OST-HTH+OST-HTH+TUDOR+Thermonuclease+TUDOR+Thermonuclease+TUDOR+Thermonuclease    TDRD7                  1098   Bos taurus                               metazoa>vertebrata                   tudor domain containing 7 [Bos taurus].  
194034061         OST-HTH+OST-HTH+TUDOR+Thermonuclease+TUDOR+Thermonuclease+TUDOR+Thermonuclease    LOC100152652           1098   Sus scrofa                               metazoa>vertebrata                   PREDICTED: similar to LOC506702 protein [Sus scrofa].  
197102106         OST-HTH+OST-HTH+TUDOR+Thermonuclease+TUDOR+Thermonuclease+TUDOR+Thermonuclease    TDRD7                  1098   Pongo abelii                             metazoa>vertebrata                   tudor domain containing 7 [Pongo abelii].  
194225516         OST-HTH+OST-HTH+TUDOR+Thermonuclease+TUDOR+Thermonuclease+TUDOR+Thermonuclease    LOC100064733           1097   Equus caballus                           metazoa>vertebrata                   PREDICTED: similar to LOC506702 protein [Equus caballus].  
126333960         OST-HTH+OST-HTH+TUDOR+Thermonuclease+TUDOR+Thermonuclease+TUDOR+Thermonuclease    LOC100014171           1092   Monodelphis domestica                    metazoa>vertebrata                   PREDICTED: similar to tudor domain containing 7, [Monodelphis  
189521661         OST-HTH+OST-HTH+TUDOR+Thermonuclease+TUDOR+Thermonuclease+TUDOR+Thermonuclease    LOC100004703           1092   Danio rerio                              metazoa>vertebrata>actinopterygii    PREDICTED: similar to tudor domain containing 7 [Danio rerio].  
73971400          OST-HTH+OST-HTH+TUDOR+Thermonuclease+TUDOR+Thermonuclease+TUDOR+Thermonuclease    LOC474770              1090   Canis lupus familiaris                   metazoa>vertebrata                   PREDICTED: similar to tudor domain containing 7 isoform 3 [Canis  
73971402          OST-HTH+OST-HTH+TUDOR+Thermonuclease+TUDOR+Thermonuclease+TUDOR+Thermonuclease    LOC474770              1090   Canis lupus familiaris                   metazoa>vertebrata                   PREDICTED: similar to tudor domain containing 7 isoform 4 [Canis  
58475742          OST-HTH+OST-HTH+TUDOR+Thermonuclease+TUDOR+Thermonuclease+TUDOR+Thermonuclease    Tdrd7                  1086   Rattus norvegicus                        metazoa>vertebrata                   Tdrd7 protein [Rattus norvegicus].  
148227186         OST-HTH+OST-HTH+TUDOR+Thermonuclease+TUDOR+Thermonuclease+TUDOR+Thermonuclease    MGC81371               1079   Xenopus laevis                           metazoa>vertebrata                   hypothetical protein LOC414521 [Xenopus laevis].  
149383922         OST-HTH+OST-HTH+TUDOR+Thermonuclease+TUDOR+TUDOR+Thermonuclease                   -                      1079   Danio rerio                              metazoa>vertebrata>actinopterygii    tudor domain containing protein 7 [Danio rerio].  
150170656         OST-HTH+OST-HTH+TUDOR+Thermonuclease+TUDOR+TUDOR+Thermonuclease                   tdrd7                  1079   Danio rerio                              metazoa>vertebrata>actinopterygii    tudor domain containing 7 isoform 1 [Danio rerio].  
58332562          OST-HTH+OST-HTH+TUDOR+Thermonuclease+TUDOR+Thermonuclease+TUDOR+Thermonuclease    LOC496822              1077   Xenopus (Silurana) tropicalis            metazoa>vertebrata                   hypothetical protein LOC496822 [Xenopus (Silurana) tropicalis].  
224089725         OST-HTH+TUDOR+Thermonuclease+TUDOR+Thermonuclease+TUDOR+Thermonuclease            LOC100221690           1054   Taeniopygia guttata                      metazoa>vertebrata                   PREDICTED: tudor domain containing 7 [Taeniopygia guttata].  
148670437         OST-HTH+TUDOR+Thermonuclease+TUDOR+Thermonuclease+TUDOR+Thermonuclease            Tdrd7                  959    Mus musculus                             metazoa>vertebrata                   tudor domain containing 7 [Mus musculus].  
# 21;  
114568108         OST-HTH+OST-HTH+OST-HTH+TUDOR+Thermonuclease                                      TDRD5                  1205   Pan troglodytes                          metazoa>vertebrata                   PREDICTED: tudor domain containing 5 [Pan troglodytes].  
197927152         OST-HTH+OST-HTH+OST-HTH+TUDOR+Thermonuclease                                      Tdrd5                  1046   Rattus norvegicus                        metazoa>vertebrata                   tudor domain containing 5 isoform a [Rattus norvegicus].  
134035042         OST-HTH+OST-HTH+OST-HTH+TUDOR+Thermonuclease                                      Tdrd5                  1040   Mus musculus                             metazoa>vertebrata                   RecName: Full=Tudor domain-containing protein 5.  
197927156         OST-HTH+OST-HTH+OST-HTH+TUDOR+Thermonuclease                                      Tdrd5                  1040   Mus musculus                             metazoa>vertebrata                   tudor domain containing 5 [Mus musculus].  
73961278          OST-HTH+OST-HTH+OST-HTH+TUDOR+Thermonuclease                                      LOC480050              1035   Canis lupus familiaris                   metazoa>vertebrata                   PREDICTED: similar to tudor domain containing 5 [Canis familiaris].  
119611460         OST-HTH+OST-HTH+OST-HTH+TUDOR+Thermonuclease                                      TDRD5                  1035   Homo sapiens                             metazoa>vertebrata                   tudor domain containing 5, isoform CRA_c [Homo sapiens].  
120660256         OST-HTH+OST-HTH+OST-HTH+TUDOR+Thermonuclease                                      TDRD5                  1035   Homo sapiens                             metazoa>vertebrata                   TDRD5 protein [Homo sapiens].  
197927154         OST-HTH+OST-HTH+OST-HTH+TUDOR+Thermonuclease                                      Tdrd5                  995    Rattus norvegicus                        metazoa>vertebrata                   tudor domain containing 5 isoform b [Rattus norvegicus].  
149755084         OST-HTH+OST-HTH+OST-HTH+TUDOR+Thermonuclease                                      TDRD5                  988    Equus caballus                           metazoa>vertebrata                   PREDICTED: tudor domain containing 5 [Equus caballus].  
126306288         OST-HTH+OST-HTH+OST-HTH+TUDOR+Thermonuclease                                      LOC100017117           984    Monodelphis domestica                    metazoa>vertebrata                   PREDICTED: hypothetical protein [Monodelphis domestica].  
281351295         OST-HTH+OST-HTH+OST-HTH+TUDOR+Thermonuclease                                      PANDA_004307           982    Ailuropoda melanoleuca                   metazoa>vertebrata                   hypothetical protein PANDA_004307 [Ailuropoda melanoleuca].  
40255178          OST-HTH+OST-HTH+OST-HTH+TUDOR+Thermonuclease                                      TDRD5                  981    Homo sapiens                             metazoa>vertebrata                   tudor domain containing 5 [Homo sapiens].  
109019196         OST-HTH+OST-HTH+OST-HTH+TUDOR+Thermonuclease                                      TDRD5                  978    Macaca mulatta                           metazoa>vertebrata                   PREDICTED: tudor domain containing 5 isoform 2 [Macaca mulatta].  
118404676         OST-HTH+OST-HTH+TUDOR+Thermonuclease                                              tdrd5                  963    Xenopus (Silurana) tropicalis            metazoa>vertebrata                   tudor domain-containing protein 5 [Xenopus (Silurana) tropicalis].  
147903755         OST-HTH+OST-HTH+TUDOR+Thermonuclease                                              LOC100036842           963    Xenopus laevis                           metazoa>vertebrata                   hypothetical protein LOC100036842 [Xenopus laevis].  
157278855         OST-HTH+OST-HTH+TUDOR+Thermonuclease                                              Tdrd5                  945    Mus musculus                             metazoa>vertebrata                   Tdrd5 protein [Mus musculus].  
113678081         OST-HTH+OST-HTH+OST-HTH+TUDOR+Thermonuclease                                      tdrd5                  905    Danio rerio                              metazoa>vertebrata>actinopterygii    tudor domain containing 5 [Danio rerio].  
141795560         OST-HTH+OST-HTH+OST-HTH+TUDOR+Thermonuclease                                      tdrd5                  902    Danio rerio                              metazoa>vertebrata>actinopterygii    Tdrd5 protein [Danio rerio].  
59807731          OST-HTH+TUDOR+Thermonuclease                                                      TDRD5                  787    Homo sapiens                             metazoa>vertebrata                   TDRD5 protein [Homo sapiens].  
109019202         OST-HTH+TUDOR+Thermonuclease                                                      TDRD5                  787    Macaca mulatta                           metazoa>vertebrata                   PREDICTED: tudor domain containing 5 isoform 1 [Macaca mulatta].  
118094184         OST-HTH+TUDOR+Thermonuclease                                                      LOC424421              742    Gallus gallus                            metazoa>vertebrata                   PREDICTED: similar to Tudor domain-containing protein 5 [Gallus  
# 16;  
195382221         OST-HTH+DSRBD+TUDOR+TUDOR+TUDOR+Thermonuclease                                    Dvir\GJ20530           1865   Drosophila virilis                       metazoa>hexapoda                     GJ20530 [Drosophila virilis].  
195442037         OST-HTH+DSRBD+TUDOR+TUDOR+TUDOR+Thermonuclease                                    Dwil\GK17950           1758   Drosophila willistoni                    metazoa>hexapoda                     GK17950 [Drosophila willistoni].  
157125859         OST-HTH+DSRBD+TUDOR+TUDOR+TUDOR+Thermonuclease                                    AaeL_AAEL010311        1304   Aedes aegypti                            metazoa>hexapoda                     hypothetical protein AaeL_AAEL010311 [Aedes aegypti].  
195154869         OST-HTH+DSRBD+TUDOR+TUDOR+TUDOR+Thermonuclease                                    Dper\GL17657           1304   Drosophila persimilis                    metazoa>hexapoda                     GL17657 [Drosophila persimilis].  
198458782         OST-HTH+DSRBD+TUDOR+TUDOR+TUDOR+Thermonuclease                                    Dpse\GA24863           1302   Drosophila pseudoobscura pseudoobscura   metazoa>hexapoda                     GA24863 [Drosophila pseudoobscura pseudoobscura].  
194753448         OST-HTH+DSRBD+TUDOR+TUDOR+TUDOR+Thermonuclease                                    Dana\GF12261           1266   Drosophila ananassae                     metazoa>hexapoda                     GF12261 [Drosophila ananassae].  
62484261          OST-HTH+DSRBD+TUDOR+TUDOR+TUDOR+Thermonuclease                                    CG8920                 1222   Drosophila melanogaster                  metazoa>hexapoda                     CG8920, isoform B [Drosophila melanogaster].  
195123035         OST-HTH+DSRBD+TUDOR+TUDOR                                                         Dmoj\GI20795           1055   Drosophila mojavensis                    metazoa>hexapoda                     GI20795 [Drosophila mojavensis].  
170044611         OST-HTH+DSRBD+TUDOR+Thermonuclease+TUDOR                                          CpipJ_CPIJ007981       1042   Culex quinquefasciatus                   metazoa>hexapoda                     conserved hypothetical protein [Culex quinquefasciatus].  
194881557         OST-HTH+DSRBD+TUDOR+TUDOR                                                         Dere\GG22026           1040   Drosophila erecta                        metazoa>hexapoda                     GG22026 [Drosophila erecta].  
195028815         OST-HTH+DSRBD+TUDOR+TUDOR                                                         Dgri\GH21825           1006   Drosophila grimshawi                     metazoa>hexapoda                     GH21825 [Drosophila grimshawi].  
195486905         OST-HTH+DSRBD+TUDOR+TUDOR                                                         Dyak\GE12104           974    Drosophila yakuba                        metazoa>hexapoda                     GE12104 [Drosophila yakuba].  
51092095          OST-HTH+DSRBD+TUDOR+TUDOR                                                         -                      965    Drosophila melanogaster                  metazoa>hexapoda                     RE10852p [Drosophila melanogaster].  
281363827         OST-HTH+DSRBD+TUDOR+TUDOR                                                         CG8920                 965    Drosophila melanogaster                  metazoa>hexapoda                     CG8920, isoform D [Drosophila melanogaster].  
170062988         OST-HTH+DSRBD+TUDOR+Thermonuclease                                                CpipJ_CPIJ016676       794    Culex quinquefasciatus                   metazoa>hexapoda                     conserved hypothetical protein [Culex quinquefasciatus].  
195336080         OST-HTH+DSRBD+TUDOR                                                               Dsec\GM22008           714    Drosophila sechellia                     metazoa>hexapoda                     GM22008 [Drosophila sechellia].  
193678971         OST-HTH+DSRBD+TUDOR+TUDOR+Thermonuclease                                          LOC100161737           907    Acyrthosiphon pisum                      metazoa>hexapoda                     PREDICTED: similar to conserved hypothetical protein [Acyrthosiphon  
242025382         OST-HTH+DSRBD+TUDOR+TUDOR+TUDOR+Thermonuclease                                    Phum_PHUM613870        1141   Pediculus humanus corporis               metazoa>hexapoda                     conserved hypothetical protein [Pediculus humanus corporis].  
91084545          OST-HTH+DSRBD+TUDOR+TUDOR+TUDOR+Thermonuclease                                    LOC661845              1045   Tribolium castaneum                      metazoa>hexapoda                     PREDICTED: similar to CG8920 CG8920-PB [Tribolium castaneum].  
110764792         OST-HTH+DSRBD+TUDOR+Thermonuclease+TUDOR+TUDOR+Thermonuclease                     LOC726241              1043   Apis mellifera                           metazoa>hexapoda                     PREDICTED: similar to CG8920-PB, isoform B [Apis mellifera].  
156546224         OST-HTH+DSRBD+TUDOR+TUDOR+TUDOR+Thermonuclease                                    LOC100121226           1039   Nasonia vitripennis                      metazoa>hexapoda                     PREDICTED: similar to conserved hypothetical protein [Nasonia  
# 15;  
195426405         OST-HTH+NASP+TUDOR+Thermonuclease                                                 Dwil\GK20858           609    Drosophila willistoni                    metazoa>hexapoda                     GK20858 [Drosophila willistoni].  
198457785         OST-HTH+TUDOR+Thermonuclease                                                      Dpse\GA24775           594    Drosophila pseudoobscura pseudoobscura   metazoa>hexapoda                     GA24775 [Drosophila pseudoobscura pseudoobscura].  
195334308         OST-HTH+TUDOR+Thermonuclease                                                      Dsec\GM20217           563    Drosophila sechellia                     metazoa>hexapoda                     GM20217 [Drosophila sechellia].  
195485930         OST-HTH+TUDOR+Thermonuclease                                                      Dyak\GE12320           562    Drosophila yakuba                        metazoa>hexapoda                     GE12320 [Drosophila yakuba].  
194883116         OST-HTH+TUDOR+Thermonuclease                                                      Dere\GG22430           560    Drosophila erecta                        metazoa>hexapoda                     GG22430 [Drosophila erecta].  
24653610          OST-HTH+TUDOR+Thermonuclease                                                      CG8589                 559    Drosophila melanogaster                  metazoa>hexapoda                     CG8589 [Drosophila melanogaster].  
195583342         OST-HTH+NASP+TUDOR+Thermonuclease                                                 Dsim\GD25688           553    Drosophila simulans                      metazoa>hexapoda                     GD25688 [Drosophila simulans].  
194756914         OST-HTH+NASP+TUDOR+Thermonuclease                                                 Dana\GF11357           541    Drosophila ananassae                     metazoa>hexapoda                     GF11357 [Drosophila ananassae].  
195384351         OST-HTH+TUDOR+Thermonuclease                                                      Dvir\GJ19957           533    Drosophila virilis                       metazoa>hexapoda                     GJ19957 [Drosophila virilis].  
195170288         OST-HTH+TUDOR+Thermonuclease                                                      Dper\GL10200           529    Drosophila persimilis                    metazoa>hexapoda                     GL10200 [Drosophila persimilis].  
195028696         OST-HTH+NASP+TUDOR+Thermonuclease                                                 Dgri\GH20095           519    Drosophila grimshawi                     metazoa>hexapoda                     GH20095 [Drosophila grimshawi].  
195159274         OST-HTH+TUDOR+Thermonuclease                                                      Dper\GL13477           471    Drosophila persimilis                    metazoa>hexapoda                     GL13477 [Drosophila persimilis].  
198449617         OST-HTH+TUDOR+Thermonuclease                                                      Dpse\GA26863           471    Drosophila pseudoobscura pseudoobscura   metazoa>hexapoda                     GA26863 [Drosophila pseudoobscura pseudoobscura].  
198461026         OST-HTH+TUDOR+Thermonuclease                                                      Dpse\GA21185           456    Drosophila pseudoobscura pseudoobscura   metazoa>hexapoda                     GA21185 [Drosophila pseudoobscura pseudoobscura].  
195122158         OST-HTH+TUDOR+Thermonuclease                                                      Dmoj\GI18993           417    Drosophila mojavensis                    metazoa>hexapoda                     GI18993 [Drosophila mojavensis].  
# 3;  
17506361          OST-HTH+TUDOR                                                                     D1081.7                857    Caenorhabditis elegans                   metazoa>nematoda                     hypothetical protein D1081.7 [Caenorhabditis elegans].  
268567690         OST-HTH+TUDOR                                                                     CBG12533               857    Caenorhabditis briggsae                  metazoa>nematoda                     Hypothetical protein CBG12533 [Caenorhabditis briggsae].  
193202475         OST-HTH+TUDOR                                                                     D1081.7                855    Caenorhabditis elegans                   metazoa>nematoda                     hypothetical protein D1081.7 [Caenorhabditis elegans].  
# 2;  
198418545         OST-HTH+OST-HTH+OST-HTH+TUDOR+Thermonuclease                                      LOC100177761           849    Ciona intestinalis                       metazoa                              PREDICTED: similar to Tudor domain-containing protein 5 [Ciona  
260797443         OST-HTH+OST-HTH+OST-HTH+OST-HTH+TUDOR                                             BRAFLDRAFT_64001       584    Branchiostoma floridae                   metazoa                              hypothetical protein BRAFLDRAFT_64001 [Branchiostoma floridae].  
# 1; Tail  
241123253         OST-HTH+OST-HTH+TUDOR+Thermonuclease                                              IscW_ISCW002914        740    Ixodes scapularis                        metazoa                              hypothetical protein IscW_ISCW002914 [Ixodes scapularis].  
241842214         OST-HTH+OST-HTH+OST-HTH+TUDOR                                                     IscW_ISCW023428        538    Ixodes scapularis                        metazoa                              hypothetical protein IscW_ISCW023428 [Ixodes scapularis].  
198415060         OST-HTH+TUDOR+TUDOR+Thermonuclease+TUDOR+TUDOR+Thermonuclease                     LOC100183257           1151   Ciona intestinalis                       metazoa                              PREDICTED: similar to tudor domain containing protein 7 [Ciona  
241599806         OST-HTH+TUDOR+Thermonuclease+TUDOR+TUDOR                                          IscW_ISCW019360        951    Ixodes scapularis                        metazoa                              hypothetical protein IscW_ISCW019360 [Ixodes scapularis].  
260791599         OST-HTH+TUDOR+Thermonuclease+TUDOR+Thermonuclease                                 BRAFLDRAFT_125731      1095   Branchiostoma floridae                   metazoa                              hypothetical protein BRAFLDRAFT_125731 [Branchiostoma floridae].  
241570591         OST-HTH+TUDOR+Thermonuclease                                                      IscW_ISCW008071        1038   Ixodes scapularis                        metazoa                              A kinase anchor protein, putative [Ixodes scapularis].  
156387657         OST-HTH+OST-HTH+OST-HTH+TUDOR                                                     NEMVEDRAFT_v1g205481   590    Nematostella vectensis                   metazoa>cnidaria                     predicted protein [Nematostella vectensis].  
115768247         OST-HTH+OST-HTH+OST-HTH+TUDOR+Thermonuclease+Thermonuclease                       LOC578673              1552   Strongylocentrotus purpuratus            metazoa>echinodermata                PREDICTED: hypothetical protein [Strongylocentrotus purpuratus].  
115689733         OST-HTH+TUDOR+Thermonuclease                                                      LOC577641              683    Strongylocentrotus purpuratus            metazoa>echinodermata                PREDICTED: hypothetical protein [Strongylocentrotus purpuratus].  
170030338         OST-HTH+TUDOR+Thermonuclease                                                      CpipJ_CPIJ001562       497    Culex quinquefasciatus                   metazoa>hexapoda                     conserved hypothetical protein [Culex quinquefasciatus].  
58390124          OST-HTH+TUDOR+Thermonuclease                                                      AgaP_AGAP007965        470    Anopheles gambiae str. PEST              metazoa>hexapoda                     AGAP007965-PA [Anopheles gambiae str. PEST].  
193697693         OST-HTH+OST-HTH+TUDOR                                                             LOC100158689           810    Acyrthosiphon pisum                      metazoa>hexapoda                     PREDICTED: hypothetical protein, partial [Acyrthosiphon pisum].  
270001903         OST-HTH+TUDOR+Thermonuclease                                                      TcasGA2_TC000805       891    Tribolium castaneum                      metazoa>hexapoda                     hypothetical protein TcasGA2_TC000805 [Tribolium castaneum].  
156544082         OST-HTH+OST-HTH+TUDOR+Thermonuclease                                              LOC100121779           1647   Nasonia vitripennis                      metazoa>hexapoda                     PREDICTED: similar to conserved hypothetical protein [Nasonia  
118789562         OST-HTH+TUDOR                                                                     AgaP_AGAP007964        477    Anopheles gambiae str. PEST              metazoa>hexapoda                     AGAP007964-PA [Anopheles gambiae str. PEST].  
118104542         OST-HTH+TUDOR+TUDOR+TUDOR+Thermonuclease                                          LOC430492              898    Gallus gallus                            metazoa>vertebrata                   PREDICTED: hypothetical protein [Gallus gallus].  
149533223         OST-HTH+TUDOR+Thermonuclease                                                      LOC100088228           439    Ornithorhynchus anatinus                 metazoa>vertebrata                   PREDICTED: similar to tudor domain containing 7, partial  
47217386          OST-HTH+TUDOR                                                                     GSTEN:00019271:G:001   430    Tetraodon nigroviridis                   metazoa>vertebrata>actinopterygii    unnamed protein product [Tetraodon nigroviridis].  
47085847          OST-HTH+TUDOR+Thermonuclease                                                      tdrd7                  411    Danio rerio                              metazoa>vertebrata>actinopterygii    tudor domain containing 7 isoform 2 [Danio rerio].  
  
# 18;  
194903569         OST-HTH                                                                           Dere\GG13545           625    Drosophila erecta                        metazoa>hexapoda                     GG13545 [Drosophila erecta].  
195499262         OST-HTH                                                                           Dyak\GE25914           625    Drosophila yakuba                        metazoa>hexapoda                     GE25914 [Drosophila yakuba].  
195152922         OST-HTH                                                                           Dper\GL21554           619    Drosophila persimilis                    metazoa>hexapoda                     GL21554 [Drosophila persimilis].  
198454187         OST-HTH                                                                           Dpse\GA10627           619    Drosophila pseudoobscura pseudoobscura   metazoa>hexapoda                     GA10627 [Drosophila pseudoobscura pseudoobscura].  
195572425         OST-HTH                                                                           Dsim\GD18580           611    Drosophila simulans                      metazoa>hexapoda                     GD18580 [Drosophila simulans].  
195330556         OST-HTH                                                                           Dsec\GM23770           610    Drosophila sechellia                     metazoa>hexapoda                     GM23770 [Drosophila sechellia].  
24645205          OST-HTH                                                                           osk                    606    Drosophila melanogaster                  metazoa>hexapoda                     oskar, isoform A [Drosophila melanogaster].  
194741640         OST-HTH                                                                           Dana\GF17692           602    Drosophila ananassae                     metazoa>hexapoda                     GF17692 [Drosophila ananassae].  
195054868         OST-HTH                                                                           Dgri\GH23955           600    Drosophila grimshawi                     metazoa>hexapoda                     GH23955 [Drosophila grimshawi].  
195111098         OST-HTH                                                                           Dmoj\GI10055           600    Drosophila mojavensis                    metazoa>hexapoda                     GI10055 [Drosophila mojavensis].  
195389208         OST-HTH                                                                           Dvir\osk               595    Drosophila virilis                       metazoa>hexapoda                     oskar [Drosophila virilis].  
2498716           OST-HTH                                                                           osk                    594    Drosophila virilis                       metazoa>hexapoda                     RecName: Full=Maternal effect protein oskar.  
111663086         OST-HTH                                                                           oskar                  552    Drosophila immigrans                     metazoa>hexapoda                     oskar [Drosophila immigrans].  
111663088         OST-HTH                                                                           oskar                  552    Drosophila immigrans                     metazoa>hexapoda                     oskar [Drosophila immigrans].  
157134733         OST-HTH                                                                           AaeL_AAEL000442        476    Aedes aegypti                            metazoa>hexapoda                     hypothetical protein AaeL_AAEL000442 [Aedes aegypti].  
118783859         OST-HTH                                                                           AgaP_AGAP003545        407    Anopheles gambiae str. PEST              metazoa>hexapoda                     AGAP003545-PA [Anopheles gambiae str. PEST].  
170041806         OST-HTH                                                                           CpipJ_CPIJ007471       403    Culex quinquefasciatus                   metazoa>hexapoda                     oskar [Culex quinquefasciatus].  
195445337         OST-HTH                                                                           Dwil\GK11116           358    Drosophila willistoni                    metazoa>hexapoda                     GK11116 [Drosophila willistoni].  
# 10;  
218187168         ANK+RRM+OST-HTH+UBI                                                               OsI_38941              1371   Oryza sativa Indica Group                viridiplantae                        hypothetical protein OsI_38941 [Oryza sativa Indica Group].  
14346032          ANK+RRM+OST-HTH+UBI                                                               -                      1161   Oryza sativa Indica Group                viridiplantae                        RSSG8 [Oryza sativa Indica Group].  
222617379         ANK+ANK+RRM+OST-HTH+UBI                                                           OsJ_36687              1441   Oryza sativa Japonica Group              viridiplantae                        hypothetical protein OsJ_36687 [Oryza sativa Japonica Group].  
108862897         ANK+ANK+RRM+OST-HTH+UBI                                                           LOC_Os12g39700         1618   Oryza sativa Japonica Group              viridiplantae                        retrotransposon protein, putative, unclassified [Oryza sativa  
255670419         ANK+RRM+OST-HTH+RING+RING                                                         Os12g0572800           1233   Oryza sativa Japonica Group              viridiplantae                        Os12g0572800 [Oryza sativa Japonica Group].  
125537113         ANK+ANK+RRM+OST-HTH+RING+RING                                                     OsI_38823              1134   Oryza sativa Indica Group                viridiplantae                        hypothetical protein OsI_38823 [Oryza sativa Indica Group].  
115489212         ANK+ANK+RRM+OST-HTH+RING+RING                                                     Os12g0572800           1132   Oryza sativa Japonica Group              viridiplantae                        Os12g0572800 [Oryza sativa (japonica cultivar-group)].  
242085946         ANK+RRM+OST-HTH+RING+RING                                                         SORBIDRAFT_08g018890   1083   Sorghum bicolor                          viridiplantae                        hypothetical protein SORBIDRAFT_08g018890 [Sorghum bicolor].  
255566771         ANK+OST-HTH+RING                                                                  RCOM_0703810           707    Ricinus communis                         viridiplantae                        ankyrin repeat-containing protein, putative [Ricinus communis].  
224077602         ANK+CCCH+RRM+OST-HTH                                                              POPTRDRAFT_759364      1097   Populus trichocarpa                      viridiplantae                        predicted protein [Populus trichocarpa].  
147834593         ANK+CCCH+RRM+OST-HTH+RING                                                         VITISV_028449          1609   Vitis vinifera                           viridiplantae                        hypothetical protein [Vitis vinifera].  
225452793         ANK+CCCH+RRM+OST-HTH+RING                                                         LOC100264593           1061   Vitis vinifera                           viridiplantae                        PREDICTED: hypothetical protein [Vitis vinifera].  
270232882         ANK+CCCH+RRM+OST-HTH+RING                                                         GSVIVT01011394001      849    Vitis vinifera                           viridiplantae                        unnamed protein product [Vitis vinifera].  
168025460         CCCH+RRM+OST-HTH+RING                                                             PHYPADRAFT_78748       934    Physcomitrella patens subsp. patens      viridiplantae                        predicted protein [Physcomitrella patens subsp. patens].  
Smoe1000009123    RRM+OST-HTH+RING                                                                  Smoe1000009123         447    Selaginella moellendorffii               viridiplantae                        fgenesh2_pg.C_scaffold_114000059  
Smoe1000009128    RRM+OST-HTH+RING                                                                  Smoe1000009128         311    Selaginella moellendorffii               viridiplantae                        fgenesh2_pg.C_scaffold_114000054  
# 9;  
255587629         CCCH+OST-HTH+RRM                                                                  RCOM_0408340           578    Ricinus communis                         viridiplantae                        RNA binding protein, putative [Ricinus communis].  
255576322         CCCH+OST-HTH+RRM                                                                  RCOM_0047440           551    Ricinus communis                         viridiplantae                        RNA binding protein, putative [Ricinus communis].  
15224480          CCCH+OST-HTH+RRM                                                                  AT2G05160              536    Arabidopsis thaliana                     viridiplantae                        zinc finger (CCCH-type) family protein / RNA recognition motif  
75328302          CCCH+OST-HTH+RRM                                                                  Os07g0583300           513    Oryza sativa Japonica Group              viridiplantae                        RecName: Full=Putative zinc finger CCCH domain-containing protein  
125558949         CCCH+OST-HTH+RRM                                                                  OsI_26636              432    Oryza sativa Indica Group                viridiplantae                        hypothetical protein OsI_26636 [Oryza sativa Indica Group].  
125600867         CCCH+OST-HTH+RRM                                                                  OsJ_24897              432    Oryza sativa Japonica Group              viridiplantae                        hypothetical protein OsJ_24897 [Oryza sativa Japonica Group].  
168011119         CCCH+OST-HTH+RRM                                                                  PHYPADRAFT_119974      421    Physcomitrella patens subsp. patens      viridiplantae                        predicted protein [Physcomitrella patens subsp. patens].  
168037568         CCCH+OST-HTH+RRM                                                                  PHYPADRAFT_137259      386    Physcomitrella patens subsp. patens      viridiplantae                        predicted protein [Physcomitrella patens subsp. patens].  
15231722          CCCH+OST-HTH+RRM                                                                  AT3G52980              381    Arabidopsis thaliana                     viridiplantae                        RNA recognition motif (RRM)-containing protein [Arabidopsis  
Smoe1000018699    CCCH+OST-HTH+RRM                                                                  Smoe1000018699         524    Selaginella moellendorffii               viridiplantae                        estExt_fgenesh2_pg.C_00420  
Smoe1000013793    CCCH+OST-HTH+RRM                                                                  Smoe1000013793         395    Selaginella moellendorffii               viridiplantae                        e_gw1.43.473.1  
Smoe1000015858    CCCH+OST-HTH+RRM                                                                  Smoe1000015858         362    Selaginella moellendorffii               viridiplantae                        fgenesh1_pm.C_scaffold_139000002  
Smoe1000017142    CCCH+OST-HTH+RRM                                                                  Smoe1000017142         356    Selaginella moellendorffii               viridiplantae                        e_gw1.24.612.1  
Smoe1000011346    CCCH+OST-HTH+RRM                                                                  Smoe1000011346         205    Selaginella moellendorffii               viridiplantae                        gw1.10.916.1  
Smoe1000005181    CCCH+OST-HTH+RRM                                                                  Smoe1000005181         198    Selaginella moellendorffii               viridiplantae                        gw1.68.205.1  
# 4;  
239875739         WW+OHA+OST-HTH                                                                    Pmar_PMAR023053        488    Perkinsus marinus ATCC 50983             alveolata                            hypothetical protein Pmar_PMAR023053 [Perkinsus marinus ATCC  
156087541         WW+OHA+OST-HTH                                                                    BBOV_III000420         571    Babesia bovis T2Bo                       alveolata>apicomplexa                hypothetical protein [Babesia bovis T2Bo].  
156089481         WW+OHA+OST-HTH                                                                    BBOV_III010230         413    Babesia bovis T2Bo                       alveolata>apicomplexa                hypothetical protein [Babesia bovis T2Bo].  
209881271         WW+OHA+OST-HTH                                                                    CMU_006480             508    Cryptosporidium muris RN66               alveolata>apicomplexa                hypothetical protein [Cryptosporidium muris RN66].  
209880995         WW+OHA+OST-HTH                                                                    CMU_005100             555    Cryptosporidium muris RN66               alveolata>apicomplexa                hypothetical protein [Cryptosporidium muris RN66].  
126652095         WW+OHA+OST-HTH                                                                    cgd7_410               405    Cryptosporidium parvum Iowa II           alveolata>apicomplexa                hypothetical protein [Cryptosporidium parvum Iowa II].  
68060968          WW+OHA+OST-HTH                                                                    PB300490.00.0          280    Plasmodium berghei str. ANKA             alveolata>apicomplexa                hypothetical protein [Plasmodium berghei strain ANKA].  
68065788          WW+OHA+WW+OHA+OST-HTH+FDF                                                         PB001068.03.0          750    Plasmodium berghei str. ANKA             alveolata>apicomplexa                hypothetical protein [Plasmodium berghei strain ANKA].  
68073861          WW+OHA+OST-HTH                                                                    PB000432.02.0          1180   Plasmodium berghei str. ANKA             alveolata>apicomplexa                hypothetical protein [Plasmodium berghei strain ANKA].  
68075603          WW+OHA+OST-HTH                                                                    PB000038.01.0          365    Plasmodium berghei str. ANKA             alveolata>apicomplexa                hypothetical protein [Plasmodium berghei strain ANKA].  
70943942          WW+OHA+OST-HTH                                                                    PC000282.00.0          311    Plasmodium chabaudi chabaudi             alveolata>apicomplexa                hypothetical protein [Plasmodium chabaudi chabaudi].  
70948668          WW+OHA+OST-HTH+FDF                                                                PC001208.02.0          970    Plasmodium chabaudi chabaudi             alveolata>apicomplexa                hypothetical protein [Plasmodium chabaudi chabaudi].  
124809304         WW+OHA+OST-HTH                                                                    PF14_0367              700    Plasmodium falciparum 3D7                alveolata>apicomplexa                conserved Plasmodium protein, unknown function [Plasmodium  
86170701          WW+OHA+WW+OHA+OST-HTH                                                             PFF0380w               2752   Plasmodium falciparum 3D7                alveolata>apicomplexa                hypothetical protein [Plasmodium falciparum 3D7].  
124808633         WW+OHA+OST-HTH                                                                    PF14_0195              1449   Plasmodium falciparum 3D7                alveolata>apicomplexa                conserved Plasmodium protein, unknown function [Plasmodium  
124809004         WW+OHA+WW+OHA+OST-HTH                                                             PF14_0291              1225   Plasmodium falciparum 3D7                alveolata>apicomplexa                conserved Plasmodium protein, unknown function [Plasmodium  
221060132         WW+OHA+WW+OHA+OST-HTH                                                             PKH_131910             948    Plasmodium knowlesi strain H             alveolata>apicomplexa                hypothetical protein, conserved in Plasmodium species [Plasmodium  
221059679         WW+OHA+OST-HTH                                                                    PKH_126750             897    Plasmodium knowlesi strain H             alveolata>apicomplexa                hypothetical protein, conserved in Plasmodium species [Plasmodium  
221060328         WW+OHA+OST-HTH                                                                    PKH_132890             1525   Plasmodium knowlesi strain H             alveolata>apicomplexa                hypothetical protein, conserved in Plasmodium species [Plasmodium  
221058180         WW+OHA+WW+OHA+OST-HTH                                                             PKH_114160             2670   Plasmodium knowlesi strain H             alveolata>apicomplexa                hypothetical protein, conserved in Plasmodium species [Plasmodium  
156100551         WW+OHA+OST-HTH                                                                    PVX_118550             937    Plasmodium vivax SaI-1                   alveolata>apicomplexa                hypothetical protein [Plasmodium vivax SaI-1].  
156102132         WW+OHA+OST-HTH                                                                    PVX_085475             1597   Plasmodium vivax SaI-1                   alveolata>apicomplexa                hypothetical protein [Plasmodium vivax SaI-1].  
82914991          WW+OHA                                                                            PY01201                1493   Plasmodium yoelii yoelii str. 17XNL      alveolata>apicomplexa                hypothetical protein [Plasmodium yoelii yoelii str. 17XNL].  
82539444          WW+OHA+OST-HTH                                                                    PY00415                577    Plasmodium yoelii yoelii str. 17XNL      alveolata>apicomplexa                hypothetical protein [Plasmodium yoelii yoelii str. 17XNL].  
82593955          WW+OHA+WW+OHA+OST-HTH+FDF                                                         PY04854                1980   Plasmodium yoelii yoelii str. 17XNL      alveolata>apicomplexa                hypothetical protein [Plasmodium yoelii yoelii str. 17XNL].  
85001524          WW+OHA+OST-HTH                                                                    TA18280                785    Theileria annulata strain Ankara         alveolata>apicomplexa                hypothetical protein [Theileria annulata].  
84995728          WW+OHA+OST-HTH                                                                    TA11490                423    Theileria annulata strain Ankara         alveolata>apicomplexa                hypothetical protein [Theileria annulata].  
71026501          WW+OHA+OST-HTH                                                                    TP03_0796              320    Theileria parva strain Muguga            alveolata>apicomplexa                hypothetical protein [Theileria parva strain Muguga].  
71030104          WW+OHA+OST-HTH                                                                    TP02_0125              436    Theileria parva strain Muguga            alveolata>apicomplexa                hypothetical protein [Theileria parva strain Muguga].  
221481924         WW+OHA                                                                            TGGT1_062120           700    Toxoplasma gondii GT1                    alveolata>apicomplexa                conserved hypothetical protein [Toxoplasma gondii GT1].  
221485777         WW+OHA+OST-HTH                                                                    TGGT1_045580           340    Toxoplasma gondii GT1                    alveolata>apicomplexa                conserved hypothetical protein [Toxoplasma gondii GT1].  
237835255         WW+OHA+OST-HTH                                                                    TGME49_044360          340    Toxoplasma gondii ME49                   alveolata>apicomplexa                hypothetical protein, conserved [Toxoplasma gondii ME49].  
237837091         WW+OHA                                                                            TGME49_006620          700    Toxoplasma gondii ME49                   alveolata>apicomplexa                hypothetical protein TGME49_006620 [Toxoplasma gondii ME49].  
221501378         WW+OHA                                                                            TGVEG_000200           700    Toxoplasma gondii VEG                    alveolata>apicomplexa                conserved hypothetical protein [Toxoplasma gondii VEG].  
145497923         WW+OHA+OST-HTH+OST-HTH                                                            GSPATT00006860001      651    Paramecium tetraurelia strain d4-2       alveolata>ciliophora                 hypothetical protein [Paramecium tetraurelia strain d4-2].  
145527744         WW+OHA+OST-HTH                                                                    GSPATT00002404001      675    Paramecium tetraurelia strain d4-2       alveolata>ciliophora                 hypothetical protein [Paramecium tetraurelia strain d4-2].  
145496001         WW+OHA+OST-HTH                                                                    GSPATT00035725001      706    Paramecium tetraurelia strain d4-2       alveolata>ciliophora                 hypothetical protein [Paramecium tetraurelia strain d4-2].  
145510865         BBOX+WW+OHA+OST-HTH                                                               GSPATT00010196001      909    Paramecium tetraurelia strain d4-2       alveolata>ciliophora                 hypothetical protein [Paramecium tetraurelia strain d4-2].  
145490150         BBOX+WW+OHA+OST-HTH                                                               GSPATT00033531001      912    Paramecium tetraurelia strain d4-2       alveolata>ciliophora                 hypothetical protein [Paramecium tetraurelia strain d4-2].  
146181541         BBOX+WW+OHA+OST-HTH                                                               TTHERM_00353270        1928   Tetrahymena thermophila                  alveolata>ciliophora                 B-box zinc finger family protein [Tetrahymena thermophila].  
# 2;  
Psoj1000010146    TUDOR+OST-HTH+WW                                                                  Psoj1000010146         5077   Phytophthora sojae                       stramenopiles                        137309  
Pram1000000908    SFI1+TUDOR+MORN+MORN+MORN+MORN+OST-HTH+WW                                         Pram1000000908         3507   Phytophthora ramorum                     stramenopiles                        83992  
Pram1000012897    OST-HTH+WW                                                                        Pram1000012897         351    Phytophthora ramorum                     stramenopiles                        87873  
# 2;  
66358626          KH+OST-HTH                                                                        cgd2_2940              1132   Cryptosporidium parvum Iowa II           alveolata>apicomplexa                hypothetical protein [Cryptosporidium parvum Iowa II].  
67611650          KH+OST-HTH                                                                        Chro.20308             1132   Cryptosporidium hominis TU502            alveolata>apicomplexa                hypothetical protein [Cryptosporidium hominis TU502].  
209882957         KH+OST-HTH                                                                        CMU_030530             789    Cryptosporidium muris RN66               alveolata>apicomplexa                KH domain-containing protein [Cryptosporidium muris RN66].  
# 2;  
186510546         ANK+OST-HTH+RING                                                                  AT3G28880              772    Arabidopsis thaliana                     viridiplantae                        protein binding [Arabidopsis thaliana].  
9294229           ANK+OST-HTH+RING                                                                  -                      738    Arabidopsis thaliana                     viridiplantae                        unnamed protein product [Arabidopsis thaliana].  
# 2;  
148707438         OST-HTH+OST-HTH                                                                   -                      321    Mus musculus                             metazoa>vertebrata                   mCG126116, isoform CRA_b [Mus musculus].  
149058333         OST-HTH                                                                           rCG_46180              302    Rattus norvegicus                        metazoa>vertebrata                   rCG46180 [Rattus norvegicus].  
# 2;  
195351636         OST-HTH                                                                           Dsec\GM13340           148    Drosophila sechellia                     metazoa>hexapoda                     GM13340 [Drosophila sechellia].  
195351704         OST-HTH                                                                           Dsec\GM13504           148    Drosophila sechellia                     metazoa>hexapoda                     GM13504 [Drosophila sechellia].  
# 1;  
115608823         OST-HTH                                                                           LOC586486              233    Strongylocentrotus purpuratus            metazoa>echinodermata                PREDICTED: hypothetical protein, partial [Strongylocentrotus  
195585071         OST-HTH                                                                           Dsim\GD11508           133    Drosophila simulans                      metazoa>hexapoda                     GD11508 [Drosophila simulans].  
168054254         OST-HTH                                                                           PHYPADRAFT_94425       871    Physcomitrella patens subsp. patens      viridiplantae                        predicted protein [Physcomitrella patens subsp. patens].  
242067539         OST-HTH                                                                           SORBIDRAFT_05g003965   237    Sorghum bicolor                          viridiplantae                        hypothetical protein SORBIDRAFT_05g003965 [Sorghum bicolor].  
67624177          OST-HTH                                                                           Chro.70054             154    Cryptosporidium hominis TU502            alveolata>apicomplexa                hypothetical protein [Cryptosporidium hominis TU502].  
57971128          OST-HTH                                                                           GPRNND1                274    Anopheles gambiae str. PEST              metazoa>hexapoda                     putative GPCR class d orphan receptor 1 (AGAP011090-PA) [Anopheles  
221115123         OST-HTH                                                                           LOC100204243           282    Hydra magnipapillata                     metazoa>cnidaria                     PREDICTED: similar to predicted protein [Hydra magnipapillata].  
260789014         OST-HTH                                                                           BRAFLDRAFT_232620      64     Branchiostoma floridae                   metazoa                              hypothetical protein BRAFLDRAFT_232620 [Branchiostoma floridae].  
242011130         OST-HTH                                                                           Phum_PHUM247930        126    Pediculus humanus corporis               metazoa>hexapoda                     hypothetical protein Phum_PHUM247930 [Pediculus humanus corporis].  
47220003          OST-HTH                                                                           GSTEN:00033526:G:001   83     Tetraodon nigroviridis                   metazoa>vertebrata>actinopterygii    unnamed protein product [Tetraodon nigroviridis].  
115761941         OST-HTH                                                                           LOC766210              78     Strongylocentrotus purpuratus            metazoa>echinodermata                PREDICTED: similar to RE40762p, partial [Strongylocentrotus  
109487677         OST-HTH                                                                           LOC688442              322    Rattus norvegicus                        metazoa>vertebrata                   PREDICTED: similar to limkain b1 [Rattus norvegicus].  
221129949         OST-HTH                                                                           LOC100207520           338    Hydra magnipapillata                     metazoa>cnidaria                     PREDICTED: hypothetical protein [Hydra magnipapillata].  
170056257         OST-HTH                                                                           CpipJ_CPIJ013576       276    Culex quinquefasciatus                   metazoa>hexapoda                     conserved hypothetical protein [Culex quinquefasciatus].  
195567280         OST-HTH                                                                           Dsim\GD15702           67     Drosophila simulans                      metazoa>hexapoda                     GD15702 [Drosophila simulans].  
193598795         OST-HTH                                                                           LOC100164016           380    Acyrthosiphon pisum                      metazoa>hexapoda                     PREDICTED: similar to CG8589 CG8589-PA, partial [Acyrthosiphon  
241710168         OST-HTH                                                                           IscW_ISCW010856        450    Ixodes scapularis                        metazoa                              hypothetical protein IscW_ISCW010856 [Ixodes scapularis].  
224092085         OST-HTH                                                                           POPTRDRAFT_791332      530    Populus trichocarpa                      viridiplantae                        predicted protein [Populus trichocarpa].  
167525489         OST-HTH+CCCH+LittleFinger+ANK+ANK                                                 MONBRDRAFT_26716       973    Monosiga brevicollis MX1                 choanoflagellida                     hypothetical protein [Monosiga brevicollis MX1].  
156352303         OST-HTH+OST-HTH                                                                   NEMVEDRAFT_v1g175950   279    Nematostella vectensis                   metazoa>cnidaria                     hypothetical protein NEMVEDRAFT_v1g175950 [Nematostella vectensis].  
119579247         OST-HTH+OST-HTH                                                                   TDRD7                  532    Homo sapiens                             metazoa>vertebrata                   tudor domain containing 7, isoform CRA_a [Homo sapiens].  
148707437         OST-HTH+OST-HTH                                                                   -                      274    Mus musculus                             metazoa>vertebrata                   mCG126116, isoform CRA_a [Mus musculus].  
189525705         OST-HTH+OST-HTH+OST-HTH                                                           LOC100006616           343    Danio rerio                              metazoa>vertebrata>actinopterygii    PREDICTED: hypothetical protein, partial [Danio rerio].  
168048576         OST-HTH+OST-HTH+OST-HTH                                                           PHYPADRAFT_91454       1414   Physcomitrella patens subsp. patens      viridiplantae                        predicted protein [Physcomitrella patens subsp. patens].  
189525662         OST-HTH+OST-HTH+OST-HTH                                                           LOC100148211           1025   Danio rerio                              metazoa>vertebrata>actinopterygii    PREDICTED: hypothetical protein [Danio rerio].  
198415080         OST-HTH+OST-HTH+OST-HTH+OST-HTH                                                   LOC100177790           718    Ciona intestinalis                       metazoa                              PREDICTED: similar to limkain b1 [Ciona intestinalis].  
239897854         RRM+HD_GLND+OST-HTH                                                               Pmar_PMAR021063        354    Perkinsus marinus ATCC 50983             alveolata                            hypothetical protein Pmar_PMAR021063 [Perkinsus marinus ATCC  
Aano1000005602    OST-HTH+OST-HTH+RRM+OST-HTH+RRM+RRM                                               Aano1000005602         3343   Aureococcus anophagefferens              stramenopiles                        71372  
Aano1000008826    RRM+RRM+OST-HTH+UBOX+S1COLD+CCCH+OST-HTH+UBOX+OST-HTH                             Aano1000008826         2507   Aureococcus anophagefferens              stramenopiles                        72103  
Caps1000009677    OST-HTH                                                                           Caps1000009677         628    Capitella spI                            metazoa>annelida                     fgenesh1_pg.C_scaffold_2115000001  
Caps1000025556    RRM+RRM+OST-HTH+OST-HTH                                                           Caps1000025556         1029   Capitella spI                            metazoa>annelida                     fgenesh1_pg.C_scaffold_517000007  
Caps1000025558    OST-HTH+OST-HTH+OST-HTH                                                           Caps1000025558         505    Capitella spI                            metazoa>annelida                     fgenesh1_pg.C_scaffold_517000009  
Caps1000027389    OST-HTH                                                                           Caps1000027389         71     Capitella spI                            metazoa>annelida                     e_gw1.4807.1.1  
Hrob1000008257    RRM+RRM+OST-HTH+OST-HTH+OST-HTH                                                   Hrob1000008257         1920   Helobdella robusta                       metazoa>annelida                     190347  
Lgig1000014581    OST-HTH                                                                           Lgig1000014581         263    Lottia gigantea                          metazoa>mollusca                     fgenesh2_pg.C_sca_112000018  
Smoe1000005539    OST-HTH                                                                           Smoe1000005539         316    Selaginella moellendorffii               viridiplantae                        fgenesh2_pg.C_scaffold_47000136  
Smoe1000011227    OST-HTH                                                                           Smoe1000011227         746    Selaginella moellendorffii               viridiplantae                        estExt_fgenesh2_pg.C_100228  
Caps1000018855    OST-HTH+RRM+RRM+TUDOR                                                             Caps1000018855         831    Capitella spI                            metazoa>annelida                     fgenesh1_pg.C_scaffold_340000010  
Hrob1000004659    OST-HTH+RRM+TUDOR                                                                 Hrob1000004659         608    Helobdella robusta                       metazoa>annelida                     163664  
Hrob1000011607    OST-HTH+RRM+TUDOR                                                                 Hrob1000011607         712    Helobdella robusta                       metazoa>annelida                     171620  
  
  
# 330; Bacterial  
159121942         LK-Nuclease+OST-HTH                                                               AFUB_102220            178    Aspergillus fumigatus A1163                                     fungi>ascomycota                       hypothetical protein AFUB_102220 [Aspergillus fumigatus A1163].  
67904058          LK-Nuclease+OST-HTH                                                               AN9016.2               404    Aspergillus nidulans FGSC A4                                    fungi>ascomycota                       hypothetical protein AN9016.2 [Aspergillus nidulans FGSC A4].  
145241746         LK-Nuclease+OST-HTH                                                               An09g02040             253    Aspergillus niger CBS 513.88                                    fungi>ascomycota                       hypothetical protein An09g02040 [Aspergillus niger].  
169772059         LK-Nuclease+OST-HTH                                                               AO090003001445         209    Aspergillus oryzae RIB40                                        fungi>ascomycota                       hypothetical protein [Aspergillus oryzae RIB40].  
256725266         LK-Nuclease+OST-HTH                                                               NECHADRAFT_62469       262    Nectria haematococca mpVI 77-13-4                               fungi>ascomycota                       hypothetical protein NECHADRAFT_62469 [Nectria haematococca mpVI  
255931419         LK-Nuclease+OST-HTH                                                               Pc12g03870             259    Penicillium chrysogenum Wisconsin 54-1255                       fungi>ascomycota                       Pc12g03870 [Penicillium chrysogenum Wisconsin 54-1255].  
255935257         LK-Nuclease+OST-HTH                                                               Pc13g02120             259    Penicillium chrysogenum Wisconsin 54-1255                       fungi>ascomycota                       Pc13g02120 [Penicillium chrysogenum Wisconsin 54-1255].  
169611408         LK-Nuclease+OST-HTH                                                               SNOG_08816             277    Phaeosphaeria nodorum SN15                                      fungi>ascomycota                       hypothetical protein SNOG_08816 [Phaeosphaeria nodorum SN15].  
189210104         LK-Nuclease+OST-HTH                                                               PTRG_11053             260    Pyrenophora tritici-repentis Pt-1C-BFP                          fungi>ascomycota                       conserved hypothetical protein [Pyrenophora tritici-repentis  
261361271         LK-Nuclease+OST-HTH                                                               VDBG_09809             264    Verticillium albo-atrum VaMs.102                                fungi>ascomycota                       conserved hypothetical protein [Verticillium albo-atrum VaMs.102].  
91772560          LK-Nuclease+OST-HTH                                                               Mbur_0520              262    Methanococcoides burtonii DSM 6242                              euryarchaeota                          hypothetical protein Mbur_0520 [Methanococcoides burtonii DSM  
147920970         LK-Nuclease+OST-HTH                                                               RCIX466                254    uncultured methanogenic archaeon RC-I                           euryarchaeota                          hypothetical protein RCIX466 [uncultured methanogenic archaeon  
255292460         LK-Nuclease+OST-HTH                                                               -                      238    uncultured bacterium                                                                                   hypothetical protein [uncultured bacterium].  
257784783         LK-Nuclease+OST-HTH                                                               Apar_0980              260    Atopobium parvulum DSM 20469                                    actinobacteria                         protein of unknown function LK-Nuclease [Atopobium parvulum DSM 20469].  
221195605         LK-Nuclease+OST-HTH                                                               ATORI0001_0115         256    Atopobium rimae ATCC 49626                                      actinobacteria                         conserved hypothetical protein [Atopobium rimae ATCC 49626].  
227516716         LK-Nuclease+OST-HTH                                                               HMPREF0091_0971        260    Atopobium vaginae DSM 15829                                     actinobacteria                         conserved hypothetical protein [Atopobium vaginae DSM 15829].  
229819539         LK-Nuclease+OST-HTH                                                               Bcav_1042              378    Beutenbergia cavernae DSM 12333                                 actinobacteria                         protein of unknown function LK-Nuclease [Beutenbergia cavernae DSM  
257068962         LK-Nuclease+OST-HTH                                                               Bfae_18070             335    Brachybacterium faecium DSM 4810                                actinobacteria                         Protein of unknown function LK-Nuclease [Brachybacterium faecium DSM  
256396571         LK-Nuclease+OST-HTH                                                               Caci_7469              260    Catenulispora acidiphila DSM 44928                              actinobacteria                         protein of unknown function LK-Nuclease [Catenulispora acidiphila DSM  
229815424         LK-Nuclease+OST-HTH                                                               COLINT_02472           301    Collinsella intestinalis DSM 13280                              actinobacteria                         hypothetical protein COLINT_02472 [Collinsella intestinalis DSM  
162447870         LK-Nuclease+OST-HTH                                                               ACL_1016               242    Acholeplasma laidlawii PG-8A                                    tenericutes                            hypothetical protein ACL_1016 [Acholeplasma laidlawii PG-8A].  
257791681         LK-Nuclease+OST-HTH                                                               Elen_1934              552    Eggerthella lenta DSM 2243                                      actinobacteria                         protein of unknown function LK-Nuclease [Eggerthella lenta DSM 2243].  
158314589         LK-Nuclease+OST-HTH                                                               Franean1_2768          272    Frankia sp. EAN1pec                                             actinobacteria                         hypothetical protein Franean1_2768 [Frankia sp. EAN1pec].  
280961430         LK-Nuclease+OST-HTH                                                               FraEuI1cDRAFT_2052     314    Frankia sp. EuI1c                                               actinobacteria                         protein of unknown function LK-Nuclease [Frankia sp. EuI1c].  
84495933          LK-Nuclease+OST-HTH                                                               JNB_00400              309    Janibacter sp. HTCC2649                                         actinobacteria                         hypothetical protein JNB_00400 [Janibacter sp. HTCC2649].  
256824346         LK-Nuclease+OST-HTH                                                               Ksed_04690             412    Kytococcus sedentarius DSM 20547                                actinobacteria                         hypothetical protein Ksed_04690 [Kytococcus sedentarius DSM 20547].  
50955250          LK-Nuclease+OST-HTH                                                               Lxx16560               362    Leifsonia xyli subsp. xyli str. CTCB07                          actinobacteria                         hypothetical protein Lxx16560 [Leifsonia xyli subsp. xyli str.  
239918004         LK-Nuclease+OST-HTH                                                               Mlut_15160             371    Micrococcus luteus NCTC 2665                                    actinobacteria                         conserved hypothetical protein TIGR00288 [Micrococcus luteus NCTC  
270500325         LK-Nuclease+OST-HTH                                                               MicauDRAFT_2251        356    Micromonospora aurantiaca ATCC 27029                            actinobacteria                         protein of unknown function LK-Nuclease [Micromonospora aurantiaca ATCC  
229214908         LK-Nuclease+OST-HTH                                                               DpepDRAFT_21110        274    Dethiosulfovibrio peptidovorans DSM 11002                       synergistetes                          Protein of unknown function LK-Nuclease [Dethiosulfovibrio peptidovorans  
118463148         LK-Nuclease+OST-HTH                                                               MAV_4568               284    Mycobacterium avium 104                                         actinobacteria                         hypothetical protein MAV_4568 [Mycobacterium avium 104].  
254776999         LK-Nuclease+OST-HTH                                                               MaviaA2_010100020351   296    Mycobacterium avium subsp. avium ATCC 25291                     actinobacteria                         hypothetical protein MaviaA2_20351 [Mycobacterium avium subsp.  
41410167          LK-Nuclease+OST-HTH                                                               MAP4069c               292    Mycobacterium avium subsp. paratuberculosis K-10                actinobacteria                         hypothetical protein MAP4069c [Mycobacterium avium subsp.  
145225022         LK-Nuclease+OST-HTH                                                               Mflv_4443              329    Mycobacterium gilvum PYR-GCK                                    actinobacteria                         hypothetical protein Mflv_4443 [Mycobacterium gilvum PYR-GCK].  
240171215         LK-Nuclease+OST-HTH                                                               MkanA1_010100018021    289    Mycobacterium kansasii ATCC 12478                               actinobacteria                         hypothetical protein MkanA1_18021 [Mycobacterium kansasii ATCC  
183982600         LK-Nuclease+OST-HTH                                                               MMAR_2590              288    Mycobacterium marinum M                                         actinobacteria                         hypothetical protein MMAR_2590 [Mycobacterium marinum M].  
118470413         LK-Nuclease+OST-HTH                                                               MSMEG_1210             289    Mycobacterium smegmatis str. MC2 155                            actinobacteria                         hypothetical protein MSMEG_1210 [Mycobacterium smegmatis str. MC2  
126433513         LK-Nuclease+OST-HTH                                                               Mjls_0904              311    Mycobacterium sp. JLS                                           actinobacteria                         hypothetical protein Mjls_0904 [Mycobacterium sp. JLS].  
108797873         LK-Nuclease+OST-HTH                                                               Mmcs_0898              311    Mycobacterium sp. MCS                                           actinobacteria                         hypothetical protein Mmcs_0898 [Mycobacterium sp. MCS].  
118618529         LK-Nuclease+OST-HTH                                                               MUL_3170               288    Mycobacterium ulcerans Agy99                                    actinobacteria                         hypothetical protein MUL_3170 [Mycobacterium ulcerans Agy99].  
120402029         LK-Nuclease+OST-HTH                                                               Mvan_1014              299    Mycobacterium vanbaalenii PYR-1                                 actinobacteria                         hypothetical protein Mvan_1014 [Mycobacterium vanbaalenii PYR-1].  
119714385         LK-Nuclease+OST-HTH                                                               Noca_0118              286    Nocardioides sp. JS614                                          actinobacteria                         hypothetical protein Noca_0118 [Nocardioides sp. JS614].  
108802863         LK-Nuclease+OST-HTH                                                               Rxyl_0007              487    Rubrobacter xylanophilus DSM 9941                               actinobacteria                         hypothetical protein Rxyl_0007 [Rubrobacter xylanophilus DSM 9941].  
257063827         LK-Nuclease+OST-HTH                                                               Shel_11150             314    Slackia heliotrinireducens DSM 20476                            actinobacteria                         hypothetical protein Shel_11150 [Slackia heliotrinireducens DSM  
269954862         LK-Nuclease+OST-HTH                                                               Xcel_0052              470    Xylanimonas cellulosilytica DSM 15894                           actinobacteria                         protein of unknown function LK-Nuclease [Xylanimonas cellulosilytica DSM  
126648210         LK-Nuclease+OST-HTH                                                               ALPR1_15829            256    Algoriphagus sp. PR1                                            bacteroidetes/chlorobi                 hypothetical protein ALPR1_15829 [Algoriphagus sp. PR1].  
153809371         LK-Nuclease+OST-HTH                                                               BACCAC_03685           192    Bacteroides caccae ATCC 43185                                   bacteroidetes/chlorobi                 hypothetical protein BACCAC_03685 [Bacteroides caccae ATCC 43185].  
154496234         LK-Nuclease+OST-HTH                                                               BACCAP_00519           288    Bacteroides capillosus ATCC 29799                               bacteroidetes/chlorobi                 hypothetical protein BACCAP_00519 [Bacteroides capillosus ATCC  
218128838         LK-Nuclease+OST-HTH                                                               BACEGG_00410           234    Bacteroides eggerthii DSM 20697                                 bacteroidetes/chlorobi                 hypothetical protein BACEGG_00410 [Bacteroides eggerthii DSM  
255012325         LK-Nuclease+OST-HTH                                                               B2_010100000320        240    Bacteroides sp. 2_1_7                                           bacteroidetes/chlorobi                 hypothetical protein B2_00320 [Bacteroides sp. 2_1_7].  
237708343         LK-Nuclease+OST-HTH                                                               BSBG_04258             234    Bacteroides sp. 9_1_42FAA                                       bacteroidetes/chlorobi                 conserved hypothetical protein [Bacteroides sp. 9_1_42FAA].  
167764633         LK-Nuclease+OST-HTH                                                               BACSTE_03023           234    Bacteroides stercoris ATCC 43183                                bacteroidetes/chlorobi                 hypothetical protein BACSTE_03023 [Bacteroides stercoris ATCC  
29348060          LK-Nuclease+OST-HTH                                                               BT_2650                233    Bacteroides thetaiotaomicron VPI-5482                           bacteroidetes/chlorobi                 hypothetical protein BT_2650 [Bacteroides thetaiotaomicron  
150004082         LK-Nuclease+OST-HTH                                                               BVU_1517               234    Bacteroides vulgatus ATCC 8482                                  bacteroidetes/chlorobi                 hypothetical protein BVU_1517 [Bacteroides vulgatus ATCC 8482].  
150003300         LK-Nuclease+OST-HTH                                                               BVU_0716               240    Bacteroides vulgatus ATCC 8482                                  bacteroidetes/chlorobi                 hypothetical protein BVU_0716 [Bacteroides vulgatus ATCC 8482].  
256419852         LK-Nuclease+OST-HTH                                                               Cpin_0806              246    Chitinophaga pinensis DSM 2588                                  bacteroidetes/chlorobi                 protein of unknown function LK-Nuclease [Chitinophaga pinensis DSM 2588].  
193212130         LK-Nuclease+OST-HTH                                                               Cpar_0461              450    Chlorobaculum parvum NCIB 8327                                  bacteroidetes/chlorobi                 protein of unknown function LK-Nuclease [Chlorobaculum parvum NCIB 8327].  
78188294          LK-Nuclease+OST-HTH                                                               Cag_0316               264    Chlorobium chlorochromatii CaD3                                 bacteroidetes/chlorobi                 hypothetical protein Cag_0316 [Chlorobium chlorochromatii CaD3].  
110596945         LK-Nuclease+OST-HTH                                                               CferDRAFT_2099         247    Chlorobium ferrooxidans DSM 13031                               bacteroidetes/chlorobi                 conserved hypothetical protein [Chlorobium ferrooxidans DSM 13031].  
189347523         LK-Nuclease+OST-HTH                                                               Clim_2044              249    Chlorobium limicola DSM 245                                     bacteroidetes/chlorobi                 conserved hypothetical protein [Chlorobium limicola DSM 245].  
78186223          LK-Nuclease+OST-HTH                                                               Plut_0335              247    Chlorobium luteolum DSM 273                                     bacteroidetes/chlorobi                 hypothetical protein Plut_0335 [Chlorobium luteolum DSM 273].  
189500992         LK-Nuclease+OST-HTH                                                               Cphamn1_2071           247    Chlorobium phaeobacteroides BS1                                 bacteroidetes/chlorobi                 conserved hypothetical protein [Chlorobium phaeobacteroides BS1].  
119357877         LK-Nuclease+OST-HTH                                                               Cpha266_2085           249    Chlorobium phaeobacteroides DSM 266                             bacteroidetes/chlorobi                 hypothetical protein Cpha266_2085 [Chlorobium phaeobacteroides DSM  
145219215         LK-Nuclease+OST-HTH                                                               Cvib_0400              247    Chlorobium phaeovibrioides DSM 265                              bacteroidetes/chlorobi                 hypothetical protein Cvib_0400 [Prosthecochloris vibrioformis DSM  
127511410         LK-Nuclease+OST-HTH                                                               Shew_0476              265    Shewanella loihica PV-4                                         proteobacteria>gammaproteobacteria     hypothetical protein Shew_0476 [Shewanella loihica PV-4].  
110637857         LK-Nuclease+OST-HTH                                                               CHU_1453               258    Cytophaga hutchinsonii ATCC 33406                               bacteroidetes/chlorobi                 hypothetical protein CHU_1453 [Cytophaga hutchinsonii ATCC 33406].  
255536314         LK-Nuclease+OST-HTH                                                               FIC_02187              246    Flavobacteriaceae bacterium 3519-10                             bacteroidetes/chlorobi                 Maebl [Flavobacteriaceae bacterium 3519-10].  
88714258          LK-Nuclease+OST-HTH                                                               FB2170_01831           255    Flavobacteriales bacterium HTCC2170                             bacteroidetes/chlorobi                 hypothetical protein FB2170_01831 [Flavobacteriales bacterium  
146300165         LK-Nuclease+OST-HTH                                                               Fjoh_2410              259    Flavobacterium johnsoniae UW101                                 bacteroidetes/chlorobi                 hypothetical protein Fjoh_2410 [Flavobacterium johnsoniae UW101].  
120436105         LK-Nuclease+OST-HTH                                                               GFO_1754               245    Gramella forsetii KT0803                                        bacteroidetes/chlorobi                 hypothetical protein GFO_1754 [Gramella forsetii KT0803].  
150008736         LK-Nuclease+OST-HTH                                                               BDI_2126               240    Parabacteroides distasonis ATCC 8503                            bacteroidetes/chlorobi                 hypothetical protein BDI_2126 [Parabacteroides distasonis ATCC  
194337430         LK-Nuclease+OST-HTH                                                               Ppha_2419              247    Pelodictyon phaeoclathratiforme BU-1                            bacteroidetes/chlorobi                 hypothetical protein Ppha_2419 [Pelodictyon phaeoclathratiforme  
258649060         LK-Nuclease+OST-HTH                                                               GCWU000325_02600       208    Prevotella tannerae ATCC 51259                                  bacteroidetes/chlorobi                 conserved hypothetical protein [Prevotella tannerae ATCC 51259].  
258649161         LK-Nuclease+OST-HTH                                                               GCWU000325_02739       239    Prevotella tannerae ATCC 51259                                  bacteroidetes/chlorobi                 conserved hypothetical protein [Prevotella tannerae ATCC 51259].  
194334672         LK-Nuclease+OST-HTH                                                               Paes_1873              252    Prosthecochloris aestuarii DSM 271                              bacteroidetes/chlorobi                 hypothetical protein Paes_1873 [Prosthecochloris aestuarii DSM  
227539037         LK-Nuclease+OST-HTH                                                               HMPREF0765_3281        259    Sphingobacterium spiritivorum ATCC 33300                        bacteroidetes/chlorobi                 protein of hypothetical function LK-Nuclease [Sphingobacterium  
241891367         LK-Nuclease+OST-HTH                                                               HMPREF0766_1204        276    Sphingobacterium spiritivorum ATCC 33861                        bacteroidetes/chlorobi                 protein of hypothetical function LK-Nuclease [Sphingobacterium  
171909553         LK-Nuclease+OST-HTH                                                               VspiD_010100000235     312    Verrucomicrobium spinosum DSM 4136                              chlamydiae/verrucomicrobia             hypothetical protein VspiD_00235 [Verrucomicrobium spinosum DSM  
187735246         LK-Nuclease+OST-HTH                                                               Amuc_0741              251    Akkermansia muciniphila ATCC BAA-835                            chlamydiae/verrucomicrobia             protein of unknown function LK-Nuclease [Akkermansia muciniphila ATCC  
225165594         LK-Nuclease+OST-HTH                                                               ObacDRAFT_5726         265    Opitutaceae bacterium TAV2                                      chlamydiae/verrucomicrobia             protein of unknown function LK-Nuclease [Opitutaceae bacterium TAV2].  
254443296         LK-Nuclease+OST-HTH                                                               VDG1235_1532           242    Verrucomicrobiae bacterium DG1235                               chlamydiae/verrucomicrobia             hypothetical protein VDG1235_1532 [Verrucomicrobiae bacterium  
182414934         LK-Nuclease+OST-HTH                                                               Oter_3120              264    Opitutus terrae PB90-1                                          chlamydiae/verrucomicrobia             hypothetical protein Oter_3120 [Opitutus terrae PB90-1].  
225165548         LK-Nuclease+OST-HTH                                                               ObacDRAFT_5777         280    Opitutaceae bacterium TAV2                                      chlamydiae/verrucomicrobia             conserved hypothetical protein [Opitutaceae bacterium TAV2].  
219848822         LK-Nuclease+OST-HTH                                                               Cagg_1924              334    Chloroflexus aggregans DSM 9485                                 chloroflexi                            protein of unknown function LK-Nuclease [Chloroflexus aggregans DSM  
16330259          LK-Nuclease+OST-HTH                                                               slr1870                249    Synechocystis sp. PCC 6803                                      cyanobacteria                          hypothetical protein slr1870 [Synechocystis sp. PCC 6803].  
254432426         LK-Nuclease+OST-HTH                                                               CPCC7001_2319          244    Cyanobium sp. PCC 7001                                          cyanobacteria                          protein containing LK-Nuclease [Cyanobium sp. PCC 7001].  
172035458         LK-Nuclease+OST-HTH                                                               cce_0542               235    Cyanothece sp. ATCC 51142                                       cyanobacteria                          hypothetical protein cce_0542 [Cyanothece sp. ATCC 51142].  
126656067         LK-Nuclease+OST-HTH                                                               CY0110_03254           235    Cyanothece sp. CCY0110                                          cyanobacteria                          hypothetical protein CY0110_03254 [Cyanothece sp. CCY0110].  
166364770         LK-Nuclease+OST-HTH                                                               MAE_20290              249    Microcystis aeruginosa NIES-843                                 cyanobacteria                          hypothetical protein MAE_20290 [Microcystis aeruginosa NIES-843].  
67924850          LK-Nuclease+OST-HTH                                                               CwatDRAFT_1822         235    Crocosphaera watsonii WH 8501                                   cyanobacteria                          hypothetical protein CwatDRAFT_1822 [Crocosphaera watsonii WH  
218245522         LK-Nuclease+OST-HTH                                                               PCC8801_0650           234    Cyanothece sp. PCC 8801                                         cyanobacteria                          protein of unknown function LK-Nuclease [Cyanothece sp. PCC 8801].  
261415964         LK-Nuclease+OST-HTH                                                               Fisuc_1570             384    Fibrobacter succinogenes subsp. succinogenes S85                fibrobacteres/acidobacteria            protein of unknown function LK-Nuclease [Fibrobacter succinogenes subsp.  
116626906         LK-Nuclease+OST-HTH                                                               Acid_7883              451    Candidatus Solibacter usitatus Ellin6076                        fibrobacteres/acidobacteria            hypothetical protein Acid_7883 [Solibacter usitatus Ellin6076].  
257453692         LK-Nuclease+OST-HTH                                                               ENHAE0001_0568         267    Enhydrobacter aerosaccus SK60                                   proteobacteria>gammaproteobacteria     protein containing LK-Nuclease [Enhydrobacter aerosaccus SK60].  
260775864         LK-Nuclease+OST-HTH                                                               VIC_001249             290    Vibrio coralliilyticus ATCC BAA-450                             proteobacteria>gammaproteobacteria     hypothetical protein VIC_001249 [Vibrio coralliilyticus ATCC  
260774799         LK-Nuclease+OST-HTH                                                               VIC_000167             259    Vibrio coralliilyticus ATCC BAA-450                             proteobacteria>gammaproteobacteria     hypothetical protein VIC_000167 [Vibrio coralliilyticus ATCC  
153954651         LK-Nuclease+OST-HTH                                                               CKL_2033               250    Clostridium kluyveri DSM 555                                    firmicutes                             hypothetical protein CKL_2033 [Clostridium kluyveri DSM 555].  
150389764         LK-Nuclease+OST-HTH                                                               Amet_1991              250    Alkaliphilus metalliredigens QYMF                               firmicutes                             hypothetical protein Amet_1991 [Alkaliphilus metalliredigens QYMF].  
89894266          LK-Nuclease+OST-HTH                                                               DSY1520                253    Desulfitobacterium hafniense Y51                                firmicutes                             hypothetical protein DSY1520 [Desulfitobacterium hafniense Y51].  
167770975         LK-Nuclease+OST-HTH                                                               ANACOL_02329           286    Anaerotruncus colihominis DSM 17241                             firmicutes                             hypothetical protein ANACOL_02329 [Anaerotruncus colihominis DSM  
225568810         LK-Nuclease+OST-HTH                                                               CLOHYLEM_04889         313    Clostridium hylemonae DSM 15053                                 firmicutes                             hypothetical protein CLOHYLEM_04889 [Clostridium hylemonae DSM  
197301939         LK-Nuclease+OST-HTH                                                               RUMLAC_00661           322    Ruminococcus lactaris ATCC 29176                                firmicutes                             hypothetical protein RUMLAC_00661 [Ruminococcus lactaris ATCC  
51102893          LK-Nuclease+OST-HTH                                                               -                      280    Pseudomonas viridiflava                                         proteobacteria>gammaproteobacteria     hypothetical protein [Pseudomonas viridiflava].  
259504812         LK-Nuclease+OST-HTH                                                               HMPREF0357_0884        241    Erysipelothrix rhusiopathiae ATCC 19414                         firmicutes                             conserved hypothetical protein [Erysipelothrix rhusiopathiae ATCC  
254524406         LK-Nuclease+OST-HTH                                                               SSKA14_3544            259    Stenotrophomonas sp. SKA14                                      proteobacteria>gammaproteobacteria     protein containing LK-Nuclease [Stenotrophomonas sp. SKA14].  
167760471         LK-Nuclease+OST-HTH                                                               CLOSCI_02845           311    Clostridium scindens ATCC 35704                                 firmicutes                             hypothetical protein CLOSCI_02845 [Clostridium scindens ATCC  
256756430         LK-Nuclease+OST-HTH                                                               CpapDRAFT_3358         258    Clostridium papyrosolvens DSM 2782                              firmicutes                             protein of unknown function LK-Nuclease [Clostridium papyrosolvens DSM  
225175633         LK-Nuclease+OST-HTH                                                               DealDRAFT_1482         246    Dethiobacter alkaliphilus AHT 1                                 firmicutes                             conserved hypothetical protein [Dethiobacter alkaliphilus AHT 1].  
255280264         LK-Nuclease+OST-HTH                                                               BRYFOR_05595           349    Bryantella formatexigens DSM 14469                              firmicutes                             conserved hypothetical protein [Bryantella formatexigens DSM  
153854120         LK-Nuclease+OST-HTH                                                               DORLON_01419           309    Dorea longicatena DSM 13814                                     firmicutes                             hypothetical protein DORLON_01419 [Dorea longicatena DSM 13814].  
254225637         LK-Nuclease+OST-HTH                                                               VCV51_A0361            259    Vibrio cholerae V51                                             proteobacteria>gammaproteobacteria     protein of unknown function [Vibrio cholerae V51].  
194367375         LK-Nuclease+OST-HTH                                                               Smal_3603              259    Stenotrophomonas maltophilia R551-3                             proteobacteria>gammaproteobacteria     protein of unknown function LK-Nuclease [Stenotrophomonas maltophilia  
166033313         LK-Nuclease+OST-HTH                                                               DORFOR_03039           308    Dorea formicigenerans ATCC 27755                                firmicutes                             hypothetical protein DORFOR_03039 [Dorea formicigenerans ATCC  
190576025         LK-Nuclease+OST-HTH                                                               Smlt4196               259    Stenotrophomonas maltophilia K279a                              proteobacteria>gammaproteobacteria     conserved hypothetical protein [Stenotrophomonas maltophilia  
261367119         LK-Nuclease+OST-HTH                                                               SUBVAR_05158           275    Subdoligranulum variabile DSM 15176                             firmicutes                             conserved hypothetical protein [Subdoligranulum variabile DSM  
51102938          LK-Nuclease+OST-HTH                                                               -                      280    Pseudomonas viridiflava                                         proteobacteria>gammaproteobacteria     hypothetical protein [Pseudomonas viridiflava].  
229825159         LK-Nuclease+OST-HTH                                                               GCWU000182_00510       249    Abiotrophia defectiva ATCC 49176                                firmicutes                             hypothetical protein GCWU000182_00510 [Abiotrophia defectiva ATCC  
167746693         LK-Nuclease+OST-HTH                                                               ANACAC_01404           351    Anaerostipes caccae DSM 14662                                   firmicutes                             hypothetical protein ANACAC_01404 [Anaerostipes caccae DSM 14662].  
21233195          LK-Nuclease+OST-HTH                                                               XCC3767                277    Xanthomonas campestris pv. campestris str. ATCC 33913           proteobacteria>gammaproteobacteria     hypothetical protein XCC3767 [Xanthomonas campestris pv. campestris  
220928157         LK-Nuclease+OST-HTH                                                               Ccel_0707              258    Clostridium cellulolyticum H10                                  firmicutes                             protein of unknown function LK-Nuclease [Clostridium cellulolyticum H10].  
227824978         LK-Nuclease+OST-HTH                                                               ACDG_01754             262    Acidaminococcus sp. D21                                         firmicutes                             conserved hypothetical protein [Acidaminococcus sp. D21].  
225018409         LK-Nuclease+OST-HTH                                                               CLOSTMETH_02356        246    Clostridium methylpentosum DSM 5476                             firmicutes                             hypothetical protein CLOSTMETH_02356 [Clostridium methylpentosum  
32473273          LK-Nuclease+OST-HTH                                                               RB4721                 242    Rhodopirellula baltica SH 1                                     planctomycetes                         hypothetical protein RB4721 [Rhodopirellula baltica SH 1].  
114778764         LK-Nuclease+OST-HTH                                                               SPV1_03033             277    Mariprofundus ferrooxydans PV-1                                 proteobacteria                         hypothetical protein SPV1_03033 [Mariprofundus ferrooxydans PV-1].  
115526363         LK-Nuclease+OST-HTH                                                               RPE_4370               253    Rhodopseudomonas palustris BisA53                               proteobacteria>alphaproteobacteria     hypothetical protein RPE_4370 [Rhodopseudomonas palustris BisA53].  
163746469         LK-Nuclease+OST-HTH                                                               OIHEL45_13730          232    Oceanibulbus indolifex HEL-45                                   proteobacteria>alphaproteobacteria     hypothetical protein OIHEL45_13730 [Oceanibulbus indolifex HEL-45].  
92115812          LK-Nuclease+OST-HTH                                                               Nham_0180              254    Nitrobacter hamburgensis X14                                    proteobacteria>alphaproteobacteria     hypothetical protein Nham_0180 [Nitrobacter hamburgensis X14].  
94496412          LK-Nuclease+OST-HTH                                                               SKA58_09476            257    Sphingomonas sp. SKA58                                          proteobacteria>alphaproteobacteria     hypothetical protein SKA58_09476 [Sphingomonas sp. SKA58].  
259419136         LK-Nuclease+OST-HTH                                                               SCH4B_3972             258    Silicibacter sp. TrichCH4B                                      proteobacteria>alphaproteobacteria     conserved hypothetical protein [Silicibacter sp. TrichCH4B].  
84515798          LK-Nuclease+OST-HTH                                                               SKA53_14151            246    Loktanella vestfoldensis SKA53                                  proteobacteria>alphaproteobacteria     hypothetical protein SKA53_14151 [Loktanella vestfoldensis SKA53].  
77461998          LK-Nuclease+OST-HTH                                                               RSP_1459               248    Rhodobacter sphaeroides 2.4.1                                   proteobacteria>alphaproteobacteria     hypothetical protein RSP_1459 [Rhodobacter sphaeroides 2.4.1].  
148550669         LK-Nuclease+OST-HTH                                                               Swit_5232              284    Sphingomonas wittichii RW1                                      proteobacteria>alphaproteobacteria     hypothetical protein Swit_5232 [Sphingomonas wittichii RW1].  
149915348         LK-Nuclease+OST-HTH                                                               RAZWK3B_05017          232    Roseobacter sp. AzwK-3b                                         proteobacteria>alphaproteobacteria     predicted S-transferase [Roseobacter sp. AzwK-3b].  
254439173         LK-Nuclease+OST-HTH                                                               OA307_4043             233    Octadecabacter antarcticus 307                                  proteobacteria>alphaproteobacteria     conserved hypothetical protein [Octadecabacter antarcticus 307].  
153011951         LK-Nuclease+OST-HTH                                                               Oant_4745              246    Ochrobactrum anthropi ATCC 49188                                proteobacteria>alphaproteobacteria     hypothetical protein Oant_4745 [Ochrobactrum anthropi ATCC 49188].  
85374093          LK-Nuclease+OST-HTH                                                               ELI_06330              245    Erythrobacter litoralis HTCC2594                                proteobacteria>alphaproteobacteria     hypothetical protein ELI_06330 [Erythrobacter litoralis HTCC2594].  
254464182         LK-Nuclease+OST-HTH                                                               RBY4I_782              245    Rhodobacterales bacterium Y4I                                   proteobacteria>alphaproteobacteria     protein containing LK-Nuclease [Rhodobacterales bacterium Y4I].  
126729949         LK-Nuclease+OST-HTH                                                               SSE37_16263            233    Sagittula stellata E-37                                         proteobacteria>alphaproteobacteria     hypothetical protein SSE37_16263 [Sagittula stellata E-37].  
146276115         LK-Nuclease+OST-HTH                                                               Rsph17025_0057         246    Rhodobacter sphaeroides ATCC 17025                              proteobacteria>alphaproteobacteria     hypothetical protein Rsph17025_0057 [Rhodobacter sphaeroides ATCC  
241554076         LK-Nuclease+OST-HTH                                                               Rleg_6293              262    Rhizobium leguminosarum bv. trifolii WSM1325                    proteobacteria>alphaproteobacteria     protein of unknown function LK-Nuclease [Rhizobium leguminosarum bv.  
99080500          LK-Nuclease+OST-HTH                                                               TM1040_0659            262    Ruegeria sp. TM1040                                             proteobacteria>alphaproteobacteria     hypothetical protein TM1040_0659 [Ruegeria sp. TM1040].  
89052539          LK-Nuclease+OST-HTH                                                               Jann_0048              239    Jannaschia sp. CCS1                                             proteobacteria>alphaproteobacteria     hypothetical protein Jann_0048 [Jannaschia sp. CCS1].  
148255393         LK-Nuclease+OST-HTH                                                               BBta_4005              263    Bradyrhizobium sp. BTAi1                                        proteobacteria>alphaproteobacteria     hypothetical protein BBta_4005 [Bradyrhizobium sp. BTAi1].  
146340536         LK-Nuclease+OST-HTH                                                               BRADO3579              263    Bradyrhizobium sp. ORS278                                       proteobacteria>alphaproteobacteria     hypothetical protein BRADO3579 [Bradyrhizobium sp. ORS278].  
149184574         LK-Nuclease+OST-HTH                                                               ED21_27688             241    Erythrobacter sp. SD-21                                         proteobacteria>alphaproteobacteria     hypothetical protein ED21_27688 [Erythrobacter sp. SD-21].  
52220908          LK-Nuclease+OST-HTH                                                               pAgK84_22              275    Agrobacterium tumefaciens                                       proteobacteria>alphaproteobacteria     hypothetical protein pAgK84_22 [Agrobacterium tumefaciens].  
85708688          LK-Nuclease+OST-HTH                                                               NAP1_05595             240    Erythrobacter sp. NAP1                                          proteobacteria>alphaproteobacteria     hypothetical protein NAP1_05595 [Erythrobacter sp. NAP1].  
260574190         LK-Nuclease+OST-HTH                                                               Rsw2DRAFT_0182         252    Rhodobacter sp. SW2                                             proteobacteria>alphaproteobacteria     protein of unknown function LK-Nuclease [Rhodobacter sp. SW2].  
27378275          LK-Nuclease+OST-HTH                                                               blr3164                265    Bradyrhizobium japonicum USDA 110                               proteobacteria>alphaproteobacteria     hypothetical protein blr3164 [Bradyrhizobium japonicum USDA 110].  
84684164          LK-Nuclease+OST-HTH                                                               RB2654_16986           234    Rhodobacterales bacterium HTCC2654                              proteobacteria>alphaproteobacteria     hypothetical protein RB2654_16986 [Rhodobacterales bacterium  
283841317         LK-Nuclease+OST-HTH                                                               Rpdx1DRAFT_2339        266    Rhodopseudomonas palustris DX-1                                 proteobacteria>alphaproteobacteria     protein of unknown function LK-Nuclease [Rhodopseudomonas palustris  
89070045          LK-Nuclease+OST-HTH                                                               OG2516_09755           238    Oceanicola granulosus HTCC2516                                  proteobacteria>alphaproteobacteria     hypothetical protein OG2516_09755 [Oceanicola granulosus HTCC2516].  
221640949         LK-Nuclease+OST-HTH                                                               RSKD131_2850           238    Rhodobacter sphaeroides KD131                                   proteobacteria>alphaproteobacteria     hypothetical protein RSKD131_2850 [Rhodobacter sphaeroides KD131].  
83949890          LK-Nuclease+OST-HTH                                                               ISM_02310              231    Roseovarius nubinhibens ISM                                     proteobacteria>alphaproteobacteria     hypothetical protein ISM_02310 [Roseovarius nubinhibens ISM].  
260428850         LK-Nuclease+OST-HTH                                                               CSE45_3613             236    Citreicella sp. SE45                                            proteobacteria>alphaproteobacteria     conserved hypothetical protein [Citreicella sp. SE45].  
192292848         LK-Nuclease+OST-HTH                                                               Rpal_4482              267    Rhodopseudomonas palustris TIE-1                                proteobacteria>alphaproteobacteria     conserved hypothetical protein [Rhodopseudomonas palustris TIE-1].  
39937021          LK-Nuclease+OST-HTH                                                               RPA3960                267    Rhodopseudomonas palustris CGA009                               proteobacteria>alphaproteobacteria     hypothetical protein RPA3960 [Rhodopseudomonas palustris CGA009].  
159042661         LK-Nuclease+OST-HTH                                                               Dshi_0105              236    Dinoroseobacter shibae DFL 12                                   proteobacteria>alphaproteobacteria     hypothetical protein Dshi_0105 [Dinoroseobacter shibae DFL 12].  
90418798          LK-Nuclease+OST-HTH                                                               SI859A1_00703          273    Aurantimonas manganoxydans SI85-9A1                             proteobacteria>alphaproteobacteria     conserved hypothetical protein [Aurantimonas manganoxydans  
260432079         LK-Nuclease+OST-HTH                                                               SL1157_1203            238    Silicibacter lacuscaerulensis ITI-1157                          proteobacteria>alphaproteobacteria     protein containing LK-Nuclease [Silicibacter lacuscaerulensis ITI-1157].  
84500442          LK-Nuclease+OST-HTH                                                               OB2597_10806           236    Oceanicola batsensis HTCC2597                                   proteobacteria>alphaproteobacteria     hypothetical protein OB2597_10806 [Oceanicola batsensis HTCC2597].  
114764063         LK-Nuclease+OST-HTH                                                               R2601_15432            236    Roseovarius sp. HTCC2601                                        proteobacteria>alphaproteobacteria     hypothetical protein R2601_15432 [Roseovarius sp. HTCC2601].  
75674368          LK-Nuclease+OST-HTH                                                               Nwi_0169               271    Nitrobacter winogradskyi Nb-255                                 proteobacteria>alphaproteobacteria     hypothetical protein Nwi_0169 [Nitrobacter winogradskyi Nb-255].  
87200042          LK-Nuclease+OST-HTH                                                               Saro_2026              270    Novosphingobium aromaticivorans DSM 12444                       proteobacteria>alphaproteobacteria     hypothetical protein Saro_2026 [Novosphingobium aromaticivorans DSM  
30248674          LK-Nuclease+OST-HTH                                                               NE0665                 269    Nitrosomonas europaea ATCC 19718                                proteobacteria>betaproteobacteria      hypothetical protein NE0665 [Nitrosomonas europaea ATCC 19718].  
257094155         LK-Nuclease+OST-HTH                                                               CAP2UW1_2580           311    Candidatus Accumulibacter phosphatis clade IIA str. UW-1        proteobacteria>betaproteobacteria      hypothetical protein CAP2UW1_2580 [Candidatus Accumulibacter  
152983068         LK-Nuclease+OST-HTH                                                               mma_1010               236    Janthinobacterium sp. Marseille                                 proteobacteria>betaproteobacteria      hypothetical protein mma_1010 [Janthinobacterium sp. Marseille].  
114330619         LK-Nuclease+OST-HTH                                                               Neut_0604              268    Nitrosomonas eutropha C91                                       proteobacteria>betaproteobacteria      hypothetical protein Neut_0604 [Nitrosomonas eutropha C91].  
89900334          LK-Nuclease+OST-HTH                                                               Rfer_1542              272    Rhodoferax ferrireducens T118                                   proteobacteria>betaproteobacteria      hypothetical protein Rfer_1542 [Rhodoferax ferrireducens T118].  
121604991         LK-Nuclease+OST-HTH                                                               Pnap_2090              268    Polaromonas naphthalenivorans CJ2                               proteobacteria>betaproteobacteria      hypothetical protein Pnap_2090 [Polaromonas naphthalenivorans CJ2].  
161525283         LK-Nuclease+OST-HTH                                                               Bmul_2113              508    Burkholderia multivorans ATCC 17616                             proteobacteria>betaproteobacteria      hypothetical protein Bmul_2113 [Burkholderia multivorans ATCC  
71905737          LK-Nuclease+OST-HTH                                                               Daro_0095              273    Dechloromonas aromatica RCB                                     proteobacteria>betaproteobacteria      hypothetical protein Daro_0095 [Dechloromonas aromatica RCB].  
74317958          LK-Nuclease+OST-HTH                                                               Tbd_1940               308    Thiobacillus denitrificans ATCC 25259                           proteobacteria>betaproteobacteria      hypothetical protein Tbd_1940 [Thiobacillus denitrificans ATCC  
193222199         LK-Nuclease+OST-HTH                                                               HEAR0113               267    Herminiimonas arsenicoxydans                                    proteobacteria>betaproteobacteria      Conserved hypothetical protein [Herminiimonas arsenicoxydans].  
56477068          LK-Nuclease+OST-HTH                                                               ebA2897                510    Aromatoleum aromaticum EbN1                                     proteobacteria>betaproteobacteria      hypothetical protein ebA2897 [Aromatoleum aromaticum EbN1].  
272528892         LK-Nuclease+OST-HTH                                                               BC1002DRAFT_1216       515    Burkholderia sp. CCGE1002                                       proteobacteria>betaproteobacteria      protein of unknown function LK-Nuclease [Burkholderia sp. CCGE1002].  
115351126         LK-Nuclease+OST-HTH                                                               Bamb_1072              507    Burkholderia ambifaria AMMD                                     proteobacteria>betaproteobacteria      hypothetical protein Bamb_1072 [Burkholderia ambifaria AMMD].  
167566002         LK-Nuclease+OST-HTH                                                               BoklE_010100025814     524    Burkholderia oklahomensis EO147                                 proteobacteria>betaproteobacteria      hypothetical protein BoklE_25814 [Burkholderia oklahomensis EO147].  
253998702         LK-Nuclease+OST-HTH                                                               Msip34_0991            275    Methylovorus sp. SIP3-4                                         proteobacteria>betaproteobacteria      protein of unknown function LK-Nuclease [Methylovorus sp. SIP3-4].  
113869183         LK-Nuclease+OST-HTH                                                               h16_A3229              265    Ralstonia eutropha H16                                          proteobacteria>betaproteobacteria      hypothetical protein H16_A3229 [Ralstonia eutropha H16].  
282885100         LK-Nuclease+OST-HTH                                                               BC1001DRAFT_1691       534    Burkholderia sp. CCGE1001                                       proteobacteria>betaproteobacteria      protein of unknown function LK-Nuclease [Burkholderia sp. CCGE1001].  
241766896         LK-Nuclease+OST-HTH                                                               AcdelDRAFT_3936        316    Acidovorax delafieldii 2AN                                      proteobacteria>betaproteobacteria      protein of unknown function LK-Nuclease [Acidovorax delafieldii 2AN].  
134295221         LK-Nuclease+OST-HTH                                                               Bcep1808_1110          507    Burkholderia vietnamiensis G4                                   proteobacteria>betaproteobacteria      hypothetical protein Bcep1808_1110 [Burkholderia vietnamiensis G4].  
209517483         LK-Nuclease+OST-HTH                                                               BH160DRAFT_2601        505    Burkholderia sp. H160                                           proteobacteria>betaproteobacteria      protein of unknown function LK-Nuclease [Burkholderia sp. H160].  
172060127         LK-Nuclease+OST-HTH                                                               BamMC406_1072          501    Burkholderia ambifaria MC40-6                                   proteobacteria>betaproteobacteria      hypothetical protein BamMC406_1072 [Burkholderia ambifaria MC40-6].  
91776174          LK-Nuclease+OST-HTH                                                               Mfla_1822              275    Methylobacillus flagellatus KT                                  proteobacteria>betaproteobacteria      hypothetical protein Mfla_1822 [Methylobacillus flagellatus KT].  
217418449         LK-Nuclease+OST-HTH                                                               BUC_6042               500    Burkholderia pseudomallei 576                                   proteobacteria>betaproteobacteria      conserved hypothetical protein [Burkholderia pseudomallei 576].  
170693531         LK-Nuclease+OST-HTH                                                               BgramDRAFT_3500        534    Burkholderia graminis C4D1M                                     proteobacteria>betaproteobacteria      protein of unknown function LK-Nuclease [Burkholderia graminis C4D1M].  
254245848         LK-Nuclease+OST-HTH                                                               BCPG_00568             500    Burkholderia cenocepacia PC184                                  proteobacteria>betaproteobacteria      hypothetical protein BCPG_00568 [Burkholderia cenocepacia PC184].  
238024602         LK-Nuclease+OST-HTH                                                               bglu_2g12170           538    Burkholderia glumae BGR1                                        proteobacteria>betaproteobacteria      hypothetical protein bglu_2g12170 [Burkholderia glumae BGR1].  
217970117         LK-Nuclease+OST-HTH                                                               Tmz1t_1700             564    Thauera sp. MZ1T                                                proteobacteria>betaproteobacteria      protein of unknown function LK-Nuclease [Thauera sp. MZ1T].  
134282577         LK-Nuclease+OST-HTH                                                               BURPS305_1600          622    Burkholderia pseudomallei 305                                   proteobacteria>betaproteobacteria      conserved hypothetical protein [Burkholderia pseudomallei 305].  
255259391         LK-Nuclease+OST-HTH                                                               SlitDRAFT_1205         274    Sideroxydans lithotrophicus ES-1                                proteobacteria>betaproteobacteria      protein of unknown function LK-Nuclease [Sideroxydans lithotrophicus  
107022270         LK-Nuclease+OST-HTH                                                               Bcen_0714              498    Burkholderia cenocepacia AU 1054                                proteobacteria>betaproteobacteria      hypothetical protein Bcen_0714 [Burkholderia cenocepacia AU 1054].  
160896833         LK-Nuclease+OST-HTH                                                               Daci_1386              303    Delftia acidovorans SPH-1                                       proteobacteria>betaproteobacteria      hypothetical protein Daci_1386 [Delftia acidovorans SPH-1].  
121583279         LK-Nuclease+OST-HTH                                                               Pnap_4909              279    Polaromonas naphthalenivorans CJ2                               proteobacteria>betaproteobacteria      hypothetical protein Pnap_4909 [Polaromonas naphthalenivorans CJ2].  
170732518         LK-Nuclease+OST-HTH                                                               Bcenmc03_1168          498    Burkholderia cenocepacia MC0-3                                  proteobacteria>betaproteobacteria      hypothetical protein Bcenmc03_1168 [Burkholderia cenocepacia  
221065080         LK-Nuclease+OST-HTH                                                               CtesDRAFT_PD0417       300    Comamonas testosteroni KF-1                                     proteobacteria>betaproteobacteria      protein of unknown function LK-Nuclease [Comamonas testosteroni KF-1].  
91786924          LK-Nuclease+OST-HTH                                                               Bpro_1025              260    Polaromonas sp. JS666                                           proteobacteria>betaproteobacteria      hypothetical protein Bpro_1025 [Polaromonas sp. JS666].  
78065774          LK-Nuclease+OST-HTH                                                               Bcep18194_A4302        496    Burkholderia sp. 383                                            proteobacteria>betaproteobacteria      hypothetical protein Bcep18194_A4302 [Burkholderia sp. 383].  
82703323          LK-Nuclease+OST-HTH                                                               Nmul_A2205             381    Nitrosospira multiformis ATCC 25196                             proteobacteria>betaproteobacteria      hypothetical protein Nmul_A2205 [Nitrosospira multiformis ATCC  
163857253         LK-Nuclease+OST-HTH                                                               Bpet2940               385    Bordetella petrii DSM 12804                                     proteobacteria>betaproteobacteria      hypothetical protein Bpet2940 [Bordetella petrii DSM 12804].  
188993344         LK-Nuclease+OST-HTH                                                               xccb100_3949           277    Xanthomonas campestris pv. campestris str. B100                 proteobacteria>gammaproteobacteria     hypothetical protein xccb100_3949 [Xanthomonas campestris pv.  
237808105         LK-Nuclease+OST-HTH                                                               Tola_1344              277    Tolumonas auensis DSM 9187                                      proteobacteria>gammaproteobacteria     protein of unknown function LK-Nuclease [Tolumonas auensis DSM 9187].  
206559459         LK-Nuclease+OST-HTH                                                               BCAL1072               491    Burkholderia cenocepacia J2315                                  proteobacteria>betaproteobacteria      hypothetical protein BCAL1072 [Burkholderia cenocepacia J2315].  
226198540         LK-Nuclease+OST-HTH                                                               BUH_6138               486    Burkholderia pseudomallei Pakistan 9                            proteobacteria>betaproteobacteria      conserved hypothetical protein [Burkholderia pseudomallei Pakistan  
76819463          LK-Nuclease+OST-HTH                                                               BURPS1710b_A0347       483    Burkholderia pseudomallei 1710b                                 proteobacteria>betaproteobacteria      hypothetical protein BURPS1710b_A0347 [Burkholderia pseudomallei  
84625703          LK-Nuclease+OST-HTH                                                               XOO_4046               276    Xanthomonas oryzae pv. oryzae MAFF 311018                       proteobacteria>gammaproteobacteria     hypothetical protein XOO_4046 [Xanthomonas oryzae pv. oryzae MAFF  
167589253         LK-Nuclease+OST-HTH                                                               BuboB_010100028211     280    Burkholderia ubonensis Bu                                       proteobacteria>betaproteobacteria      hypothetical protein BuboB_28211 [Burkholderia ubonensis Bu].  
120612954         LK-Nuclease+OST-HTH                                                               Aave_4318              298    Acidovorax citrulli AAC00-1                                     proteobacteria>betaproteobacteria      hypothetical protein Aave_4318 [Acidovorax avenae subsp. citrulli  
113869484         LK-Nuclease+OST-HTH                                                               h16_A3546              417    Ralstonia eutropha H16                                          proteobacteria>betaproteobacteria      hypothetical protein H16_A3546 [Ralstonia eutropha H16].  
73542916          LK-Nuclease+OST-HTH                                                               Reut_A3232             419    Ralstonia eutropha JMP134                                       proteobacteria>betaproteobacteria      hypothetical protein Reut_A3232 [Ralstonia eutropha JMP134].  
256758405         LK-Nuclease+OST-HTH                                                               TintDRAFT_1614         259    Thiomonas intermedia K12                                        proteobacteria>betaproteobacteria      protein of unknown function LK-Nuclease [Thiomonas intermedia K12].  
222112194         LK-Nuclease+OST-HTH                                                               Dtpsy_3025             285    Diaphorobacter sp. TPSY                                         proteobacteria>betaproteobacteria      protein of unknown function LK-Nuclease [Diaphorobacter sp. TPSY].  
71908802          LK-Nuclease+OST-HTH                                                               Daro_3189              421    Dechloromonas aromatica RCB                                     proteobacteria>betaproteobacteria      hypothetical protein Daro_3189 [Dechloromonas aromatica RCB].  
194291074         LK-Nuclease+OST-HTH                                                               RALTA_A2999            426    Cupriavidus taiwanensis                                         proteobacteria>betaproteobacteria      hypothetical protein RALTA_A2999 [Cupriavidus taiwanensis].  
224827245         LK-Nuclease+OST-HTH                                                               FuraDRAFT_3843         258    Lutiella nitroferrum 2002                                       proteobacteria>betaproteobacteria      protein of unknown function LK-Nuclease [Lutiella nitroferrum 2002].  
238023177         LK-Nuclease+OST-HTH                                                               GCWU000324_03103       258    Kingella oralis ATCC 51147                                      proteobacteria>betaproteobacteria      hypothetical protein GCWU000324_03103 [Kingella oralis ATCC 51147].  
94312326          LK-Nuclease+OST-HTH                                                               Rmet_3395              432    Ralstonia metallidurans CH34                                    proteobacteria>betaproteobacteria      hypothetical protein Rmet_3395 [Ralstonia metallidurans CH34].  
270495010         LK-Nuclease+OST-HTH                                                               AcavDRAFT_3796         298    Acidovorax avenae subsp. avenae ATCC 19860                      proteobacteria>betaproteobacteria      protein of unknown function LK-Nuclease [Acidovorax avenae subsp. avenae  
34495828          LK-Nuclease+OST-HTH                                                               CV_0373                297    Chromobacterium violaceum ATCC 12472                            proteobacteria>betaproteobacteria      hypothetical protein CV_0373 [Chromobacterium violaceum ATCC  
126456340         LK-Nuclease+OST-HTH                                                               BURPS1106A_A1798       483    Burkholderia pseudomallei 1106a                                 proteobacteria>betaproteobacteria      hypothetical protein BURPS1106A_A1798 [Burkholderia pseudomallei  
186475309         LK-Nuclease+OST-HTH                                                               Bphy_0541              440    Burkholderia phymatum STM815                                    proteobacteria>betaproteobacteria      hypothetical protein Bphy_0541 [Burkholderia phymatum STM815].  
167839964         LK-Nuclease+OST-HTH                                                               Bpse38_010100025044    445    Burkholderia thailandensis MSMB43                               proteobacteria>betaproteobacteria      hypothetical protein Bpse38_25044 [Burkholderia thailandensis  
257094026         LK-Nuclease+OST-HTH                                                               CAP2UW1_2449           460    Candidatus Accumulibacter phosphatis clade IIA str. UW-1        proteobacteria>betaproteobacteria      protein of unknown function LK-Nuclease [Candidatus Accumulibacter  
121596033         LK-Nuclease+OST-HTH                                                               Ajs_3744               287    Acidovorax sp. JS42                                             proteobacteria>betaproteobacteria      hypothetical protein Ajs_3744 [Acidovorax sp. JS42].  
167577696         LK-Nuclease+OST-HTH                                                               BthaT_010100006146     463    Burkholderia thailandensis TXDOH                                proteobacteria>betaproteobacteria      hypothetical protein BthaT_06146 [Burkholderia thailandensis  
119898953         LK-Nuclease+OST-HTH                                                               azo2662                481    Azoarcus sp. BH72                                               proteobacteria>betaproteobacteria      hypothetical protein azo2662 [Azoarcus sp. BH72].  
91785005          LK-Nuclease+OST-HTH                                                               Bxe_A0775              480    Burkholderia xenovorans LB400                                   proteobacteria>betaproteobacteria      hypothetical protein Bxe_A0775 [Burkholderia xenovorans LB400].  
167573079         LK-Nuclease+OST-HTH                                                               BoklC_010100024798     468    Burkholderia oklahomensis C6786                                 proteobacteria>betaproteobacteria      hypothetical protein BoklC_24798 [Burkholderia oklahomensis C6786].  
264680306         LK-Nuclease+OST-HTH                                                               CtCNB1_4174            293    Comamonas testosteroni CNB-2                                    proteobacteria>betaproteobacteria      hypothetical protein CtCNB1_4174 [Comamonas testosteroni CNB-2].  
167828427         LK-Nuclease+OST-HTH                                                               Bpseu9_010100032389    469    Burkholderia pseudomallei 9                                     proteobacteria>betaproteobacteria      hypothetical protein Bpseu9_32389 [Burkholderia pseudomallei 9].  
237509073         LK-Nuclease+OST-HTH                                                               GBP346_B1125           480    Burkholderia pseudomallei MSHR346                               proteobacteria>betaproteobacteria      conserved hypothetical protein [Burkholderia pseudomallei MSHR346].  
221201416         LK-Nuclease+OST-HTH                                                               BURMUCGD2M_2650        479    Burkholderia multivorans CGD2M                                  proteobacteria>betaproteobacteria      protein of unknown function LK-Nuclease [Burkholderia multivorans CGD2M].  
255063447         LK-Nuclease+OST-HTH                                                               NAL212DRAFT_1617       292    Nitrosomonas sp. AL212                                          proteobacteria>betaproteobacteria      protein of unknown function LK-Nuclease [Nitrosomonas sp. AL212].  
221214025         LK-Nuclease+OST-HTH                                                               BURMUCGD1_2247         479    Burkholderia multivorans CGD1                                   proteobacteria>betaproteobacteria      protein of unknown function LK-Nuclease [Burkholderia multivorans CGD1].  
83717296          LK-Nuclease+OST-HTH                                                               BTH_II1099             472    Burkholderia thailandensis E264                                 proteobacteria>betaproteobacteria      hypothetical protein BTH_II1099 [Burkholderia thailandensis E264].  
171317829         LK-Nuclease+OST-HTH                                                               BamMEX5DRAFT_2362      643    Burkholderia ambifaria MEX-5                                    proteobacteria>betaproteobacteria      protein of unknown function LK-Nuclease [Burkholderia ambifaria MEX-5].  
254183703         LK-Nuclease+OST-HTH                                                               BURPS1655_I0930        474    Burkholderia pseudomallei 1655                                  proteobacteria>betaproteobacteria      conserved hypothetical protein [Burkholderia pseudomallei 1655].  
167915186         LK-Nuclease+OST-HTH                                                               Bpse112_010100032216   474    Burkholderia pseudomallei 112                                   proteobacteria>betaproteobacteria      hypothetical protein Bpse112_32216 [Burkholderia pseudomallei 112].  
167723901         LK-Nuclease+OST-HTH                                                               BpseD_010100033095     474    Burkholderia pseudomallei DM98                                  proteobacteria>betaproteobacteria      hypothetical protein BpseD_33095 [Burkholderia pseudomallei DM98].  
254174034         LK-Nuclease+OST-HTH                                                               BMA10399_0821          477    Burkholderia mallei ATCC 10399                                  proteobacteria>betaproteobacteria      protein of unknown function [Burkholderia mallei ATCC 10399].  
167923021         LK-Nuclease+OST-HTH                                                               BpseBC_010100030982    477    Burkholderia pseudomallei BCC215                                proteobacteria>betaproteobacteria      hypothetical protein BpseBC_30982 [Burkholderia pseudomallei  
187925165         LK-Nuclease+OST-HTH                                                               Bphyt_3191             478    Burkholderia phytofirmans PsJN                                  proteobacteria>betaproteobacteria      protein of unknown function LK-Nuclease [Burkholderia phytofirmans PsJN].  
126442572         LK-Nuclease+OST-HTH                                                               BURPS668_A1883         477    Burkholderia pseudomallei 668                                   proteobacteria>betaproteobacteria      hypothetical protein BURPS668_A1883 [Burkholderia pseudomallei  
171057047         LK-Nuclease+OST-HTH                                                               Lcho_0356              160    Leptothrix cholodnii SP-6                                       proteobacteria>betaproteobacteria      hypothetical protein Lcho_0356 [Leptothrix cholodnii SP-6].  
224371038         LK-Nuclease+OST-HTH                                                               HRM2_39800             236    Desulfobacterium autotrophicum HRM2                             proteobacteria>deltaproteobacteria     hypothetical protein HRM2_39800 [Desulfobacterium autotrophicum  
224371902         LK-Nuclease+OST-HTH                                                               HRM2_48560             239    Desulfobacterium autotrophicum HRM2                             proteobacteria>deltaproteobacteria     hypothetical protein HRM2_48560 [Desulfobacterium autotrophicum  
153004596         LK-Nuclease+OST-HTH                                                               Anae109_1734           249    Anaeromyxobacter sp. Fw109-5                                    proteobacteria>deltaproteobacteria     hypothetical protein Anae109_1734 [Anaeromyxobacter sp. Fw109-5].  
108762087         LK-Nuclease+OST-HTH                                                               MXAN_0030              320    Myxococcus xanthus DK 1622                                      proteobacteria>deltaproteobacteria     hypothetical protein MXAN_0030 [Myxococcus xanthus DK 1622].  
149922564         LK-Nuclease+OST-HTH                                                               PPSIR1_41429           264    Plesiocystis pacifica SIR-1                                     proteobacteria>deltaproteobacteria     hypothetical protein PPSIR1_41429 [Plesiocystis pacifica SIR-1].  
46578508          LK-Nuclease+OST-HTH                                                               DVU0091                240    Desulfovibrio vulgaris str. Hildenborough                       proteobacteria>deltaproteobacteria     hypothetical protein DVU0091 [Desulfovibrio vulgaris subsp.  
220918096         LK-Nuclease+OST-HTH                                                               A2cp1_2999             253    Anaeromyxobacter dehalogenans 2CP-1                             proteobacteria>deltaproteobacteria     protein of unknown function LK-Nuclease [Anaeromyxobacter dehalogenans  
197123307         LK-Nuclease+OST-HTH                                                               AnaeK_2905             253    Anaeromyxobacter sp. K                                          proteobacteria>deltaproteobacteria     protein of unknown function LK-Nuclease [Anaeromyxobacter sp. K].  
86159239          LK-Nuclease+OST-HTH                                                               Adeh_2817              253    Anaeromyxobacter dehalogenans 2CP-C                             proteobacteria>deltaproteobacteria     hypothetical protein Adeh_2817 [Anaeromyxobacter dehalogenans  
115377562         LK-Nuclease+OST-HTH                                                               STIAU_6634             359    Stigmatella aurantiaca DW4/3-1                                  proteobacteria>deltaproteobacteria     hypothetical protein STIAU_6634 [Stigmatella aurantiaca DW4/3-1].  
254456978         LK-Nuclease+OST-HTH                                                               CBGD1_673              257    Campylobacterales bacterium GD 1                                proteobacteria>epsilonproteobacteria   protein containing LK-Nuclease [Campylobacterales bacterium GD 1].  
153951620         LK-Nuclease+OST-HTH                                                               JJD26997_0199          232    Campylobacter jejuni subsp. doylei 269.97                       proteobacteria>epsilonproteobacteria   hypothetical protein JJD26997_0199 [Campylobacter jejuni subsp.  
148926354         LK-Nuclease+OST-HTH                                                               Cj8486_0183c           232    Campylobacter jejuni subsp. jejuni CG8486                       proteobacteria>epsilonproteobacteria   hypothetical protein Cj8486_0183c [Campylobacter jejuni subsp.  
86153669          LK-Nuclease+OST-HTH                                                               CJJHB9313_0205         232    Campylobacter jejuni subsp. jejuni HB93-13                      proteobacteria>epsilonproteobacteria   Protein of unknown function family [Campylobacter jejuni subsp.  
86149532          LK-Nuclease+OST-HTH                                                               CJJCF936_0212          232    Campylobacter jejuni subsp. jejuni CF93-6                       proteobacteria>epsilonproteobacteria   conserved hypothetical protein [Campylobacter jejuni subsp. jejuni  
57237194          LK-Nuclease+OST-HTH                                                               CJE0182                232    Campylobacter jejuni RM1221                                     proteobacteria>epsilonproteobacteria   hypothetical protein CJE0182 [Campylobacter jejuni RM1221].  
154148166         LK-Nuclease+OST-HTH                                                               CHAB381_1180           259    Campylobacter hominis ATCC BAA-381                              proteobacteria>epsilonproteobacteria   hypothetical protein CHAB381_1180 [Campylobacter hominis ATCC  
57168636          LK-Nuclease+OST-HTH                                                               CCO1685                232    Campylobacter coli RM2228                                       proteobacteria>epsilonproteobacteria   conserved hypothetical protein [Campylobacter coli RM2228].  
222824458         LK-Nuclease+OST-HTH                                                               Cla_1475               229    Campylobacter lari RM2100                                       proteobacteria>epsilonproteobacteria   hypothetical protein Cla_1475 [Campylobacter lari RM2100].  
152992773         LK-Nuclease+OST-HTH                                                               SUN_1182               249    Sulfurovum sp. NBC37-1                                          proteobacteria>epsilonproteobacteria   hypothetical protein SUN_1182 [Sulfurovum sp. NBC37-1].  
283955380         LK-Nuclease+OST-HTH                                                               C414_000450061         232    Campylobacter jejuni subsp. jejuni 414                          proteobacteria>epsilonproteobacteria   hypothetical protein C414_000450061 [Campylobacter jejuni subsp.  
34557802          LK-Nuclease+OST-HTH                                                               WS1458                 248    Wolinella succinogenes DSM 1740                                 proteobacteria>epsilonproteobacteria   hypothetical protein WS1458 [Wolinella succinogenes DSM 1740].  
283955630         LK-Nuclease+OST-HTH                                                               C1336_000040041        232    Campylobacter jejuni subsp. jejuni 1336                         proteobacteria>epsilonproteobacteria   hypothetical protein C1336_000040041 [Campylobacter jejuni subsp.  
146283893         LK-Nuclease+OST-HTH                                                               PST_3576               243    Pseudomonas stutzeri A1501                                      proteobacteria>gammaproteobacteria     hypothetical protein PST_3576 [Pseudomonas stutzeri A1501].  
47104079          LK-Nuclease+OST-HTH                                                               PBPRC0054              341    Photobacterium profundum SS9                                    proteobacteria>gammaproteobacteria     hypothetical protein PBPRC0054 [Photobacterium profundum SS9].  
114564810         LK-Nuclease+OST-HTH                                                               Sfri_3658              242    Shewanella frigidimarina NCIMB 400                              proteobacteria>gammaproteobacteria     hypothetical protein Sfri_3658 [Shewanella frigidimarina NCIMB  
257455528         LK-Nuclease+OST-HTH                                                               ENHAE0001_0038         350    Enhydrobacter aerosaccus SK60                                   proteobacteria>gammaproteobacteria     conserved hypothetical protein [Enhydrobacter aerosaccus SK60].  
71066424          LK-Nuclease+OST-HTH                                                               Psyc_1869              335    Psychrobacter arcticus 273-4                                    proteobacteria>gammaproteobacteria     hypothetical protein Psyc_1869 [Psychrobacter arcticus 273-4].  
88703638          LK-Nuclease+OST-HTH                                                               KT71_17336             241    Congregibacter litoralis KT71                                   proteobacteria>gammaproteobacteria     conserved hypothetical protein (LK-Nuclease) [Congregibacter litoralis  
237799136         LK-Nuclease+OST-HTH                                                               Psyrpo1_010100009876   264    Pseudomonas syringae pv. oryzae str. 1_6                        proteobacteria>gammaproteobacteria     hypothetical protein Psyrpo1_09876 [Pseudomonas syringae pv. oryzae  
227329498         LK-Nuclease+OST-HTH                                                               PcarcW_010200019989    241    Pectobacterium carotovorum subsp. carotovorum WPP14             proteobacteria>gammaproteobacteria     hypothetical protein PcarcW_19989 [Pectobacterium carotovorum  
258544860         LK-Nuclease+OST-HTH                                                               HMPREF0198_1129        329    Cardiobacterium hominis ATCC 15826                              proteobacteria>gammaproteobacteria     conserved hypothetical protein [Cardiobacterium hominis ATCC  
149376066         LK-Nuclease+OST-HTH                                                               MDG893_03770           248    Marinobacter algicola DG893                                     proteobacteria>gammaproteobacteria     hypothetical protein MDG893_03770 [Marinobacter algicola DG893].  
88799413          LK-Nuclease+OST-HTH                                                               MED297_17443           252    Reinekea blandensis MED297                                      proteobacteria>gammaproteobacteria     hypothetical protein MED297_17443 [Reinekea sp. MED297].  
254514031         LK-Nuclease+OST-HTH                                                               NOR53_2234             254    gamma proteobacterium NOR5-3                                    proteobacteria>gammaproteobacteria     protein containing LK-Nuclease [gamma proteobacterium NOR5-3].  
66046764          LK-Nuclease+OST-HTH                                                               Psyr_3535              264    Pseudomonas syringae pv. syringae B728a                         proteobacteria>gammaproteobacteria     hypothetical protein Psyr_3535 [Pseudomonas syringae pv. syringae  
254507560         LK-Nuclease+OST-HTH                                                               VPMS16_276             240    Vibrio parahaemolyticus 16                                      proteobacteria>gammaproteobacteria     protein containing LK-Nuclease [Vibrio parahaemolyticus 16].  
258546012         LK-Nuclease+OST-HTH                                                               HMPREF0198_2281        265    Cardiobacterium hominis ATCC 15826                              proteobacteria>gammaproteobacteria     conserved hypothetical protein [Cardiobacterium hominis ATCC  
110833552         LK-Nuclease+OST-HTH                                                               ABO_0691               252    Alcanivorax borkumensis SK2                                     proteobacteria>gammaproteobacteria     hypothetical protein ABO_0691 [Alcanivorax borkumensis SK2].  
196155113         LK-Nuclease+OST-HTH                                                               MADE_00290             249    Alteromonas macleodii 'Deep ecotype'                            proteobacteria>gammaproteobacteria     hypothetical protein MADE_00290 [Alteromonas macleodii 'Deep  
237808074         LK-Nuclease+OST-HTH                                                               Tola_1311              239    Tolumonas auensis DSM 9187                                      proteobacteria>gammaproteobacteria     protein of unknown function LK-Nuclease [Tolumonas auensis DSM 9187].  
188992022         LK-Nuclease+OST-HTH                                                               xccb100_2627           266    Xanthomonas campestris pv. campestris str. B100                 proteobacteria>gammaproteobacteria     hypothetical protein xccb100_2627 [Xanthomonas campestris pv.  
78047256          LK-Nuclease+OST-HTH                                                               XCV1700                266    Xanthomonas campestris pv. vesicatoria str. 85-10               proteobacteria>gammaproteobacteria     hypothetical protein XCV1700 [Xanthomonas campestris pv.  
212637199         LK-Nuclease+OST-HTH                                                               swp_4494               245    Shewanella piezotolerans WP3                                    proteobacteria>gammaproteobacteria     hypothetical protein swp_4494 [Shewanella piezotolerans WP3].  
146291809         LK-Nuclease+OST-HTH                                                               Sputcn32_0704          268    Shewanella putrefaciens CN-32                                   proteobacteria>gammaproteobacteria     hypothetical protein Sputcn32_0704 [Shewanella putrefaciens CN-32].  
117922000         LK-Nuclease+OST-HTH                                                               Shewana3_3564          268    Shewanella sp. ANA-3                                            proteobacteria>gammaproteobacteria     hypothetical protein Shewana3_3564 [Shewanella sp. ANA-3].  
21244544          LK-Nuclease+OST-HTH                                                               XAC3820                268    Xanthomonas axonopodis pv. citri str. 306                       proteobacteria>gammaproteobacteria     hypothetical protein XAC3820 [Xanthomonas axonopodis pv. citri str.  
262375022         LK-Nuclease+OST-HTH                                                               HMPREF0017_00177       269    Acinetobacter lwoffii SH145                                     proteobacteria>gammaproteobacteria     conserved hypothetical protein [Acinetobacter lwoffii SH145].  
146308706         LK-Nuclease+OST-HTH                                                               Pmen_3691              272    Pseudomonas mendocina ymp                                       proteobacteria>gammaproteobacteria     hypothetical protein Pmen_3691 [Pseudomonas mendocina ymp].  
78049501          LK-Nuclease+OST-HTH                                                               XCV3945                273    Xanthomonas campestris pv. vesicatoria str. 85-10               proteobacteria>gammaproteobacteria     hypothetical protein XCV3945 [Xanthomonas campestris pv.  
260914714         LK-Nuclease+OST-HTH                                                               HMPREF0621_2040        261    Pasteurella dagmatis ATCC 43325                                 proteobacteria>gammaproteobacteria     conserved hypothetical protein [Pasteurella dagmatis ATCC 43325].  
256822960         LK-Nuclease+OST-HTH                                                               Kkor_1743              236    Kangiella koreensis DSM 16069                                   proteobacteria>gammaproteobacteria     protein of unknown function LK-Nuclease [Kangiella koreensis DSM 16069].  
237800598         LK-Nuclease+OST-HTH                                                               Psyrpo1_010100017333   274    Pseudomonas syringae pv. oryzae str. 1_6                        proteobacteria>gammaproteobacteria     hypothetical protein Psyrpo1_17333 [Pseudomonas syringae pv. oryzae  
187940199         LK-Nuclease+OST-HTH                                                               PACL_0668              274    Pseudomonas aeruginosa                                          proteobacteria>gammaproteobacteria     hypothetical protein PACL_0668 [Pseudomonas aeruginosa].  
58583915          LK-Nuclease+OST-HTH                                                               XOO4292                274    Xanthomonas oryzae pv. oryzae KACC10331                         proteobacteria>gammaproteobacteria     hypothetical protein XOO4292 [Xanthomonas oryzae pv. oryzae  
52425038          LK-Nuclease+OST-HTH                                                               MS0983                 274    Mannheimia succiniciproducens MBEL55E                           proteobacteria>gammaproteobacteria     hypothetical protein MS0983 [Mannheimia succiniciproducens  
254427448         LK-Nuclease+OST-HTH                                                               ADG881_678             235    Alcanivorax sp. DG881                                           proteobacteria>gammaproteobacteria     hypothetical protein ADG881_678 [Alcanivorax sp. DG881].  
52842281          LK-Nuclease+OST-HTH                                                               lpg2067                260    Legionella pneumophila subsp. pneumophila str. Philadelphia 1   proteobacteria>gammaproteobacteria     hypothetical protein lpg2067 [Legionella pneumophila subsp.  
220934364         LK-Nuclease+OST-HTH                                                               Tgr7_1190              275    Thioalkalivibrio sp. HL-EbGR7                                   proteobacteria>gammaproteobacteria     protein of unknown function LK-Nuclease [Thioalkalivibrio sp. HL-EbGR7].  
188574761         LK-Nuclease+OST-HTH                                                               PXO_03707              276    Xanthomonas oryzae pv. oryzae PXO99A                            proteobacteria>gammaproteobacteria     hypothetical protein PXO_03707 [Xanthomonas oryzae pv. oryzae  
269925423         LK-Nuclease+OST-HTH                                                               Tter_0302              408    Thermobaculum terrenum ATCC BAA-798                             unclassified Bacteria                  protein of unknown function LK-Nuclease [Thermobaculum terrenum ATCC  
225023600         LK-Nuclease+OST-HTH+OST-HTH                                                       EIKCOROL_00460         392    Eikenella corrodens ATCC 23834                                  proteobacteria>betaproteobacteria      hypothetical protein EIKCOROL_00460 [Eikenella corrodens ATCC  
255065206         LK-Nuclease+OST-HTH+OST-HTH                                                       NEISICOT_00196         390    Neisseria sicca ATCC 29256                                      proteobacteria>betaproteobacteria      conserved hypothetical protein [Neisseria sicca ATCC 29256].  
261364990         LK-Nuclease+OST-HTH+OST-HTH                                                       NmucA2_020100007031    390    Neisseria mucosa ATCC 25996                                     proteobacteria>betaproteobacteria      hypothetical protein NmucA2_07031 [Neisseria mucosa ATCC 25996].  
268611850         LK-Nuclease+OST-HTH+OST-HTH                                                       RflaF_010100020381     303    Ruminococcus flavefaciens FD-1                                  firmicutes                             hypothetical protein RflaF_20381 [Ruminococcus flavefaciens FD-1].  
266625430         LK-Nuclease+OST-HTH+OST-HTH                                                       ChatD1_010100032463    306    Clostridium hathewayi DSM 13479                                 firmicutes                             hypothetical protein ChatD1_32463 [Clostridium hathewayi DSM  
257454371         LK-Nuclease+OST-HTH+OST-HTH                                                       ENHAE0001_0502         361    Enhydrobacter aerosaccus SK60                                   proteobacteria>gammaproteobacteria     conserved hypothetical protein [Enhydrobacter aerosaccus SK60].  
239623453         LK-Nuclease+OST-HTH+OST-HTH                                                       CBFG_05062             308    Clostridiales bacterium 1_7_47FAA                               firmicutes                             conserved hypothetical protein [Clostridiales bacterium  
160935678         LK-Nuclease+OST-HTH+OST-HTH                                                       CLOBOL_00568           308    Clostridium bolteae ATCC BAA-613                                firmicutes                             hypothetical protein CLOBOL_00568 [Clostridium bolteae ATCC  
225389922         LK-Nuclease+OST-HTH+OST-HTH                                                       CLOSTASPAR_03672       232    Clostridium asparagiforme DSM 15981                             firmicutes                             hypothetical protein CLOSTASPAR_03672 [Clostridium asparagiforme  
153815213         LK-Nuclease+OST-HTH+OST-HTH                                                       RUMTOR_01447           323    Ruminococcus torques ATCC 27756                                 firmicutes                             hypothetical protein RUMTOR_01447 [Ruminococcus torques ATCC  
163815163         LK-Nuclease+OST-HTH+OST-HTH                                                       COPEUT_01321           322    Coprococcus eutactus ATCC 27759                                 firmicutes                             hypothetical protein COPEUT_01321 [Coprococcus eutactus ATCC  
238917756         LK-Nuclease+OST-HTH+OST-HTH                                                       EUBELI_01837           318    Eubacterium eligens ATCC 27750                                  firmicutes                             hypothetical protein EUBELI_01837 [Eubacterium eligens ATCC 27750].  
256826985         LK-Nuclease+OST-HTH+OST-HTH                                                       Ccur_05410             312    Cryptobacterium curtum DSM 15641                                actinobacteria                         Protein of unknown function LK-Nuclease [Cryptobacterium curtum DSM  
167766019         LK-Nuclease+OST-HTH+OST-HTH                                                       CLOSS21_00511          319    Clostridium sp. SS2/1                                           firmicutes                             hypothetical protein CLOSS21_00511 [Clostridium sp. SS2/1].  
154506256         LK-Nuclease+OST-HTH+OST-HTH                                                       RUMGNA_03798           320    Ruminococcus gnavus ATCC 29149                                  firmicutes                             hypothetical protein RUMGNA_03798 [Ruminococcus gnavus ATCC 29149].  
238059538         LK-Nuclease+OST-HTH+OST-HTH                                                       MCAG_00504             367    Micromonospora sp. ATCC 39149                                   actinobacteria                         hypothetical protein MCAG_00504 [Micromonospora sp. ATCC 39149].  
21673303          LK-Nuclease+OST-HTH                                                               CT0467                 426    Chlorobium tepidum TLS                                          bacteroidetes/chlorobi                 hypothetical protein CT0467 [Chlorobium tepidum TLS].  
15639879          LK-Nuclease+OST-HTH+S1                                                            TP0894                 340    Treponema pallidum subsp. pallidum str. Nichols                 spirochaetes                           hypothetical protein TP0894 [Treponema pallidum subsp. pallidum  
42528036          LK-Nuclease+OST-HTH+S1                                                            TDE2536                313    Treponema denticola ATCC 35405                                  spirochaetes                           hypothetical protein TDE2536 [Treponema denticola ATCC 35405].  
161789278         LK-Nuclease+OST-HTH+S1                                                            BMSF_0033              297    Vibrio sp. 09022                                                proteobacteria>gammaproteobacteria     hypothetical protein BMSF_0033 [Vibrio sp. 09022].  
25809033          LK-Nuclease+OST-HTH+S1                                                            -                      306    Gram-negative bacterium 0471                                    unclassified Bacteria                  hypothetical protein [Gram-negative bacterium 0471].  
149908210         LK-Nuclease+OST-HTH+S1                                                            PE36_01852             304    Moritella sp. PE36                                              proteobacteria>gammaproteobacteria     hypothetical protein PE36_01852 [Moritella sp. PE36].  
257456927         LK-Nuclease+OST-HTH+S1                                                            TREVI0001_1869         311    Treponema vincentii ATCC 35580                                  spirochaetes                           conserved hypothetical protein [Treponema vincentii ATCC 35580].  
# 7;  
56476823          OST-HTH                                                                           ebA2464                314    Aromatoleum aromaticum EbN1                                     proteobacteria>betaproteobacteria      hypothetical protein ebA2464 [Aromatoleum aromaticum EbN1].  
270491449         OST-HTH                                                                           AcavDRAFT_0259         308    Acidovorax avenae subsp. avenae ATCC 19860                      proteobacteria>betaproteobacteria      conserved hypothetical protein [Acidovorax avenae subsp. avenae  
28867250          OST-HTH                                                                           PSPTO_0007             294    Pseudomonas syringae pv. tomato str. DC3000                     proteobacteria>gammaproteobacteria     hypothetical protein PSPTO_0007 [Pseudomonas syringae pv. tomato  
237653824         OST-HTH                                                                           Tmz1t_3165             293    Thauera sp. MZ1T                                                proteobacteria>betaproteobacteria      hypothetical protein Tmz1t_3165 [Thauera sp. MZ1T].  
146279554         OST-HTH                                                                           Rsph17025_3537         286    Rhodobacter sphaeroides ATCC 17025                              proteobacteria>alphaproteobacteria     exonuclease, RNase T and DNA polymerase III [Rhodobacter  
217969422         OST-HTH                                                                           Tmz1t_0994             286    Thauera sp. MZ1T                                                proteobacteria>betaproteobacteria      hypothetical protein Tmz1t_0994 [Thauera sp. MZ1T].  
83954751          OST-HTH                                                                           NAS141_16058           278    Sulfitobacter sp. NAS-14.1                                      proteobacteria>alphaproteobacteria     hypothetical protein NAS141_16058 [Sulfitobacter sp. NAS-14.1].  
# 2;  
163815973         OST-HTH                                                                           COPEUT_02153           320    Coprococcus eutactus ATCC 27759                                 firmicutes                             hypothetical protein COPEUT_02153 [Coprococcus eutactus ATCC  
160894758         OST-HTH                                                                           CLOL250_02309          291    Clostridium sp. L2-50                                           firmicutes                             hypothetical protein CLOL250_02309 [Clostridium sp. L2-50].  
# 2;  
23016669          LK-Nuclease+OST-HTH                                                               Magn03011123           188    Magnetospirillum magnetotacticum MS-1                           proteobacteria>alphaproteobacteria     hypothetical protein Magn03011123 [Magnetospirillum magnetotacticum  
83309768          LK-Nuclease+OST-HTH                                                               amb0669                188    Magnetospirillum magneticum AMB-1                               proteobacteria>alphaproteobacteria     hypothetical protein amb0669 [Magnetospirillum magneticum AMB-1].  
# 2;  
238485334         OST-HTH                                                                           AFLA_023940            126    Aspergillus flavus NRRL3357                                     fungi>ascomycota                       hypothetical protein AFLA_023940 [Aspergillus flavus NRRL3357].  
192362040         OST-HTH                                                                           CJA_3180               111    Cellvibrio japonicus Ueda107                                    proteobacteria>gammaproteobacteria     hypothetical protein CJA_3180 [Cellvibrio japonicus Ueda107].  
# 2;  
262187336         OST-HTH                                                                           AmarD1_010100011624    107    Aeromicrobium marinum DSM 15272                                 actinobacteria                         hypothetical protein AmarD1_11624 [Aeromicrobium marinum DSM  
254823311         OST-HTH                                                                           MintA_010100025509     91     Mycobacterium intracellulare ATCC 13950                         actinobacteria                         hypothetical protein MintA_25509 [Mycobacterium intracellulare ATCC  
# 1;  
260654181         OST-HTH                                                                           GCWU000246_00092       704    Jonquetella anthropi E3_33 E1                                   synergistetes                          conserved hypothetical protein [Jonquetella anthropi E3_33 E1].  
254421342         OST-HTH                                                                           S7335_1492             551    Synechococcus sp. PCC 7335                                      cyanobacteria                          hypothetical protein S7335_1492 [Synechococcus sp. PCC 7335].  
# 1;  
149917268         LK-Nuclease+OST-HTH                                                               PPSIR1_40335           474    Plesiocystis pacifica SIR-1                                     proteobacteria>deltaproteobacteria     hypothetical protein PPSIR1_40335 [Plesiocystis pacifica SIR-1].  
212692683         OST-HTH+S1+MNS                                                                    BACDOR_02181           1701   Bacteroides dorei DSM 17855                                     bacteroidetes/chlorobi                 hypothetical protein BACDOR_02181 [Bacteroides dorei DSM 17855].  
115397957         ZNF+LK-Nuclease+OST-HTH                                                           ATEG_05392             885    Aspergillus terreus NIH2624                                     fungi>ascomycota                       conserved hypothetical protein [Aspergillus terreus NIH2624].  
205356564         LK-Nuclease+OST-HTH                                                               Cj8421_0189            683    Campylobacter jejuni subsp. jejuni CG8421                       proteobacteria>epsilonproteobacteria   hypothetical protein Cj8421_0189 [Campylobacter jejuni subsp.  
46580394          LK-Nuclease+OST-HTH                                                               DVU1985                418    Desulfovibrio vulgaris str. Hildenborough                       proteobacteria>deltaproteobacteria     hypothetical protein DVU1985 [Desulfovibrio vulgaris subsp.  
196255379         OST-HTH                                                                           Cyan7822DRAFT_1073     359    Cyanothece sp. PCC 7822                                         cyanobacteria                          hypothetical protein Cyan7822DRAFT_1073 [Cyanothece sp. PCC 7822].  
156602934         RNA-Helicase_SFI+LK-Nuclease+OST-HTH                                              NEMVEDRAFT_v1g224852   343    Nematostella vectensis                                          metazoa>cnidaria                       hypothetical protein NEMVEDRAFT_v1g224852 [Nematostella vectensis].  
254415491         LK-Nuclease+OST-HTH                                                               MC7420_4949            265    Microcoleus chthonoplastes PCC 7420                             cyanobacteria                          hypothetical protein MC7420_4949 [Microcoleus chthonoplastes PCC  
153874870         OST-HTH                                                                           BGP_5435               230    Beggiatoa sp. PS                                                proteobacteria>gammaproteobacteria     hypothetical protein BGP_5435 [Beggiatoa sp. PS].  
210633139         OST-HTH                                                                           COLSTE_01619           110    Collinsella stercoris DSM 13279                                 actinobacteria                         hypothetical protein COLSTE_01619 [Collinsella stercoris DSM  
281306909         OST-HTH                                                                           -                      105    Nitrosomonas europaea                                           proteobacteria>betaproteobacteria      Chain A, Solution Nmr Structure Of Uncharacterized Protein From  
121609648         OST-HTH                                                                           Veis_2695              85     Verminephrobacter eiseniae EF01-2                               proteobacteria>betaproteobacteria      hypothetical protein Veis_2695 [Verminephrobacter eiseniae EF01-2].  
52548607          OST-HTH                                                                           GZ17F1_27              64     uncultured archaeon GZfos17F1                                                                          hypothetical protein GZ17F1_27 [uncultured archaeon GZfos17F1].
```

  
Top
